# Global, Regional, and National Change Patterns in the Incidence of Low Back Pain From 1990 to 2019 and Its Predicted Level in the Next Decade

**DOI:** 10.3389/ijph.2024.1606299

**Published:** 2024-02-21

**Authors:** Yong Gu, Zhengming Wang, Haiwei Shi, Yanlin He, Yunshang Yang, Yajun Li, Shuangshuang Chen, Zhirong Wang, Yubo Mei, Long Xiao

**Affiliations:** ^1^ Translational Medical Innovation Center, Zhangjiagang TCM Hospital Affiliated to Nanjing University of Chinese Medicine, Zhangjiagang, China; ^2^ Department of Orthopedics, Zhangjiagang TCM Hospital Affiliated to Nanjing University of Chinese Medicine, Zhangjiagang, China; ^3^ Shi’s Center of Orthopedics and Traumatology, Shuguang Hospital Affiliated to Shanghai University of Traditional Chinese Medicine, Shanghai, China; ^4^ Key Laboratory of Carbohydrate Chemistry and Biotechnology, Ministry of Education, School of Biotechnology, Jiangnan University, Wuxi, China

**Keywords:** incidence, burden, age, low back pain, socio-demographic index

## Abstract

**Objectives:** To analyze and describe the spatiotemporal trends of Low back pain (LBP) burdens from 1990 to 2019 and anticipate the following decade’s incidence.

**Methods:** Using data from the Global Burden of Disease (GBD) 2019 Study, we described net drifts, local drifts, age effects, and period cohort effects in incidence and forecasted incidence rates and cases by sex from 2020 to 2029 using the Nordpred R package.

**Results:** LBP remained the leading cause of the musculoskeletal disease burden globally and across all socio-demographic index (SDI) regions. China is the top country. For recent periods, high-SDI countries faced unfavorable or worsening risks. The relative risk of incidence showed improving trends over time and in successively younger birth cohorts amongst low-middle-, middle- and high-middle-SDI countries. Additionally, the age-standardized incidence rates (ASIR) of LBP in both sexes globally showed a decreasing trend, but the incident cases would increase from 223 to 253 million overall in the next decade.

**Conclusion:** As the population ages, incident cases will rise but ASIR will fall. To minimise LBP, public awareness and disease prevention and control are needed.

## Introduction

Low back pain (LBP) is a common symptom in populations globally, occurring in all age groups [1–3], and is the leading cause of disability [4]. LBP ranks ninth in disability-adjusted life years (DALYs) and highest in years lived with disability (YLDs), accounting for 2.5% of total DALYs and 7.41% of total YLDs, respectively, in 2019 [5]. With population growth and aging, the number of people with disabilities due to LBP is rapidly increasing, particularly in low-income and middle-income countries [6], where health and social systems are poorly equipped to deal with this growing burden.

LBP has multiple causes and may be related to various factors, including symptom-related, lifestyle, psychological, and social factors [7]. However, in most cases, the exact cause of pain cannot be determined. Therefore, measures must be taken to minimize modifiable risk factors such as smoking and obesity, which are associated with an increased risk of LBP [8]. Untreated pain can lead to depression, functional impairment, sleep disturbance, late recovery, malnutrition, and cognitive dysfunction [9]. Additionally, LBP is the leading cause of premature labor market exit in adults [10]. The societal impact of early retirement in terms of absenteeism or productivity loss costs is substantial [7]. LBP can lead to increased costs to businesses, governments, and society. Therefore, an investigation of the global epidemic trends of LBP is urgently needed to facilitate the development of health policies to guide the prevention and management thereof.

Recent studies report the incidence, prevalence, and YLDs of LBP using data from the Global Burden of Disease (GBD) study [11–13]. However, this traditional descriptive analysis method fails to distinguish the relative contributions of age, period, and cohort effects on incidence. Analyzing these effects on overall time trends help determine the effectiveness of early policy interventions in the health system and future goals [14–16]. We aimed to estimate global, regional, and national epidemiological patterns of LBP based on improved methods, and provide valuable insights to support decision making and suggests actions to reduce the disease burden of LBP. We also focused on secular trends in the global LBP burden over the next 10 years for the first time, with the aim of conducting an evidence-based assessment of the efficacy of current prevention and therapeutic strategies to reduce the global LBP burden.

## Methods

### Data Source

The Global Burden of Disease Study is the largest and most comprehensive study to measure the epidemiological levels and trends worldwide [5]. The GBD study used a Bayesian meta-regression tool, DisMod-MR V.2.1, to pool the heterogeneous data. Detailed information about data processing and modeling methods related to LBP is available in Online Supplementary Appendix S1 of the GBD study 2019 [5].

The data sources used to estimate the burden of LBP around the world can be found through the Global Health Data Exchange query tool [17]. The SDI is a composite indicator of development status that is strongly correlated with health outcomes, estimated based on the total fertility rate, educational attainment, and lag distributed income. Human Development Index (HDI) data were obtained from the Human Development Report 2019 [18]. The available HDI data can be matched with incidence among 187 countries and territories.

### Statistical Analysis

Overall temporal trends in incidence were assessed by the all-age incidence and age-standardized incidence, as well as the relative percent change of incidence between 1990 and 2019. Incident cases were divided into five age groups: 5–19, 20–44, 45–59, 60–74, and >75 years, and the proportion of incidence in each age group was calculated.

The age-period-cohort (APC) model framework was used to analyze potential trends in incidence by age, period, and birth cohort [19]. The detailed introduction and parameter settings for the APC model can be found in the Methods section of the Supplementary Material.

We also performed correlation analyses between net drifts and the SDI/HDI in 2019. Additionally, a hierarchy cluster analysis was conducted to group the countries and territories into four categories (I: significant decrease, II: minor decrease, III: remained stable or minor increase, IV: significant increase) in terms of their temporal trends in incidence. In addition, we predicted the incidence rates and incident cases of LBP from 2020 to 2029 by sex based on an APC model using the Nordpred R package, taking into account changes in the rate of change and population structure [20]. To facilitate a comparison with predicted outcomes, we calculated the absolute number of new cases that would occur if the rate remained stable (baseline reference), decreased by 1% per year (optimistic reference), and increased by 1% per year (pessimistic reference) based on actual observed rates in 2019. A Bayesian age-period-cohort (BAPC) model using an integrated nested Laplace approximation (INLA) was performed to verify the stability of the predicted results. The INLA and BAPC R packages were used for the sensitivity analyses. All data analyses were performed using R software (version 4.1.3, R Core Team).

## Results

### Musculoskeletal (MSK) Disorders Globally, 1990–2019

**FIGURE 1:**
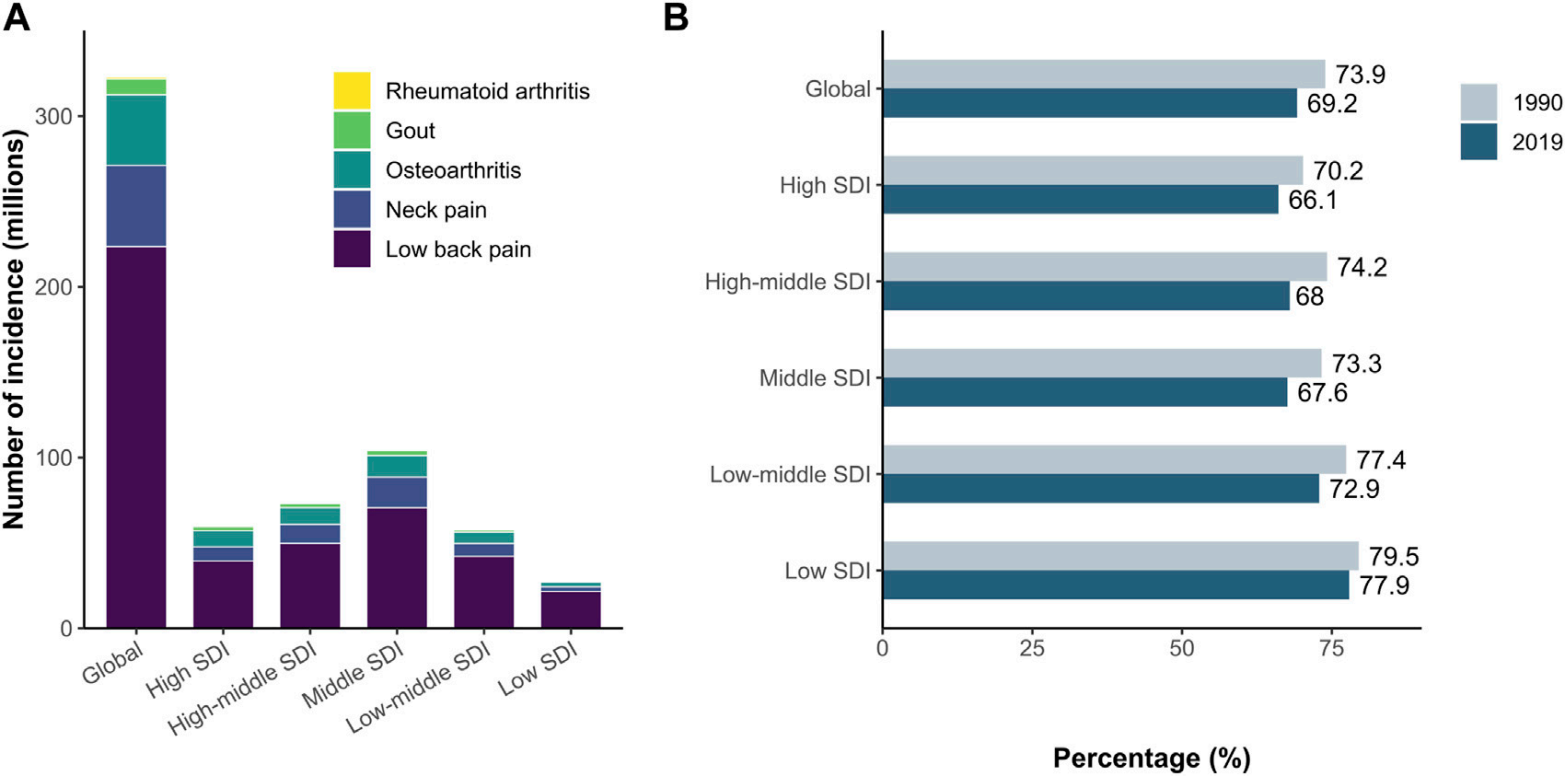
LBP is the leading cause of global incidence from MSK disorders **(A)** The number of incident cases in 2019 for MSK disorders. Globally and regionally, low back pain (LBP) accounts for the largest proportion of incident cases from MSK disorders. **(B)** Change in the proportion of incidence from LBP relative to all MSK disorders incidence between 1990 and 2019. LBP, low back pain; MSK, musculoskeletal (Global Burden of Diseases Study, 21 Global Burden of Diseases regions, 1990–2019).

Globally, the estimated number of incident cases of MSK disorders was 322.75 million (95% UI 292.67–354.31) in 2019 (Figure 1A). Among all causes of MSK disorders, LBP accounted for >60% of the incidence [223.46 million (95% UI 197.71 to 252.99)], resulting in the highest proportion of disease burden from MSK disorders globally and across all SDI regions.

From 1990 to 2019, the proportion of LBP incident cases relative to all MSK disorders decreased from 73.9% to 69.2% globally. The proportion of LBP incident cases decreased across all SDI regions, with the highest relative decrease in the high-middle SDI region (Figure 1B). LBP resulted in the least pronounced proportional reduction in the low-SDI region.

### Global and Regional Trends in LBP Incidence, 1990–2019

Over the past 30 years, the number of new LBP cases has increased from 149.29 million (95% UI 131.31–169.17) to 223.46 million (95% UI 197.71–252.99) by 49.67% (95% UI 45.58%–53.34%), as the global population increased from 53.5 billion (95% UI 52.4–54.6) to 77.4 billion (95% UI 74.8–79.9) with an increase of 44.6%. Globally, the all-age incidence rate of LBP was 2887.97 (95% UI 2555.23–3269.71) per 100,000 population in 2019, a growth of 3.49% (95% UI 0.66–6.02), but the age-standardized incidence rate (ASIR) in 2019 was 2748.9 (95% UI 2425.77–3106.89) per 100,000 population, a 13.25% (95% UI -13.87 to −12.64) decline from 1990.

The percentage change in the number of incident cases substantially increased across all SDI regions between 1990 and 2019, with the highest increase [100.62% (95% UI 98.84 to 102.47)] in the low-SDI regions, which has approximately doubled, and the lowest increase in the high-middle-SDI regions [29.75% (95% UI 25.84 to 33.4)]. From 1990 to 2019, the middle-SDI, low-middle-SDI, and low-SDI regions accounted for an increasing proportion of new LBP cases worldwide. Except for the decrease [−6.13% (95% UI −6.96 to −5.26)] in low-SDI regions, the all-age incidence rates increased relatively in all SDI regions, among which the highest was in middle-SDI regions [8.75% (95% UI 4.65 to 12.67)] and the lowest was in high-middle-SDI regions [4.36% (95% UI 1.21 to 7.29)]. Conversely, the percentage change in ASIRs decreased in all SDI regions. The all-age incidence rate is generally lower than the age-standardized rate in SDI regions, except in low SDI and low-middle SDI regions, which indicates that the all-age incidence rate is more suitable to capture the real burden of LBP in low- and middle-income countries.

**FIGURE 2:**
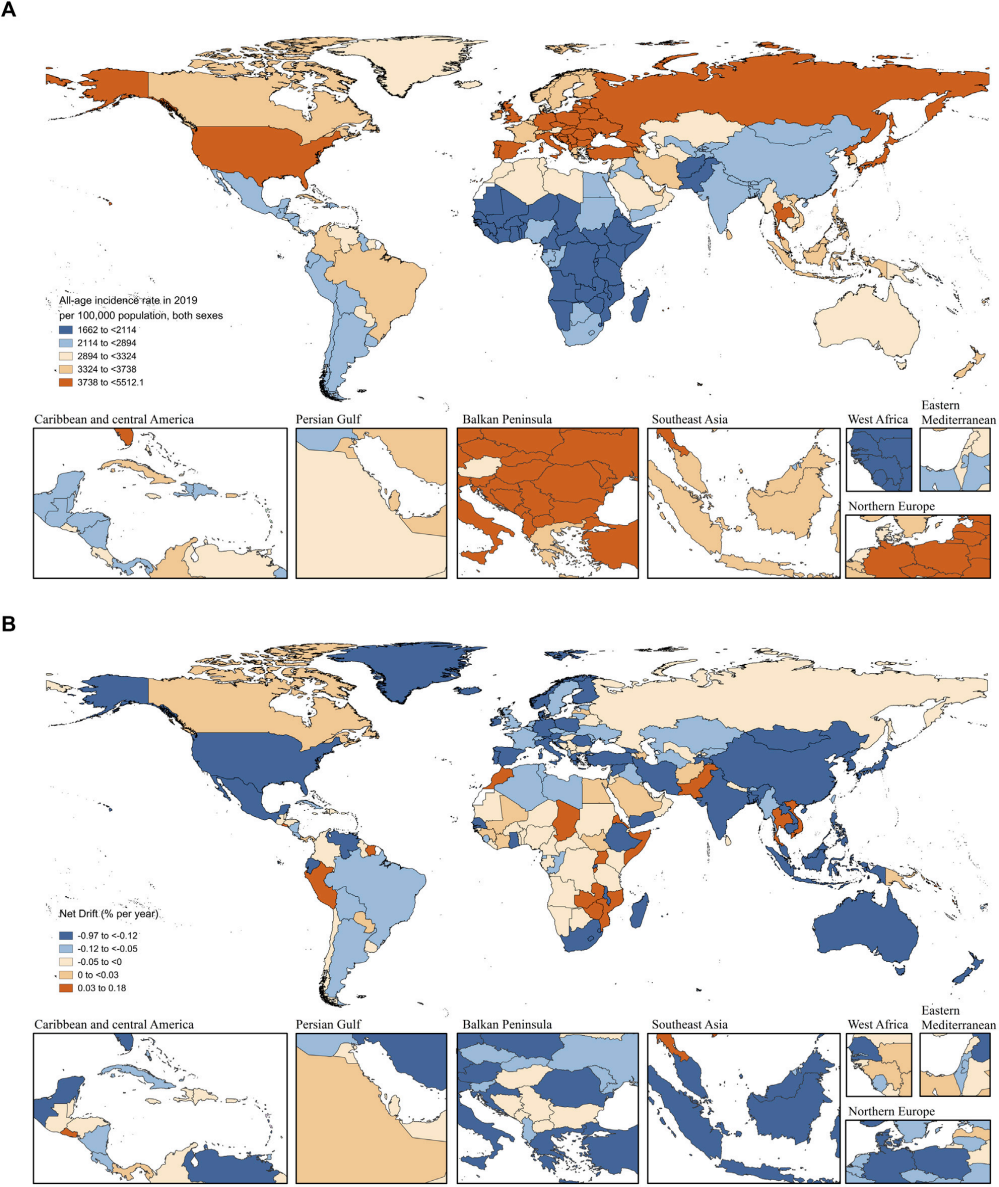
All-age incidence rate in 2019 and net drift of incidence between 1990 and 2019 for LBP in 204 countries and territories **(A)** World map of all-age incidence rates of LBP per 100,000 population for both sexes in 2019. **(B)** World map of net drifts for LBP incidence between 1990 and 2019 (i.e., estimated annual percentage change of incidence from age-period-cohort model). Net drift captures components of the trends attributable to calendar time and successive birth cohorts. LBP, low back pain (Global Burden of Diseases Study, 204 countries or territories, 2019).

Globally, a net drift of −0.37% (95% CI −0.40 to −0.34) per year in incidence was derived from the APC model estimates, ranging from −0.48% (95% CI −0.54 to −0.41) per year in low-middle SDI regions to −0.17% (95% CI −0.19 to −0.14) per year in high SDI regions. That is, in the past 30 years, the incidence rates of LBP overall and across all SDI regions have shown a decreasing trend (Supplementary Tables S2, S3; Figure 2; Supplementary Figure S1).

### National Trends in LBP Incidence, 1990–2019

Of 204 countries and territories, 40 had more than 1 million cases of LBP in 2019. China [40.16 million (95% UI 35.37 to 45.81)], India [29.63 million (95% UI 26.01 to 33.74)] and the United States [13.73 million (95% UI 12.30 to 15.29)] were the top three countries, accounting for 37.40% of the global incident cases of LBP. Thirty-six countries showed a moderate increasing trend (net drifts ≥0.0% per year), with Kiribati [0.17% (95% CI −0.26 to 0.61)], Zambia [0.16% (95% CI 0.11 to 0.20)], and Zimbabwe [0.15% (95% CI 0.12 to 0.19)] being the top three. A total of 168 countries and territories showed a decreasing trend in the incidence rate (net drifts <0·0% per year), and the top three countries were India [−0.97% (95% CI −1.24% to −0.70)], Georgia [−0.73% (95% CI −0.80% to −0.66%)], and China [−0.68% (95% CI −0.76% to −0.60%)] (Supplementary Table S2).

Thailand had the highest increase in the all-age incidence rate [52.93% (95% CI 42.76 to 65.12)] with a net drift of 0.099% per year (95% CI 0.082 to 0.115 per year) in incidence rate. In 2019, 25 countries had all-age incidence rates more than 1.5 times higher than the global level, and most of them were countries with high SDI in Southeast, Central, and Eastern Europe. The ASIRs in three countries (Poland, Vanuatu, and Romania) were more than 1.5 times higher than the global level.

While decreasing net drifts across all SDI regions suggest a favorable incidence reduction of LBP, in some low-SDI countries, such as Afghanistan, Angola, and Niger, the population increased substantially (235.21%, 192.09%, and 190.36%, respectively), and the number of incident cases increased significantly (199.89%, 191.27%, and 183.63%, respectively). Similarly, some countries with high SDI, such as Qatar, the United Arab Emirates, and Saudi Arabia, showed dramatic growth in incident cases (676.05%, 605.35%, and 216.33%) and all-age incidence rates (20.59%, 42.88%, and 42.05%), with net drifts in incidence at −0.03% (−0.34 to 0.28), −0.05% (−0.33 to 0.23), and 0.00% (−0.02–0.03), respectively.

Moreover, populous countries such as India and China, which represent typical emerging economies and belong to BRICS, showed a remarkable decrease in net drifts in incidence, ranking in the top three worldwide [India: −0.97% (−1.24 to −0.70); China: −0.68% (−0.76 to −0.60)] with different patterns of percentage change in all-age incidence rates at −6.47% (−9.83 to −3.16) and 0.57% (−5 to −5.63), respectively. We classified the 204 countries and territories into four categories using hierarchical clustering based on changing trends in incidence (Supplementary Figure S2).

These results indicate that the changing trends of LBP incidence were uneven among countries and were not necessarily consistent with expectations based on SDI at the national level. In addition, the changing direction of incidence captured from traditional indicators (such as all-age incidence rate and ASIR) may not be completely consistent with that indicated by net drifts derived from the APC model, which suggests that it is necessary to distinguish period and cohort effects.

### Time Trends in LBP Incidence Across Different Age Groups

**FIGURE 3:**
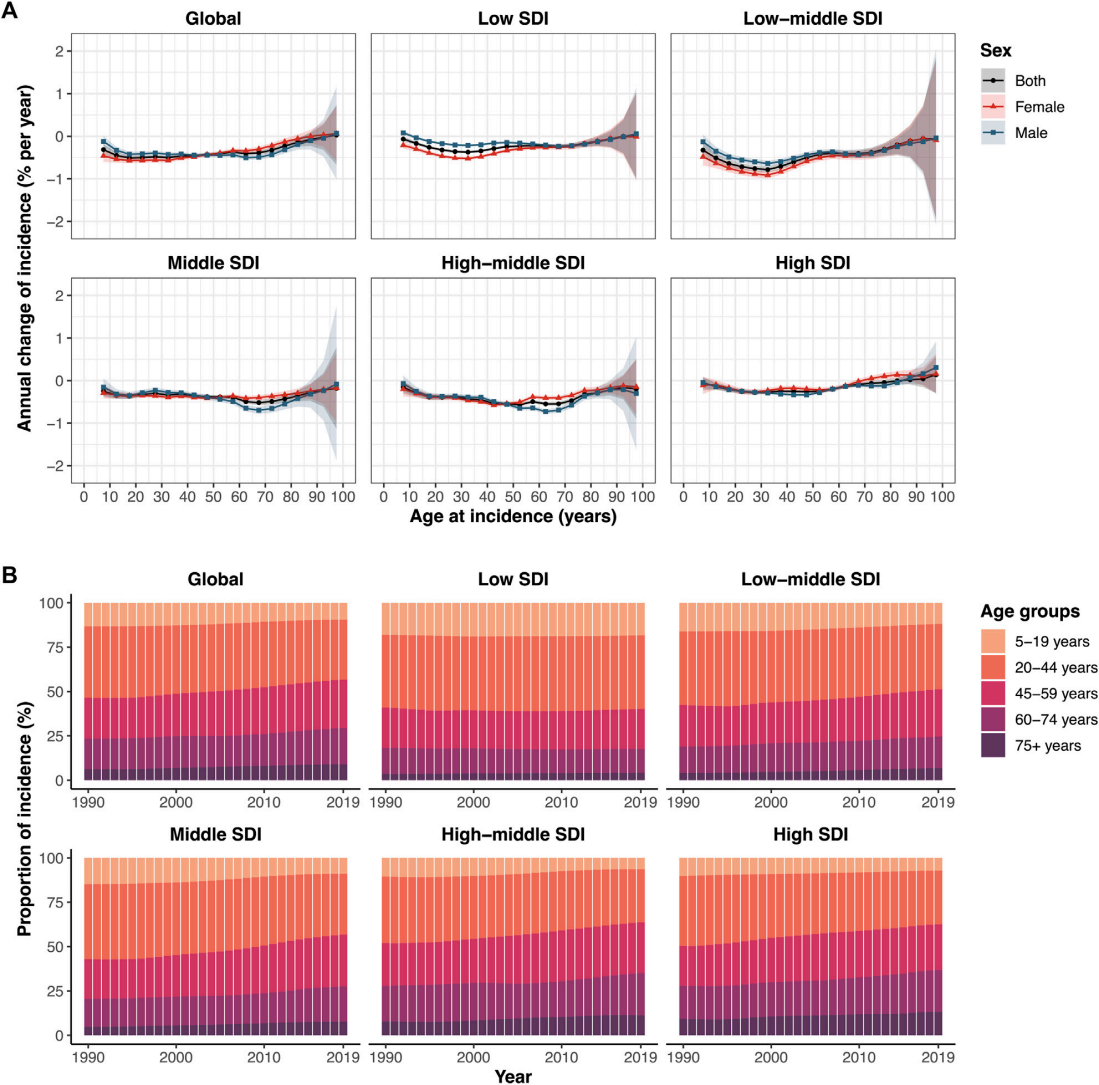
Local drifts of LBP incidence and age distribution of incident cases from LBP by SDI quintiles from 1990 to 2019. **(A)** Annual percentage change in incidence rate of low back pain for 19 age groups (from 5–9 to >95 years) between 1990 and 2019 (i.e., local drifts of LBP incidence estimated from APC model and capturing trends in birth cohort effects). The dots and shaded areas indicate the annual percentage change of incidence (% per year) and the corresponding 95% CIs. **(B)** Temporal change in the relative proportion of incident cases across age groups (5–19 years, 20–44 years, 45–59 years, 60–74 years, >75 years) from 1990 to 2019. LBP, low back pain; SDI, Socio-demographic index (Global Burden of Diseases Study, 21 Global Burden of Diseases regions, 1990–2019).

Globally, the incidence rates of LBP in all age groups showed decreasing trends (***p* < 0.0001**), except for no significant change in the >95 years age group [local drifts: 0.03% (95% CI −0.67 to 0.73) per year]. The steepest incidence reduction occurred in the 15–19 years age group [−0.51% (95% CI −0.57 to −0.45) per year], and the decreasing trend weakened with age. For those under 45 years of age, the incidence of LBP in women declined faster than that in men, whereas the opposite was true for those older than 50 years. Similar trends were observed across the different SDI quintiles. Except for the high SDI region, the incidence rates in all regions improved. In the population over 70 years of age, the incidence of women in countries with high SDI has been increasing (Figure 3A). The local drift of incidence for each country or territory is shown in Supplementary Figures S3–S7.

Globally, the incidence of LBP has shifted from the pediatric population (5–19 years) and young population (20–44 years) to the middle-aged population (45–59 years) and elderly population (>60 years) (Figure 3B) (Supplementary Figures S8–S12). This trend was more obvious in regions with middle SDI, high-middle SDI, and high SDI, but not in regions with low SDI. More than 50% of incident cases were concentrated in the young population group. In regions with a higher SDI, the incidence of LBP in the middle-aged and elderly groups was higher. Especially for the >75 years age group, with the increase in SDI, the relative proportion of LBP incidence also increased. These results indirectly reflect the age trends in the LBP population.

### Age, Period, and Cohort Effects on LBP Incidence

**FIGURE 4:**
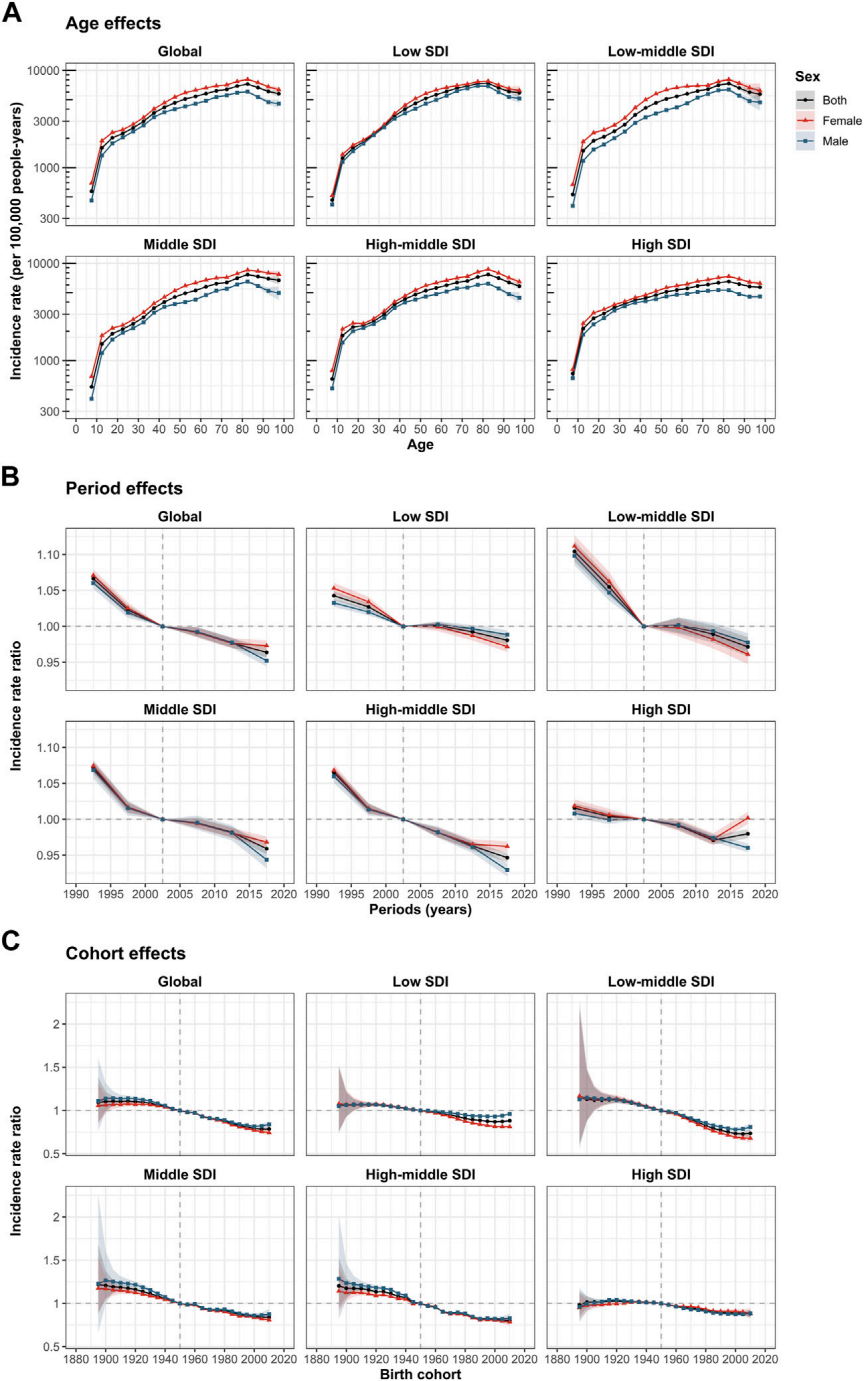
Age, period, and cohort effects on LBP incidence by SDI quintiles. **(A)** Age effects are shown by the fitted longitudinal age curves of incidence (per 100,000 population years), adjusted for period deviations. **(B)** Period effects are shown by the relative risk of incidence (incidence rate ratio) and computed as the ratio of age-specific rates from to 1990–1994 to 2015–2019, with the reference period set at 2000–2004. **(C)** Cohort effects are shown by the relative risk of incidence, computed as the ratio of age-specific rates from the 1895 cohort to the 2010 cohort, with the referent cohort set at 1950. The dots and shaded areas denote incidence rates or rate ratios and their corresponding 95% CIs, respectively. LBP, low back pain; SDI, socio-demographic index (Global Burden of Diseases Study, 21 Global Burden of Diseases regions, 1990–2019).

The estimates of age, period, and cohort effects by SDI quintile were derived from the APC model. Similar patterns of age effects were found across different SDI regions. The risk of incidence increased with age, peaking in the 80–84 years age group. The risk was the lowest in those aged 5–10 years, suggesting less severe symptoms. Compared with other regions, the high-middle SDI regions showed an overall higher incidence across all age groups. The incidence risk in women was higher than that in men in the overall and SDI quintiles (Figure 4A).

The period effects showed different degrees of incidence risk reduction across the different SDI quintiles. A more favorable relative risk reduction occurred in the low-middle SDI, middle SDI, and high-middle SDI regions. For countries with low SDI and middle SDI, the period effects remained almost constant during 2000–2014, indicating that there was little improvement in the incidence of LBP. Moreover, the period risk for women was lower than that for men during 2005–2019. In each SDI region, the gender difference in period risks between 2015 and 2019 was significantly greater than before. It is worth noting that, although the period risk of high SDI countries was low in the past decade, the relative risk of women showed an unfavorable and significant upward trend, inconsistent with the trends in other SDI regions (Figure 4B).

Globally, the overall risk of successively young-to-middle-aged birth cohorts has decreased. Like the period effects, the decline in cohort effects was more notable in low-middle SDI, middle SDI, and high-middle SDI countries. The incidence of people born after the 1960s in low-middle SDI countries improved significantly, while the relative risk in high SDI countries did not decrease until the 1950 cohort. Compared with individuals born in the 1950 reference cohort, the relative cohort risks of individuals born in the 2010 cohort ranged from 0.88 (95% CI 0.86–0.90) in low SDI countries to 0.74 (95% CI 0.69–0.78) in low-middle SDI countries (Figure 4C). The age, period, and cohort effects of LBP incidence in each country and territory are shown in Supplementary Figures S13–S27.

### Factors Associated With Net Drifts in Incidence

**FIGURE 5:**
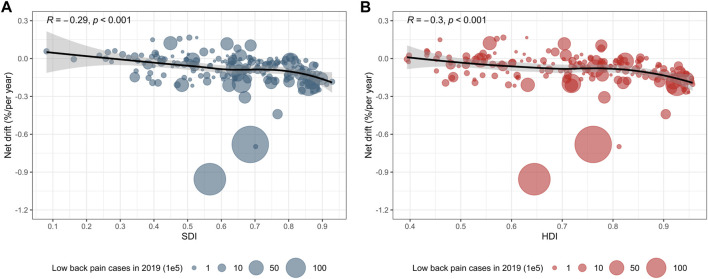
The correlation between net drifts in incidence and SDI as well as HDI. **(A)** The fitted correlation curve is represented by a black line, and the size of the blue bubbles represents the incident cases of LBP in 2019. The available SDI data can be matched with the incident cases in 204 countries and territories. **(B)** The fitted correlation curve is represented by a black line, and the size of the red bubbles represents the incident cases of LBP in 2019. The available HDI data can be matched with incident cases in 187 countries and territories. *p* < 0.001 indicated that the results were statistically significant. LBP, low back pain; SDI, socio-demographic index; HDI, Human Development Index (Global Burden of Diseases Study, 204 countries or territories, 2019).

A negative correlation (*R* = −0.29, ***p* < 0.001**) was observed between the net drifts in incidence and SDI (in 2019) for LBP (Figure 5A). A negative correlation (*R* = −0.3, ***p* < 0.001**) was found between the net drifts in incidence and HDI (in 2019) for LBP (Figure 5B).

### Predictions of Global Low Back Pain Incidence

**FIGURE 6:**
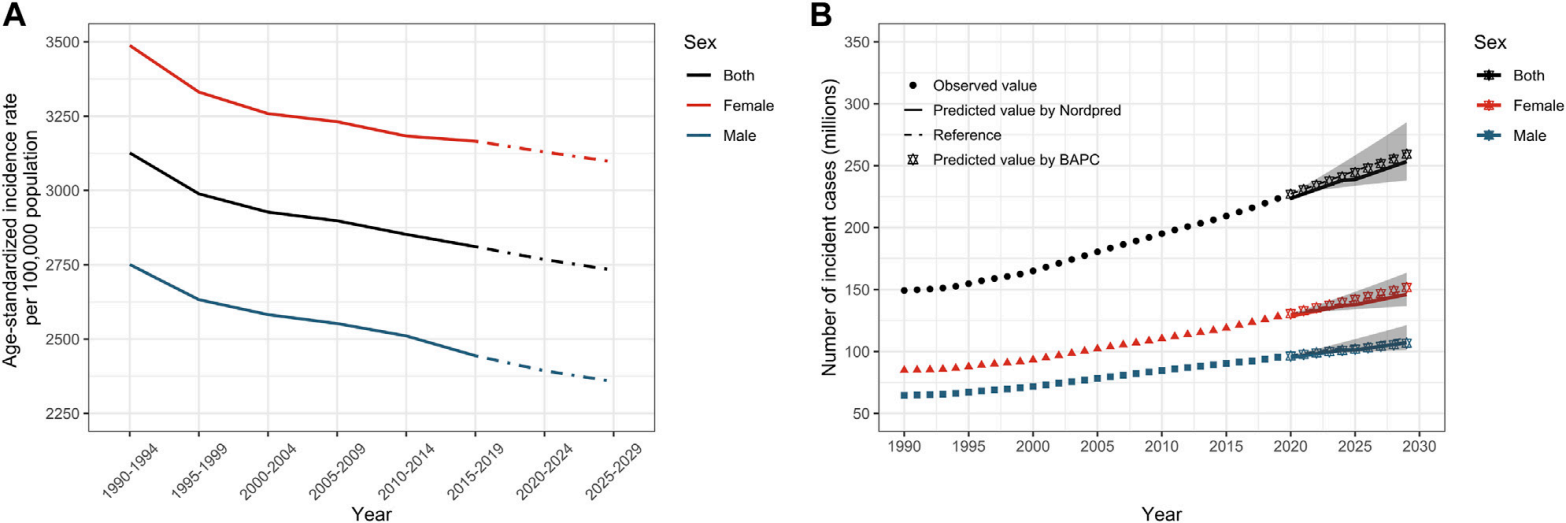
Global trends in incidence for LBP and its predicted level in the next decade. **(A)** Trends in the age-standardized incidence rate of LBP and its predicted level over the next decade. The solid lines represent the observed age-standardized incidence rates and dashed lines represent the predicted age-standardized incidence rates. **(B)** Trends in the number of incident cases of LBP and its predicted level over the next decade. The dots represent the observed values. The predicted values are represented by the solid lines (Nordpred) and stars (BAPC). The shaded areas indicate whether the rate remained stable (baseline reference), decreased by 1% per year (optimistic reference, lower limit), and increased by 1% per year (pessimistic reference, upper limit) based on the observed rate in 2019. LBP, low back pain; BAPC, Bayesian age period cohort (Global Burden of Diseases Study, 204 countries or territories, 1990–2019).

We predicted that the ASIR of LBP would continue to decrease slowly at the global level and that the decreasing trends would be stable in the future. The changing trends of the ASIR in females was similar to but significantly higher than that in males (Figure 6A).

Although the ASIRs for LBP have decreased in the past three decades and are expected to continue to decline in the future, the number of incident cases globally will continue to increase in the next decade because of population growth and aging. Globally, the number of new cases would increase from 223 million in 2019 to 253 million in 2029 for both sexes (males: 95–107 million; females: 128–146 million) (Figure 6B). To assess the robustness of our prediction results, we conducted a sensitivity analysis using the BAPC model to predict the future incidence of LBP worldwide. The results of the BAPC model showed consistent trends with the above results, indicating the stability of the prediction results (Figure 6B).

## Discussion

In the past 30 years, LBP has been the leading cause of the MSK disease burden globally and across all SDI regions. The proportion of LBP incident cases relative to all MSK cases decreased from 73.9% to 69.2%, accounting for more than two-thirds of all MSK cases. Globally, as the population increased by 44.6%, the number of incident cases increased by 49.67%, whereas the ASIR of LBP dropped by 13.25%. The counts of LBP incident cases increased across all SDI regions, and the percentage change was the largest in the low SDI region, up to 100.62%. Despite remarkable achievements in ASIRs in the past three decades, the change patterns suggest that LBP remains a major public health problem globally, since its disease burden has not substantially improved.

To the best of our knowledge, this is the first time that temporal trends in the incidence of LBP have been estimated based on an APC model at global, regional, and national levels, allowing horizontal comparison between SDI regions or countries, with some important findings presented. Compared with previous GBD 2019 publications [11–13], the main contribution of our research to this field is that the data were fully utilized to quantify the trends in the incidence of LBP, which provides more detailed insights for the public health sector. Because the assessment of incidence trends using traditional incidence indicators ignores important effects caused by differences in age, period, and birth cohort, it is not conducive to accurately assess incidence trends across all SDI regions. A prominent strength of this study is the estimation of local drifts in incidence based on period effects, which allowed us to track temporal trends in incidence for each age group. Equally important, we assessed the period and cohort effects of incidence trends, allowing us to identify the sources of changes in incidence trends by period and birth cohort. Our results facilitate an in-depth analysis of LBP incidence trends and provide a reference for healthcare services regarding the effectiveness of LBP treatment and prevention.

Between 1990 and 2019, large gaps remained in the changing trends of LBP incidence between countries, with faster rates of decline in low-middle SDI countries. Countries with an increasing trend of incidence were concentrated in regions with lower than moderate SDI. Especially in the low-SDI regions, the population increased by 56.2%, and the percentage increase relative to the global population was staggering, from 9.9% to 14.6%, and the number of LBP cases doubled. In contrast, the population of countries with high SDI and the total number of LBP cases increased by 23.3% and 31.1%, respectively. Most MSK disorders share common risk factors with other chronic diseases associated with aging. Population aging is expected to dramatically increase the economic and healthcare burden of LBP in the future.

Pakistan has a low SDI in the Eastern Mediterranean Region (EMR). Although the all-age incidence rate and ASIR were lower than the general level in the low SDI regions, the incidence rate deteriorated. The percentage changes in the all-age incidence rate and ASIR over the past three decades were 10.0% and 2.4%, respectively. We showed that its net drift was much higher than the overall level of the world and low SDI regions. Unfortunately, in low- or middle-income countries, there is a lack of evaluation or published data on the effectiveness of public health interventions for LBP. The incidence of LBP depends on the modeling of data from high-income countries, and the incidence rate may be underestimated. Public health strategies may be particularly important for low-income and middle-income countries [21]. Disease characteristics need to be understood to reduce the risk of LBP in many local populations, develop effective treatment schemes, and promote the concept of prevention.

China and India are middle and low-middle SDI countries, respectively. They belonged to the same category (significant decrease group) in the hierarchical cluster analysis based on trends in incidence. In the context of social and economic development, the largest declines in China and India may be due to improvements in health awareness [22]. Moreover, with economic development, the quality of life of the Chinese population has improved, and more attention has been paid to health. Young people prefer light physical labor [23–25]. Additionally, it is well known that, owing to the large population of India and China and with aging, the economic and social pressure faced is increasing, and the incidence of LBP among young people may be underestimated. The large drop in incidence does not indicate room for complacency, eliciting further investigation and data collection.

Most of the countries and regions with higher SDI had a downward trend in LBP incidence, while Canada and Saudi Arabia were two typical countries with an upward trend that was not commensurate with their socioeconomic development. Canada is a high-income country with a diverse population and culture. There are both indigenous and large immigrant populations. In the past 30 years, the population percentage has increased by 33.98%, and the all-age incidence rate has increased by 58.8%. The treatment recommendations present challenges for different populations. For example, if the therapist and patient do not speak the same language or misunderstand the various conceptual ways of LBP in different cultural groups, the implementation of cognitive behavioral therapy or mindfulness-based stress reduction therapy may be challenging [26]. Furthermore, a systematic and multidisciplinary care pathway was developed for low back pain to reduce variation in practice and improve quality and access to care [27–29]. Previous attempts have been made to change beliefs and behavior towards LBP through mass media campaigns, which have proven to be successful [30]. However, no significant downward trend has been observed over the past 30 years. Saudi Arabia is a rich country in EMR, in which the incidence rate was much higher than that of Pakistan, and it also showed an unfavorable trend. Previous studies have shown that the burden of diseases such as LBP, neck pain (NP), and osteoarthritis in EMR increased more than that in other parts of the world between 1990 and 2013 [31]. LBP and NP have the highest burden among musculoskeletal disorders in most EMR countries [31]. However, the relative importance of risk factors differed based on the income level of the countries [31]. This has resulted in different incidence rates in countries with different economic levels in the same region. In the prevention and control of any disease, public education, occupational health and safety, and ergonomic factors must be considered. It is essential to maintain various social functions to control the risk factors.

According to the analysis of age effects, the incidence of LBP was low in young adulthood and increased with age, peaking between 80 and 84 years of age. The observed association can be explained by changes induced by the aging process, such as postural problems, decreased flexibility, lack of regular physical exercise, and increased musculoskeletal degradation, which aggravate pain [32]. We showed that the incidence of LBP was greater in women than in men, corroborating data from Norway [33], Saudi Arabia [34], Thailand [35], and Israel [36]. This difference may result from the different proportions of muscle and adipose tissues in men and women.

We examined the correlation between incidence trends, SDI, and HDI. Net drifts in incidence were significantly negatively associated with both SDI and HDI, indicating a swifter decline in LBP burden in economically and socially developed regions. This phenomenon may be attributed to diverse social structures in different countries, including population, employment, and consumption structures. This may account for the observed burden of LBP relative to the expected burden based on the HDI and SDI when considering preventive measures.

Prevention programs targeting risk factors constitute a key approach. Although the mechanisms linking LBP and other chronic diseases are unclear, a previous longitudinal VISAT study identified occupational and ergonomic factors as pivotal in LBP incidence [37]. Highly demanding jobs, prolonged standing, and awkward lifting emerged as significant predictors of LBP [38]. Systematic reviews of cohort studies also suggest associations between lifestyle factors (smoking [39], obesity [40,41], and low physical activity [42]) and LBP episodes or persistent LBP, though independent relationships remain uncertain. Precise monitoring and attention to risk factors are crucial. Global, regional, and national prevention policies should promote healthy lifestyles, emphasizing exercise and balanced diets to combat overweight and obesity [9,43], ultimately enhancing quality of life. Health policymakers should integrate various risk factors to identify high-risk groups effectively.

This study presents several limitations. Firstly, our analysis inherits the limitations described in GBD 2019, such as limited or unavailable raw data in low- and middle-income countries, sparse data in some regions, and the impact of migration on the estimates. The estimates were derived from the GBD model in higher-resource environments. This leads to a wide range of uncertainties in GBD incidence rate estimates, which may affect age/period/cohort trends and exaggerate the improvement in some low SDI countries [19]. Secondly, the commonly used age interval data format in the APC model is the five-year age group, while the absence of incidence data for the 0–4 age group hinders the determination of effects on the youngest age group (≤4 years). Thirdly, due to the lack of data on individual socioeconomic status and various potential confounders, coupled with inadequate reporting systems in low HDI or SDI countries, caution is advised when interpreting the correlation between trends in incidence and SDI. Finally, despite estimating period and cohort effects, the APC model relies on GBD cross-sectional data and is not a cohort study. Comprehensive cohort studies in diverse countries are essential to ascertain relative risks at specific locations and times [19].

Globally, the burden of LBP remains substantial, with incident cases surpassing other common musculoskeletal disorders. Despite a downward trend in incidence rates across various SDI regions and some countries over the past 30 years, the incident cases and burden of LBP have risen worldwide due to an aging population. Our projections suggest a continued decline in global age-standardized rates over the next decade; however, incident cases are anticipated to substantially increase. A population-based, prospective cohort study showed that less than one-third of cases resolve annually, and more than 20% recur within 6 months [44]. Prevention and effective treatment of LBP require policy and health service interventions to mitigate the future disease burden.

### Conclusion

LBP remains a prominent global public health concern with varying temporal trends in incidence geographically. Despite a decrease in ASIRs over the past three decades, the burden persists at a high level. While ASIRs for LBP are anticipated to further decline over the next decade, the aging population will drive a significant increase in incident cases. Raising awareness about LBP and emphasizing disease prevention and control are crucial for mitigating future burdens. There is a pressing need for expanded data collection on individuals with LBP, especially in low-income countries, to facilitate health data refinement and effectively monitor the disease burden.

## Data Availability

The datasets generated and/or analysed during the current study are available in the GBD 2019 portal (http://ghdx.healthdata.org/gbd-2019).
