# Supplementary material for: Global, Regional, and National Change Patterns in the Incidence of Low Back Pain From 1990 to 2019 and Its Predicted Level in the Next Decade
Source: Int J Public Health. 2024 Feb 21;69:1606299. doi: 10.3389/ijph.2024.1606299 (PMC10915756; doi:10.3389/ijph.2024.1606299)
Supplement: Supplementary file 1 [file DataSheet1.PDF]

Supplementary appendix to

Global, regional, and national change patterns in the incidence of low back pain from 1990 to 2019 and its predicted level in the next decade

Methods

The age-period-cohort (APC) model

The APC model framework was used to analyze potential trends in incidence by age, period, and birth cohort[1]. The collinearity among age, period, and birth cohort effects (age = year-birth year) leads to difficulties in estimating their independent effects, the so-called identification problem[2]. The APC model aims to reveal the contribution of age-related biological factors, as well as technical and social factors, to disease trends beyond traditional epidemiological analyses[2]. We circumvented this problem by generating estimable APC parameters and functions without imposing arbitrary constraints on the model parameters[3].

Net drift and local drift are two important parameters in APC models. Net drift is an APC analog of the estimated annual percentage change (EAPC) in the age-standardized rate, representing the overall temporal trend in incidence. It is the log-linear component of the fitted temporal trends, period rate ratios, and cohort rate ratios[4]. Local drift is defined as EAPC over time, specific to age group[4]. The longitudinal age curve indicates the fitted longitudinal age-specific rates in the reference cohort adjusted for period deviations, reflecting age-related natural history (i.e., age effects)[4]. Period effects may be caused by changes in social, cultural, economic, or physical environments, represented by the relative risk of incidence for each period, tracking progress in different periods. Cohort effects can be related to changes between individuals or groups with the same birth year, reflected by cohort relative risks of incidence for each cohort, tracking incidence changes in different birth cohorts. Relative risk is computed as the ratio of age-specific rates in each period/cohort relative to the reference period/cohort[4].

The age and period interval entered must be perfectly consistent when analyzed in the APC model; therefore, the five-year age group should be used with the five-year calendar period. Because estimates in the GBD 2019 are presented by five-year age group with annual data, the LBP incidence estimates and population data for each country/territory were arranged into a consecutive five-year period from 1990 to 2019 (i.e., 1990-1994 [median, 1992], 1995-1999 [median, 1997]... 2015-2019 [median, 2017]). The samples consisted of 19 age groups (five-year consecutive age intervals from 5-9 to >95 years) and 24 consecutive cohorts, including cohorts born from to 1891-1899 (median, 1895) to 2006-2014 (median, 2010), with birth cohorts from 1946 to 1954 (median, 1950) as the reference group (Table S1). The APC model was implemented using the age-period-cohort web tool provided by the National Cancer Institute, as described in previous literature[4]. Wald χ 2-tests were employed to test the significance of the estimated parameters and functions. Statistical tests were two-sided, and statistical significance was set at $P < 0.05$.

References

1. Zou Z, Cini K, Dong B, et al. Time Trends in Cardiovascular Disease Mortality Across the BRICS. Circulation 2020; 141(10): 790-9.

2. Bell A. Age period cohort analysis: a review of what we should and shouldn't do. Ann Hum Biol 2020; 47(2): 208-17.
3. Rosenberg PS, Anderson WF. Age-period-cohort models in cancer surveillance research: ready for prime time? Cancer Epidemiol Biomarkers Prev 2011; 20(7): 1263-8.
4. Rosenberg PS, Check DP, Anderson WF. A web tool for age-period-cohort analysis of cancer incidence and mortality rates. Cancer Epidemiology, Biomarkers & Prevention : a Publication of the American Association For Cancer Research, Cosponsored by the American Society of Preventive Oncology 2014; 23(11): 2296-302.

Table S1. The lexis diagram of GBD data for the APC model

Period (median)	Age groups																				Birth cohort (median)
	0-4	5-9	10-14	15-19	20-24	25-29	30-34	35-39	40-44	45-49	50-54	55-59	60-64	65-69	70-74	75-79	80-84	85-89	90-94	95+	
																				X	1891-1899 (1895)
																			X	X	1896-1904 (1900)
																		X	X	X	1901-1909 (1905)
																	X	X	X	X	1906-1914 (1910)
																X	X	X	X	X	1911-1919 (1915)
															X	X	X	X	X	X	1916-1924 (1920)
														X	X	X	X	X	X		1921-1929 (1925)
													X	X	X	X	X	X			1926-1934 (1930)
												X	X	X	X	X	X				1931-1939 (1935)
											X	X	X	X	X	X					1936-1944 (1940)
										X	X	X	X	X	X						1941-1949 (1945)
									X	X	X	X	X	X							1946-1954 (1950)
								X	X	X	X	X	X								1951-1959 (1955)
							X	X	X	X	X	X									1956-1964 (1960)
						X	X	X	X	X	X										1961-1969 (1965)
					X	X	X	X	X	X											1966-1974 (1970)
				X	X	X	X	X	X												1971-1979 (1975)
			X	X	X	X	X	X													1976-1984 (1980)

		**X**	X	X	X	X	X														1981-1989 (1985)
	X	X	X	X	X	X															1986-1994 (1990)
1990-1994 (1992)	X	X	X	X	X																1991-1999 (1995)
1995-1999 (1997)	X	X	X	X																	1996-2004 (2000)
2000-2004 (2002)	X	X	X																		2001-2009 (2005)
2005-2009 (2007)	X	X																			2006-2014 (2010)
2010-2014 (2012)	X																				2011-2019 (2015)
2015-2019 (2017)																					

Note: X denotes mortality data of each age group from the corresponding period. For instance, the mortality rate of age 5-9 years in 1992 is filled in the square with a bold **X** (see table), and this square belongs to the cohort 1981-1989 (median, 1985).

Table S2. Time trends in low back pain incidence for both sexes in 204 countries and territories, 1990-2019

SDI quintile	Country	Population		Incident cases		All-age incidence		Age-standardized incidence		Net drift of incidence from APC model †, % per year
		Number in 2019	Percent change 1990-2019, %	Number in 2019	Percent change 1990-2019, %	Rate in 2019	Percent change 1990-2019, %	Rate in 2019	Percent change 1990-2019, %	
Low	Afghanistan	38277536.32 (26161825.87, 50468583.3)	235.21	781631.07 (677351.32, 895750.23)	199.89 (182.97, 217.25)	2042.01 (1769.58, 2340.15)	-10.54 (-15.59, -5.36)	3108.21 (2743.75, 3521.47)	0.58 (-2.44, 3.51)	0.0175 (-0.0126, 0.0476)
	Angola	30138521.38 (27054839.62, 33116640.83)	192.09	557122.86 (483950.15, 637857.06)	191.27 (183.16, 199.71)	1848.54 (1605.75, 2116.42)	-0.28 (-3.06, 2.61)	2863.51 (2525.77, 3251.39)	-0.84 (-3.47, 2.08)	-0.0448 (-0.0868, -0.0028)
	Bangladesh	159259849.53 (141199840.52, 177852817.87)	46.04	4307843.43 (3794696.42, 4886974.72)	94.52 (83.96, 104.94)	2704.91 (2382.71, 3068.55)	33.2 (25.96, 40.33)	2858.23 (2520.53, 3237.56)	-6.27 (-9.63, -2.9)	-0.198 (-0.2093, -0.1866)
	Benin	12665751.05 (11316886.46, 13983185.96)	161.01	238300.94 (207499.22, 272098.16)	172.08 (163.06, 180.5)	1881.46 (1638.27, 2148.3)	4.24 (0.78, 7.47)	2857.22 (2527.32, 3235.79)	-0.13 (-2.69, 2.63)	-0.0221 (-0.0637, 0.0196)
	Bhutan	754249.86 (696959.97, 815433.43)	23.21	20049.06 (17635.49, 22887.75)	67.9 (59.51, 76.06)	2658.15 (2338.15, 3034.	36.27 (29.46, 42.89)	2869.05 (2537.67, 3249.	-1.74 (-4.71, 0.99)	-0.0645 (-0.208, 0.079

						51)		31)		2)
	Burkina Faso	22691773.25 (19383320.45, 26173261.85)	137.42	428057.75 (373947.23, 489261.09)	145.78 (136.81, 155.4)	1886.4 (1647.94, 2156.12)	3.52 (-0.26, 7.57)	3809 (3384.79, 4299.12)	1.59 (-1.49, 4.8)	-0.0108 (-0.048, 0.0265)
	Burundi	11934360.75 (10304612.08, 13532060.78)	114.23	210592.45 (182913.12, 239769.18)	122.02 (114.62, 129.37)	1764.59 (1532.66, 2009.07)	3.64 (0.18, 7.07)	2589.67 (2291.21, 2934.25)	1.55 (-1.44, 4.78)	0.0652 (0.0149, 0.1155)
	Cambodia	16603117.7 (14206179.93, 18867659.67)	60.02	534408.2 (471611.54, 606369.28)	101.45 (92.62, 110.19)	3218.72 (2840.5, 3652.14)	25.88 (20.37, 31.35)	3596.19 (3197.66, 4072.36)	-5.66 (-8.21, -2.95)	-0.2092 (-0.2355, -0.1829)
	Central African Republic	5299863.15 (4459289.06, 6192556.53)	93.14	104867.2 (91196.69, 119989.88)	98.82 (91.95, 106.75)	1978.68 (1720.74, 2264.02)	2.94 (-0.62, 7.05)	2887.41 (2537.84, 3276.6)	-0.27 (-3.53, 2.64)	-0.0254 (-0.1044, 0.0536)
	Chad	16398859.74 (14327391.1, 18680096.03)	172.32	298243.09 (259648.97, 341691.01)	149.99 (141.13, 159.33)	1818.68 (1583.34, 2083.63)	-8.2 (-11.45, -4.77)	2990.69 (2651.9, 3379.57)	-0.35 (-3.47, 2.87)	0.0368 (-0.0014, 0.0751)
	Comoros	714351.07 (593290.69, 837233.34)	53.38	17135.24 (15039.17, 19537.02)	100.15 (91.88, 107.87)	2398.71 (2105.29, 2734.93)	30.49 (25.1, 35.53)	2814.26 (2491.27, 3196.6)	0.72 (-2.58, 3.97)	0.0158 (-0.1291, 0.1609)
	Côte d'Ivoire	26171531.6 (23573125.28, 28872979)	114.06	531447.89 (462721.08, 612080.67)	145.78 (136.91, 154.74)	2030.63 (1768.03, 2338.73)	14.82 (10.68, 19.01)	2851.83 (2524.29, 3230.87)	1.35 (-1.57, 4.34)	0.0055 (-0.0339, 0.0449)

	Democratic Republic of the Congo	87670443.78 (61748630.02, 112590350.36)	127.18	1694839.6 (1478578.5, 1942679.48)	137.26 (129.87, 144.72)	1933.19 (1686.52, 2215.89)	4.44 (1.18, 7.72)	2855.1 (2530.18, 3235.81)	-1.09 (-3.99, 1.72)	-0.0434 (-0.0655, -0.0212)
	Djibouti	1202796.73 (1050469.44, 1362651.13)	147.48	24843.93 (21607.32, 28624.64)	224.79 (208.65, 242.63)	2065.51 (1796.42, 2379.84)	31.23 (24.71, 38.45)	2626.97 (2321.58, 2995.76)	-1.1 (-4.42, 2)	-0.0278 (-0.2464, 0.1914)
	Eritrea	6711213.33 (4780647.5, 8595692.74)	123.6	115482.04 (99599.81, 132866.17)	167.06 (157.99, 176.44)	1720.73 (1484.08, 1979.76)	19.44 (15.38, 23.63)	2504.73 (2200.19, 2847.65)	3.5 (0.28, 6.56)	0.1527 (0.0511, 0.2545)
	Ethiopia	107591163.68 (92024285.35, 122776186.49)	109.36	1845491.24 (1606008.54, 2112834.13)	118.11 (113.85, 122.51)	1715.28 (1492.7, 1963.76)	4.18 (2.15, 6.28)	2673.52 (2364.07, 3043.93)	-2.27 (-3.67, -0.74)	-0.1571 (-0.1788, -0.1354)
	Gambia	2245865.71 (2027999.72, 2476236.13)	126.41	43157.34 (37480.82, 49239.91)	151.61 (144.59, 159.77)	1921.63 (1668.88, 2192.47)	11.13 (8.03, 14.74)	2747.75 (2420.71, 3108.53)	-0.36 (-2.96, 2.4)	-0.0643 (-0.191, 0.0625)
	Guinea	12643148.93 (11365936, 13951889.49)	104.37	243173.61 (212650.61, 276982.69)	98.07 (91.96, 104.95)	1923.36 (1681.94, 2190.77)	-3.08 (-6.07, 0.28)	2836.99 (2504.29, 3234.61)	0.76 (-1.93, 3.45)	0.0044 (-0.0313, 0.0401)
	Guinea-Bissau	1901191.44 (1666338.83, 2146163.8)	88.74	36003.5 (31290.47, 41215.27)	98.5 (91.69, 106.28)	1893.73 (1645.83, 2167.87)	5.17 (1.56, 9.29)	2774 (2446.7, 3148.39)	-0.25 (-3.27, 2.7)	-0.0212 (-0.1515, 0.1093)

	Haiti	12402098.71 (10373558.08, 14713362.41)	95.13	289471.15 (252895.52, 331111.39)	117.28 (109.58, 125.47)	2334.05 (2039.13, 2669.8)	11.35 (7.4, 15.55)	2770.61 (2438.44, 3145.15)	-0.42 (-3.42, 2.87)	-0.0047 (-0.0502, 0.0409)
	Liberia	4789906.94 (4131045.49, 5420863.09)	143.86	97809.34 (84893.63, 112463.23)	144.87 (134.48, 155.31)	2041.99 (1772.34, 2347.92)	0.41 (-3.84, 4.7)	2776.2 (2445.51, 3134.69)	-0.44 (-3.23, 2.46)	-0.0391 (-0.1073, 0.0291)
	Madagascar	26690344 (20373797.57, 32844323.86)	123.32	500686.3 (434821.02, 575211.55)	127.41 (119.88, 136.27)	1875.91 (1629.13, 2155.13)	1.83 (-1.54, 5.8)	2741.2 (2411.69, 3135.79)	-4.83 (-7.57, -1.63)	-0.1893 (-0.2202, -0.1583)
	Malawi	18442238.48 (17149774.44, 19745166.65)	93	323991.89 (281374.84, 371848.14)	93.92 (87.1, 100.86)	1756.79 (1525.71, 2016.29)	0.48 (-3.06, 4.07)	2691.2 (2364.28, 3061.3)	-3.5 (-6.61, -0.39)	-0.1893 (-0.2202, -0.1583)
	Mali	21917466.99 (19126100.67, 24868427.76)	152.72	364273.6 (318756.94, 417070.39)	152.84 (143.2, 163.18)	1662.02 (1454.35, 1902.91)	0.05 (-3.76, 4.14)	2830.44 (2496.74, 3187.16)	4.72 (1.56, 8.36)	0.0251 (-0.0131, 0.0633)
	Mozambique	29528036.87 (27057906.86, 31808337.18)	125.9	514693.2 (449901.18, 589427.55)	112.49 (105.14, 120.2)	1743.07 (1523.64, 1996.16)	-5.94 (-9.19, -2.53)	2816.37 (2488.76, 3195.4)	0.58 (-2.42, 3.44)	0.034 (0.0026, 0.0654)
	Nepal	30416382.4 (26611408.34, 34238807.5)	55.69	846613.66 (742645.66, 960559.68)	93.31 (85.53, 101.4)	2783.41 (2441.6, 3158.03)	24.16 (19.17, 29.36)	3135.17 (2767.91, 3544.36)	-1.51 (-4.88, 1.81)	-0.0202 (-0.0431, 0.0028)

	Niger	23295352.88 (20797659.81, 25931630.96)	190.36	388795.79 (337514.42, 445199.34)	183.63 (174.43, 194.22)	1668.98 (1448.85, 1911.11)	-2.32 (-5.49, 1.33)	2808.11 (2483.53, 3186.56)	0.44 (-2.55, 3.7)	-0.0052 (-0.0531, 0.0426)
	Pakistan	224062846.78 (207077327.29, 241657356.15)	98.57	4127306.15 (3539079.99, 4763854.62)	118.46 (113.31, 123.22)	1842.03 (1579.5, 2126.12)	10.02 (7.42, 12.41)	2535.35 (2208.55, 2904.11)	2.41 (0.71, 4.07)	0.1202 (0.1054, 0.1349)
	Papua New Guinea	9866614.4 (8688195.02, 10952387.86)	141.39	289570.65 (252866.62, 332062.91)	158.74 (149.77, 167.5)	2934.85 (2562.85, 3365.52)	7.19 (3.47, 10.82)	3837.33 (3407.21, 4338.17)	0.2 (-2.63, 3.01)	0.0168 (-0.0307, 0.0643)
	Rwanda	12688117.31 (11344257.63, 14076210.33)	76.89	261999.5 (229206.81, 300032.2)	101.43 (93.5, 109.64)	2064.92 (1806.47, 2364.67)	13.87 (9.39, 18.51)	2767.87 (2451.35, 3138.9)	-3 (-6.04, 0.47)	-0.1308 (-0.176, -0.0855)
	Senegal	15134067.15 (13503991.83, 16852508.63)	98.59	291135.7 (255570.35, 331979.86)	123.68 (115.92, 131.77)	1923.71 (1688.71, 2193.59)	12.63 (8.73, 16.7)	2622.3 (2309.71, 2967.96)	-0.6 (-3.52, 2.59)	-0.132 (-0.1695, -0.0944)
	Sierra Leone	8284755.48 (7526347.64, 9080217.52)	126.85	164606.29 (143458.85, 187688.61)	118.42 (110.64, 126.48)	1986.86 (1731.6, 2265.47)	-3.72 (-7.14, -0.16)	2801.96 (2484.55, 3174.98)	-2.85 (-5.65, 0.13)	-0.0982 (-0.1452, -0.0511)
	Solomon Islands	655631.94 (570723.96, 742583.38)	92.58	18667.41 (16308.27, 21396.24)	122.46 (113.57, 131.52)	2847.24 (2487.41, 3263.45)	15.51 (10.9, 20.22)	3820.15 (3391.36, 4319.07)	1.31 (-1.56, 4.42)	0.0429 (-0.2305, 0.317)

	Somalia	20343111.95 (15201444.92, 25703627.38)	184.62	340493.62 (296039.16, 392492.42)	181.85 (171.12, 192.72)	1673.75 (1455.23, 1929.36)	-0.97 (-4.74, 2.85)	2778.73 (2444.31, 3153.48)	1.27 (-1.61, 4.44)	0.0587 (0.0069, 0.1104)
	South Sudan	9282963.05 (8050826.26, 10613472.26)	58.43	170615.56 (149218.33, 196090.23)	65.07 (58.44, 71.33)	1837.94 (1607.44, 2112.37)	4.19 (0.01, 8.15)	2789.19 (2453.97, 3166.66)	0.59 (-2.64, 3.49)	0.0237 (-0.0201, 0.0674)
	Togo	7921526.59 (6943022.31, 8897861.83)	116.26	163526.15 (142304.85, 187329.43)	160.07 (149.99, 171.07)	2064.33 (1796.43, 2364.81)	20.26 (15.6, 25.35)	2783.88 (2463.17, 3154.34)	0.18 (-2.88, 3.46)	-0.018 (-0.0802, 0.0442)
	Uganda	41117856.24 (37023695.6, 44955510.63)	137.46	686019.66 (593468.14, 781919.69)	144.04 (136.13, 152.21)	1668.42 (1443.33, 1901.65)	2.77 (-0.56, 6.21)	2757.46 (2429.48, 3134.47)	0.12 (-2.75, 3.24)	0.0438 (0.0158, 0.0719)
	United Republic of Tanzania	56736116.17 (50495484.71, 63230442.97)	119.05	1018492.5 (886211.57, 1161188.75)	129.75 (121.69, 137.53)	1795.14 (1561.99, 2046.65)	4.89 (1.21, 8.44)	2686.51 (2373.79, 3045.64)	-1.66 (-4.54, 1.26)	-0.0081 (-0.0309, 0.0148)
	Vanuatu	294550.46 (267953.02, 320614)	94.57	9778.2 (8657.42, 11045.66)	129.63 (122.27, 137.35)	3319.7 (2939.2, 3750)	18.02 (14.24, 21.99)	2790.88 (2462.78, 3165.08)	2 (-0.88, 4.95)	0.0512 (-0.2159, 0.319)
	Yemen	31502896.33 (26596895.42, 36775107.04)	129.46	707217.77 (614181.32, 811160.95)	163.97 (154.11, 174.25)	2244.93 (1949.6, 2574.88)	15.04 (10.74, 19.52)	3072.84 (2702.73, 3471.55)	-4.4 (-7.25, -1.15)	-0.1932 (-0.2256, -0.1609)

	Zimbabwe	15010852.06 (13317204.38, 16650708.61)	45.2	298105.75 (259973.09, 341998.14)	73.89 (67.74, 80.52)	1985.93 (1731.9, 2278.34)	19.76 (15.52, 24.33)	2795.34 (2462.96, 3176.93)	4 (0.84, 7.32)	0.1544 (0.1189, 0.1899)
**Low-middle**	Belize	410093.52 (358839.75, 459147.99)	120.62	10350.21 (9050.05, 11818.54)	173.48 (160.48, 186.31)	2523.87 (2206.82, 2881.91)	23.96 (18.07, 29.78)	2769.13 (2442.15, 3145.13)	-0.73 (-3.58, 2.37)	-0.0236 (-0.1957, 0.1488)
	Bolivia (Plurinational State of)	12011749.54 (10641659.36, 13418191.33)	87.07	287382.46 (252523.76, 326926.66)	121.2 (112.44, 130.42)	2392.51 (2102.31, 2721.72)	18.25 (13.57, 23.18)	2677.54 (2355.04, 3028.44)	-1.63 (-4.5, 0.98)	-0.0593 (-0.0972, -0.0214)
	Cabo Verde	563563.09 (494550.4, 632144.21)	60.28	14396.31 (12672.65, 16432.39)	100.45 (89.85, 112.37)	2554.52 (2248.67, 2915.8)	25.07 (18.45, 32.5)	2776.92 (2447.85, 3149.3)	-0.35 (-3.21, 2.65)	-0.0404 (-0.1528, 0.0721)
	Cameroon	29101868.38 (24782960.15, 33604810.26)	180.07	604298.31 (526117.79, 688318.73)	192.85 (183.29, 202.29)	2076.49 (1807.85, 2365.2)	4.56 (1.15, 7.93)	2946.96 (2593.39, 3341.13)	-0.86 (-3.6, 2.05)	-0.0474 (-0.0788, -0.016)
	Congo	5265846.28 (4507068.1, 6008799.34)	115.39	114429.89 (100180.6, 131313.95)	148.79 (137.31, 160.36)	2173.06 (1902.46, 2493.69)	15.51 (10.18, 20.88)	2822.37 (2498.69, 3193.11)	-0.53 (-3.68, 2.72)	-0.0583 (-0.1582, 0.0418)
	Democratic People's Republic of Korea	26232860.94 (22628874.75, 29910476.56)	24.59	988042.54 (872824.64, 1123533.28)	52 (45.83, 58.26)	3766.43 (3327.22, 4282.92)	22 (17.04, 27.02)	3256.1 (2889.47, 3675.82)	-5.64 (-8.1, -2.82)	-0.178 (-0.1924, -0.1636)

	Dominican Republic	10881854.5 (9629750.84, 12279752.92)	51.07	281672.18 (247704.66, 320192.13)	91.17 (81.94, 100.42)	2588.46 (2276.31, 2942.44)	26.54 (20.43, 32.66)	2675.93 (2355.95, 3031.76)	2.07 (-1.39, 5.22)	-0.0217 (-0.0537, 0.0103)
	El Salvador	6256142.86 (5393103.7, 7102866.92)	18.77	182160.4 (160718, 206783.42)	50.01 (43.66, 56.97)	2911.7 (2568.96, 3305.29)	26.3 (20.96, 32.16)	2825.48 (2497.11, 3202.72)	1.01 (-2.05, 4)	0.0647 (0.0337, 0.0958)
	Eswatini	1142108.63 (1051093.13, 1230315.19)	41.58	20910.63 (18261.94, 23918.53)	73.51 (66.4, 80.33)	1830.88 (1598.97, 2094.24)	22.56 (17.53, 27.37)	2450.68 (2154.71, 2778.39)	-2 (-5.21, 1.12)	-0.1638 (-0.2986, -0.0289)
	Ghana	31536231.95 (27445271.15, 35185758.19)	110.01	600477.53 (528671.68, 679452.12)	139.97 (131.51, 148.76)	1904.09 (1676.39, 2154.51)	14.26 (10.24, 18.45)	2448.1 (2178.8, 2740.07)	-1.89 (-5.48, 1.59)	-0.1531 (-0.1833, -0.1229)
	Guatemala	17776489.98 (14652056.68, 20925960.32)	123.14	466100.31 (406346.04, 531405.61)	167.9 (157.55, 178.45)	2622 (2285.86, 2989.37)	20.06 (15.42, 24.79)	3008.93 (2659.05, 3388.5)	-0.25 (-3.68, 3.1)	-0.0183 (-0.0626, 0.0261)
	Guyana	770705.22 (683791.64, 857060.35)	0.08	20468.15 (17943.6, 23409.43)	21.29 (15.52, 27.3)	2655.77 (2328.21, 3037.4)	21.19 (15.42, 27.2)	2748.71 (2426.18, 3120.79)	-1.19 (-3.98, 1.92)	-0.0402 (-0.1583, 0.078)
	Honduras	9814395.7 (8823026.78, 10833410.07)	108.44	251564.11 (219329.67, 288022.62)	155.1 (145.62, 164.91)	2563.22 (2234.78, 2934.7)	22.39 (17.84, 27.1)	2955.28 (2595.97, 3353.76)	-0.03 (-2.82, 3.12)	-0.008 (-0.0488, 0.0327)

	India	1390706967.59 (1237773389.51, 1558771721.08)	62.54	29625621.09 (26011120.94, 33743475.91)	52.02 (46.56, 57.4)	2130.26 (1870.35, 2426.35)	-6.47 (-9.83, -3.16)	2268.6 (1998.28, 2574.65)	-24.04 (-25.57, -22.49)	-0.9694 (-1.2402, -0.6978)
	Kenya	50227709.45 (43651057.76, 56751223.08)	116.56	998193.48 (869463.63, 1140469.18)	160.8 (155.74, 165.66)	1987.34 (1731.04, 2270.6)	20.43 (18.09, 22.67)	2812.67 (2491.6, 3190.83)	-1.14 (-1.76, -0.59)	-0.034 (-0.0564, -0.0115)
	Kiribati	118620.94 (107352.04, 128869.07)	60.16	3825.58 (3366.43, 4354.88)	82.92 (77.09, 88.93)	3225.04 (2837.98, 3671.26)	14.21 (10.57, 17.97)	3968.44 (3538.44, 4467.66)	3.29 (0.58, 6.3)	0.1711 (-0.2618, 0.6058)
	Kyrgyzstan	6535458.86 (5697804.65, 7315212.48)	46.46	180485.26 (158452.34, 204980.91)	56.9 (51, 63.16)	2761.63 (2424.5, 3136.44)	7.13 (3.1, 11.4)	3123.88 (2754.44, 3527.1)	-2.09 (-5.31, 0.82)	-0.0917 (-0.1235, -0.0598)
	Lao People's Democratic Republic	7158249.73 (6469590.48, 7826129.87)	72.42	211094.42 (186033.51, 240583.94)	104.33 (95.76, 112.02)	2948.97 (2598.87, 3360.93)	18.51 (13.54, 22.97)	3465.52 (3082.69, 3909.46)	-1.51 (-4.85, 1.42)	-0.1752 (-0.2201, -0.1303)
	Lesotho	2091587.94 (1910348.4, 2273274.29)	15.73	42557.97 (37094.7, 48552.18)	28.42 (23.83, 33.18)	2034.72 (1773.52, 2321.31)	10.96 (7, 15.07)	2485.97 (2198.07, 2830.21)	-3.34 (-6.2, -0.58)	-0.1655 (-0.238, -0.093)
	Maldives	498414.07 (448540.34, 546426.1)	124.59	15064.93 (13108.8, 17453.17)	222.46 (199.97, 243.75)	3022.57 (2630.1, 3501.74)	43.58 (33.57, 53.06)	3168.74 (2816.18, 3572.25)	-2.82 (-5.64, 0.04)	-0.1971 (-0.2139, -0.1803)

	Marshall Islands	56842.25 (49772.32, 63374.4)	24.3	1815.65 (1592.8, 2070.01)	65.23 (56.4, 74.43)	3194.19 (2802.13, 3641.68)	32.92 (25.82, 40.33)	3735.85 (3324.55, 4210.86)	-2.34 (-5.17, 0.65)	-0.085 (-0.6624, 0.4958)
	Mauritania	4014273.45 (3561886.66, 4441999.29)	94.26	83849.08 (73218.09, 95946.18)	112.34 (105.65, 120.24)	2088.77 (1823.94, 2390.13)	9.3 (5.86, 13.37)	2934.4 (2577.82, 3323.78)	0.42 (-2.49, 3.54)	-0.0057 (-0.0759, 0.0646)
	Micronesia (Federated States of)	102115.6 (89818.87, 114353.73)	-2.08	3450.51 (3035.4, 3919.16)	26.64 (20.48, 33.37)	3379.02 (2972.52, 3837.97)	29.33 (23.03, 36.2)	3816.46 (3394, 4289.41)	-0.48 (-3.44, 2.47)	-0.0197 (-0.3635, 0.3253)
	Mongolia	3387589.16 (2977540.68, 3795389.42)	57.29	97809.21 (85044.28, 112493.37)	97.72 (84.56, 108.84)	2887.28 (2510.47, 3320.75)	25.7 (17.34, 32.77)	3121.94 (2755.48, 3536.23)	-3.61 (-6.29, -0.8)	-0.1592 (-0.2315, -0.0868)
	Morocco	35952185.54 (32338676.28, 39427377.46)	42.12	1165370.59 (1027187.54, 1327665.99)	86.23 (77.87, 94.99)	3241.45 (2857.09, 3692.87)	31.03 (25.15, 37.2)	3230.53 (2860.06, 3658.14)	1.29 (-1.54, 4.26)	0.0522 (0.0352, 0.0691)
	Myanmar	54676900.9 (48907817.94, 60236348.5)	33.02	1689059.61 (1492550.68, 1925546.3)	72.11 (64, 80.83)	3089.16 (2729.76, 3521.68)	29.39 (23.29, 35.94)	3197.77 (2844.61, 3623.96)	2.31 (-1.28, 6.1)	-0.097 (-0.1097, -0.0844)
	Namibia	2403123.93 (2112720.06, 2685060.49)	70.47	50189.69 (44008.87, 57287.86)	94.82 (86.4, 102.68)	2088.52 (1831.32, 2383.89)	14.28 (9.34, 18.89)	2640.98 (2338.57, 2997.09)	-0.19 (-3.56, 3.13)	-0.0296 (-0.1132, 0.054)
	Nauru	10551.3 (9292.48,	2.89	303.16 (264.7,	13.36 (9.78,	2873.19	10.17 (6.7,	3872.88	0.56 (-2.02,	0.0216

		11846.38)		348.28)	16.97)	(2508.65, 3300.82)	13.69)	(3454.25, 4378.97)	3.2)	(-1.5022, 1.569)
	Nicaragua	6510364.64 (5514375.95, 7574279.76)	67.49	174299.84 (152913.13, 198912.13)	114 (103.41, 125.29)	2677.27 (2348.76, 3055.31)	27.77 (21.45, 34.52)	2924.34 (2574.94, 3324.01)	-3.01 (-5.97, -0.13)	-0.0961 (-0.1399, -0.0523)
	Nigeria	214823785.71 (193132521.48, 236573636.29)	138.2	4814246.54 (4198936.32, 5506041.41)	135.9 (131.9, 139.88)	2241.02 (1954.6, 2563.05)	-0.97 (-2.64, 0.71)	3238.47 (2865.42, 3654.51)	0.8 (0.09, 1.47)	-0.0169 (-0.0427, 0.0089)
	Palestine	4956596.67 (4561834.93, 5329689.26)	139.45	117258.78 (102575.95, 134387.73)	182.46 (170.75, 193.62)	2365.71 (2069.48, 2711.29)	17.96 (13.07, 22.62)	3042.86 (2687.46, 3462.53)	-2.7 (-5.6, 0.69)	-0.098 (-0.1587, -0.0373)
	Philippines	112142764.27 (101580879.79, 121868834.7)	77.19	3729919.24 (3296122.33, 4242003.57)	99.84 (94.14, 105.37)	3326.05 (2939.22, 3782.68)	12.78 (9.57, 15.9)	3789.79 (3368.07, 4276.91)	-5.72 (-6.39, -5.07)	-0.2167 (-0.2382, -0.1951)
	Sao Tome and Principe	205385.14 (182423.64, 229091.42)	69	4161.38 (3622.22, 4771.59)	93.78 (85.41, 102.77)	2026.13 (1763.62, 2323.24)	14.67 (9.71, 19.99)	2586.86 (2294.31, 2935.09)	1.31 (-1.31, 4.23)	0.0322 (-0.2429, 0.308)
	Sudan	40808424.84 (35356108.64, 46009715.24)	102.03	927367.57 (803297.79, 1067814.5)	118.74 (111.68, 125.36)	2272.49 (1968.46, 2616.65)	8.27 (4.77, 11.55)	3010.11 (2654, 3429.23)	-1.21 (-3.96, 1.36)	-0.0482 (-0.071, -0.0254)
	Syrian Arab Republic	14491246.78 (12173712.54,	12.38	433622.64 (378476.57,	61.75 (51.81, 72.29)	2992.31 (2611.76,	43.93 (35.09, 53.31)	3056.33 (2686.23,	-3.59 (-6.28, -0.84)	-0.1405 (-0.1676,

		16795620.34)		495509.22)		3419.37)		3463.24)		-0.1133)
	Tajikistan	9492413.69 (8213913.13, 10674835.23)	76.58	234981.02 (205457.08, 269165.95)	96.19 (87.94, 105.23)	2475.46 (2164.43, 2835.59)	11.1 (6.43, 16.23)	3053.38 (2691.51, 3476.04)	-3.42 (-5.94, -0.58)	-0.1349 (-0.1674, -0.1023)
	Timor-Leste	1334823.34 (1208832.25, 1447383.06)	70.48	34002.64 (29951.89, 38633.82)	86.92 (78.65, 96.11)	2547.35 (2243.88, 2894.3)	9.64 (4.79, 15.03)	3266.06 (2887.48, 3694.64)	-5.21 (-8.01, -2.6)	-0.2185 (-0.32, -0.1169)
	Tuvalu	11797.83 (10461.54, 13230.45)	26.24	432.53 (382.96, 486.33)	37.97 (33.85, 42.1)	3666.2 (3246.06, 4122.22)	9.3 (6.03, 12.57)	3856.56 (3428.86, 4339.88)	-2.13 (-4.96, 0.8)	-0.0865 (-0.888, 0.7215)
	Venezuela (Bolivarian Republic of)	28068985.78 (24769383.18, 31423834.36)	49.07	833793.77 (732586.94, 944501.06)	87.19 (76.82, 98.61)	2970.52 (2609.95, 3364.93)	25.58 (18.62, 33.23)	2820.33 (2482.42, 3184.67)	-3.53 (-6.56, -0.34)	-0.1305 (-0.1484, -0.1127)
	Viet Nam	96372928.24 (83066413.94, 109007011.93)	41.85	3551710.95 (3129780.75, 4059097.35)	105.83 (94.34, 117.46)	3685.38 (3247.57, 4211.86)	45.11 (37.01, 53.31)	3471.23 (3075.36, 3922.07)	4.14 (0.93, 7.8)	0.1057 (0.098, 0.1133)
	Zambia	18237682.67 (15885855.21, 20473278.41)	129.61	312275.55 (270696.48, 360185.68)	173.28 (162.83, 184.12)	1712.25 (1484.27, 1974.95)	19.02 (14.47, 23.74)	2645.7 (2328.65, 3014.19)	8.11 (4.61, 11.63)	0.1551 (0.1064, 0.2037)
**Middle**	Albania	2720353.06 (2418307.85, 3021833.6	-17.81	134797.04 (119403.09, 153322.2	14.05 (6.51, 21.68)	4955.13 (4389.25, 5636.	38.77 (29.59, 48.05)	4099.45 (3626.32, 4642.	-1.67 (-4.81, 1.26)	-0.0572 (-0.0926, -0.021

		6)		5)		12)		5)		9)
	Algeria	41847289.68 (36020517.82, 47461911.69)	65.52	1257427.26 (1097528.94, 1445307.66)	119.79 (108.14, 131.51)	3004.8 (2622.7, 3453.77)	32.79 (25.75, 39.87)	3081.24 (2713.31, 3497.96)	-1.85 (-4.7, 1.07)	-0.0819 (-0.1238, -0.04)
	Armenia	3019674.3 (2651852.4, 3385871.26)	-11.57	120087.38 (106099.43, 135986.33)	14.11 (8.65, 19.55)	3976.83 (3513.61, 4503.34)	29.03 (22.86, 35.18)	3348.04 (2968.72, 3786.6)	-0.7 (-3.75, 2.51)	-0.0093 (-0.0474, 0.0289)
	Azerbaijan	10278673.96 (8953531.95, 11640071.99)	40.21	341777.56 (298651.36, 392073.7)	75.47 (67.21, 83.9)	3325.11 (2905.54, 3814.44)	25.15 (19.26, 31.16)	3160.86 (2792.18, 3581.32)	0.01 (-2.77, 3.22)	0.01 (-0.0162, 0.0362)
	Botswana	2338721.3 (2084722.17, 2607764.27)	79.67	50105.55 (43880.75, 57626.07)	126.35 (116.47, 137.23)	2142.43 (1876.27, 2464)	25.98 (20.48, 32.04)	2539.45 (2243.21, 2882.84)	-1.71 (-4.4, 1.27)	-0.03 (-0.1309, 0.0709)
	Brazil	216664813.68 (189879231.99, 242502434.01)	45.57	7631690.28 (6709900.98, 8648467.77)	85.68 (78.31, 93.38)	3522.35 (3096.9, 3991.63)	27.55 (22.49, 32.84)	3242.47 (2859.43, 3665.58)	0.42 (-0.57, 1.39)	-0.0986 (-0.1213, -0.0758)
	China	1422350422.36 (1239302360.26, 1597063538.03)	20.16	40158356.95 (35369447.69, 45809146.32)	20.85 (14.15, 26.93)	2823.38 (2486.69, 3220.67)	0.57 (-5, 5.63)	2280.67 (2017.18, 2572.62)	-28.15 (-29.74, -26.59)	-0.6826 (-0.7624, -0.6028)
	Colombia	47776679.01 (44174337.19, 51539500.	46.79	1630741.91 (1442304.04, 1848789.	89.87 (79.66, 100.93)	3413.26 (3018.85, 3869.	29.35 (22.39, 36.88)	3192.39 (2827.64, 3616.	0.67 (-2.82, 4.45)	-0.0112 (-0.0285, 0.006)

		94)		92)		65)		83)		
	Costa Rica	4716743.81 (4164759.49, 5271507.95)	55.16	146400.82 (129161.89, 166424.46)	97.26 (86.37, 108.95)	3103.85 (2738.37, 3528.38)	27.13 (20.12, 34.66)	2872.69 (2533.41, 3257.43)	-2.62 (-5.78, 0.22)	-0.0929 (-0.1325, -0.0533)
	Cuba	11358510.25 (10094746.43, 12738845.06)	4.86	410683.37 (368610.95, 457067.76)	28.42 (19.89, 37.32)	3615.64 (3245.24, 4024.01)	22.47 (14.34, 30.96)	2818.43 (2521.05, 3136.02)	-3.35 (-7.83, 1.8)	-0.085 (-0.1047, -0.0653)
	Ecuador	17588392.32 (15403893.92, 19749862.62)	75.42	417010.53 (373427.49, 463971.9)	107.79 (96.91, 119.46)	2370.94 (2123.15, 2637.94)	18.45 (12.25, 25.1)	2470.52 (2218.62, 2744.1)	-3.91 (-8.1, 0.89)	-0.1694 (-0.1984, -0.1404)
	Egypt	99069551.17 (90571861.47, 107515771.48)	77.87	2738367.95 (2402635.87, 3135984.39)	106.77 (99.52, 114.42)	2764.09 (2425.2, 3165.44)	16.25 (12.17, 20.55)	3155.71 (2789.93, 3568.52)	1.31 (-1.62, 4.29)	0.0247 (0.0095, 0.0399)
	Equatorial Guinea	1419839.1 (1290597.79, 1552892.25)	229.93	26523.06 (22845.48, 30350.91)	227.37 (212.53, 243.83)	1868.03 (1609.02, 2137.63)	-0.78 (-5.28, 4.21)	2808.71 (2476.48, 3167.06)	-2.51 (-5.53, 0.53)	-0.1091 (-0.2787, 0.0607)
	Fiji	911248.46 (838785.91, 984189.1)	20.01	32973.27 (29022.4, 37656.33)	46.44 (39.83, 54.09)	3618.47 (3184.91, 4132.39)	22.03 (16.52, 28.41)	3809.06 (3365.95, 4313.78)	-1.83 (-4.36, 1.1)	-0.0739 (-0.1826, 0.0349)
	Gabon	1750038.04 (1566261.77, 1939240.2	76.5	40509.54 (35503.98, 46353.68)	100.69 (93.85, 108.1)	2314.78 (2028.75, 2648.	13.71 (9.83, 17.9)	2802.2 (2470.96, 3184.	-0.14 (-2.89, 2.8)	-0.001 (-0.0896, 0.0876)

		2)				72)		14)		
	Georgia	3664751.94 (3306197.83, 4043347.78)	-33.47	137699.54 (122075.26, 156632.41)	-20.52 (-23.65, -16.84)	3757.4 (3331.06, 4274.02)	19.47 (14.77, 25)	2973.43 (2614.48, 3377.53)	1 (-2.26, 5.19)	-0.7292 (-0.7957, -0.6625)
	Grenada	103215.27 (90672.55, 115503.96)	20.48	3071.14 (2695.42, 3501.35)	49.27 (41.68, 56.51)	2975.47 (2611.46, 3392.28)	23.9 (17.6, 29.91)	2733.41 (2401.77, 3099.58)	-2.67 (-5.49, 0.28)	-0.0672 (-0.2885, 0.1546)
	Indonesia	259465835.49 (226843397.39, 291997878.71)	39.97	9234621.89 (8117808.44, 10516756.78)	66.7 (61.2, 72.68)	3559.09 (3128.66, 4053.23)	19.1 (15.17, 23.37)	3575.98 (3172.39, 4034.52)	-6.89 (-7.81, -5.98)	-0.1905 (-0.2096, -0.1713)
	Iran (Islamic Republic of)	84297882.14 (77330566.63, 91935910.25)	44	3050709.48 (2686650.2, 3487116.35)	94.97 (85.08, 104.65)	3618.96 (3187.09, 4136.66)	35.4 (28.52, 42.12)	3492.98 (3092.55, 3949.54)	-8.35 (-9.18, -7.47)	-0.3121 (-0.3535, -0.2707)
	Iraq	42119489.94 (31429294.46, 52981880.49)	139.36	1079909.42 (949881.57, 1240335.86)	188.35 (177.96, 198.41)	2563.92 (2255.21, 2944.8)	20.46 (16.13, 24.67)	3058.3 (2709.35, 3476.33)	-2.65 (-5.41, 0.05)	-0.0899 (-0.1096, -0.0702)
	Jamaica	2810753.78 (2482603.38, 3132031.36)	18.91	83800.68 (73840.97, 95494.05)	46.88 (40.65, 53.53)	2981.43 (2627.09, 3397.45)	23.52 (18.28, 29.11)	2796.26 (2462.37, 3192.45)	-0.91 (-3.67, 2.1)	-0.033 (-0.0748, 0.0089)
	Mauritius	1276662.67 (1113654.63, 1442941.5	16.05	50017.82 (44046.97, 56734.07)	61.18 (51.15, 72.06)	3917.86 (3450.16, 4443.	38.88 (30.25, 48.26)	3208.38 (2829.87, 3612.	0 (-3.34, 3.3)	-0.0799 (-0.1422, -0.017

		1)				94)		93)		6)
	Mexico	124940174.75 (108607131.68, 140630348.19)	46.15	3491462.68 (3057931.37, 3987524.01)	91.71 (83.61, 100.29)	2794.51 (2447.52, 3191.55)	31.17 (25.63, 37.04)	2736.35 (2395, 3123.41)	0.57 (-0.3, 1.44)	-0.173 (-0.1879, -0.158)
	Panama	4160456.85 (3659254.67, 4679228.59)	74.19	120255.06 (106249.29, 136497.23)	105.42 (96.24, 114.66)	2890.43 (2553.79, 3280.82)	17.93 (12.66, 23.23)	2863.19 (2531.52, 3255.79)	-0.04 (-3.06, 2.95)	0.0097 (-0.033, 0.0523)
	Paraguay	6930455.03 (5699197.99, 8110996.52)	71.31	203739.66 (179054.98, 232132.29)	112.17 (103.47, 121.13)	2939.77 (2583.6, 3349.45)	23.85 (18.77, 29.08)	3063.81 (2697.36, 3465.27)	1.41 (-2.02, 4.82)	0.0058 (-0.0291, 0.0407)
	Peru	33995397.38 (31120105.03, 36626225.84)	56.44	889037.94 (780586.31, 1012187.4)	102.53 (92.93, 111.16)	2615.17 (2296.15, 2977.42)	29.45 (23.32, 34.97)	2610.81 (2295.83, 2958.79)	1.45 (-1.61, 4.54)	0.0777 (0.0617, 0.0937)
	Republic of Moldova	3688191.08 (3095660.79, 4327373.61)	-17.05	168818.81 (149259.51, 191344.22)	2.49 (-1.45, 6.88)	4577.28 (4046.96, 5188.02)	23.56 (18.8, 28.85)	3574.37 (3159.3, 4051.64)	-2.63 (-5.49, 0.44)	-0.0882 (-0.1204, -0.056)
	Saint Lucia	174625.74 (153612.32, 195040.85)	27.09	5651.94 (4992.62, 6439.95)	77.2 (66.45, 87.62)	3236.6 (2859.04, 3687.86)	39.43 (30.97, 47.63)	2785.93 (2464.12, 3163.21)	-3.27 (-6.19, -0.32)	-0.1206 (-0.343, 0.1023)
	Saint Vincent and the Grenadines	113143.66 (101217.52, 125637.39)	2.8	3427.49 (3024.05, 3893.4)	39.22 (31.38, 47.27)	3029.32 (2672.75, 3441.	35.43 (27.8, 43.26)	2718.85 (2397.95, 3075.	-1.58 (-4.3, 1.48)	-0.0613 (-0.3046, 0.182

						11)		67)		6)
	Samoa	211353.86 (192999.8, 229266.17)	29.16	7164.05 (6315.71, 8068.73)	42.7 (37.48, 48.03)	3389.6 (2988.22, 3817.64)	10.48 (6.44, 14.61)	3970.79 (3524.44, 4448.23)	-3.62 (-6.39, -0.43)	-0.1631 (-0.3394, 0.0134)
	South Africa	55588424.52 (49169681.77, 62724694.2)	50.93	1272855.21 (1122861.09, 1454733.21)	75.28 (70.26, 80.22)	2289.78 (2019.95, 2616.97)	16.13 (12.81, 19.4)	2423.99 (2151.43, 2760.58)	-7.15 (-8.28, -5.92)	-0.153 (-0.166, -0.14)
	Sri Lanka	21854451.85 (19445063.79, 24139978.54)	26.9	773104.95 (684744.9, 873795.25)	69.19 (61.24, 78.36)	3537.52 (3133.21, 3998.25)	33.32 (27.06, 40.55)	3201.15 (2843.19, 3608.64)	1.59 (-1.57, 4.85)	-0.0468 (-0.0647, -0.029)
	Suriname	575887.92 (512163.63, 648630.11)	48.96	16785.15 (14783.2, 19210.6)	83.26 (74.71, 91.78)	2914.66 (2567.03, 3335.82)	23.02 (17.28, 28.75)	2769.29 (2444.14, 3146.84)	0.55 (-2.88, 3.56)	0.0336 (-0.0759, 0.1433)
	Thailand	70111586.24 (61328964.9, 78909536.42)	23.27	2817816.75 (2497444.9, 3209591.55)	88.52 (75.99, 103.55)	4019.05 (3562.1, 4577.83)	52.93 (42.76, 65.12)	3147.61 (2787.79, 3553.42)	2.94 (-1.22, 8.86)	0.0985 (0.0817, 0.1154)
	Tokelau	1410.79 (1283.13, 1528.53)	-16.38	51.28 (45.54, 58.18)	-4.49 (-8.18, -0.54)	3634.5 (3227.72, 4123.89)	14.23 (9.81, 18.95)	3840.67 (3409.08, 4346.68)	-2.05 (-4.77, 0.99)	-0.0677 (-1.6228, 1.5119)
	Tonga	102350.08 (93644.1, 111194.72)	5.75	3501.05 (3093.96, 3955.11)	21.24 (17.09, 25.67)	3420.66 (3022.92,	14.66 (10.73, 18.84)	3920.67 (3480.98,	-1.06 (-3.45, 1.64)	-0.0606 (-0.2759,

						3864.29)		4429.17)		0.1552)
	Tunisia	11571604.49 (10423733.2, 12757922.81)	37.12	382940.09 (337332.72, 438013)	90.18 (80.65, 100.55)	3309.31 (2915.18, 3785.24)	38.7 (31.75, 46.26)	3009.42 (2660.61, 3420.74)	-0.12 (-3.2, 3.47)	-0.0746 (-0.1091, -0.04)
	Turkmenistan	5083080.25 (4614049.33, 5544852.77)	37.19	147762.59 (129319.23, 168955.19)	70.85 (62.74, 79.85)	2906.95 (2544.11, 3323.87)	24.54 (18.63, 31.1)	3124.17 (2752.74, 3551.13)	-2.72 (-5.53, 0.26)	-0.0989 (-0.1412, -0.0567)
	Uruguay	3436136.7 (3031174.69, 3876964.06)	9.45	97041.82 (85255.35, 110066.53)	24.48 (19.74, 29.46)	2824.15 (2481.14, 3203.21)	13.73 (9.4, 18.28)	2427.46 (2118.86, 2753.93)	4.19 (0.37, 7.98)	-0.0067 (-0.2537, 0.2409)
	Uzbekistan	33677096.35 (25411000.8, 42319371.37)	60.77	922383.55 (807959.5, 1063274.59)	93.5 (85.1, 103.15)	2738.9 (2399.14, 3157.26)	20.36 (15.14, 26.36)	3106.12 (2751.43, 3530.75)	-0.39 (-3.22, 2.69)	-0.0186 (-0.0384, 0.0012)
**High-middle**	American Samoa	55504.95 (48382.93, 62763.58)	14.61	2020.89 (1787.38, 2288.67)	47.44 (40.67, 54.25)	3640.93 (3220.21, 4123.37)	28.65 (22.74, 34.59)	3840.08 (3422.71, 4330.18)	-0.16 (-2.86, 2.5)	-0.0127 (-0.3808, 0.3568)
	Antigua and Barbuda	88489.35 (77626.14, 98864.06)	45.73	2745.05 (2413.53, 3135.52)	77.28 (68.21, 87.31)	3102.13 (2727.48, 3543.39)	21.66 (15.43, 28.54)	2720.99 (2402.38, 3084.44)	-1.25 (-4.23, 1.66)	-0.0683 (-0.3156, 0.1797)
	Argentina	45115284.36 (39507182.89, 51073380.88)	36.21	1165772.86 (1021129.24, 1329341.74)	49.86 (45.09, 54.7)	2583.99 (2263.38, 2946.54)	10.01 (6.51, 13.57)	2393.08 (2089.79, 2725.79)	-0.46 (-3.62, 2.67)	-0.0739 (-0.0926, -0.0552)

	Bahamas	376940.28 (330381.27, 424688.64)	46.98	11256.52 (9853.17, 12828.75)	82.35 (72.39, 92.02)	2986.29 (2613.99, 3403.39)	24.06 (17.29, 30.65)	2721.54 (2389.22, 3092.43)	-2.02 (-4.99, 0.76)	-0.0866 (-0.2363, 0.0633)
	Bahrain	1442691.32 (1254828.57, 1605903.36)	183.97	51265.09 (43818.11, 59720.79)	298.09 (264.84, 333.98)	3553.43 (3037.25, 4139.54)	40.19 (28.48, 52.83)	3082.56 (2707.65, 3503.97)	-1 (-4.25, 2.14)	-0.0254 (-0.3392, 0.2894)
	Barbados	297770.8 (263600.9, 334647.28)	17.28	10332.16 (9127.39, 11793.4)	41.23 (33.89, 48.65)	3469.84 (3065.24, 3960.56)	20.42 (14.16, 26.75)	2742.61 (2407.8, 3126.91)	-1.09 (-4.22, 1.8)	-0.0413 (-0.1615, 0.079)
	Belarus	9500785.27 (8345414.33, 10677716.21)	-9.26	450470.9 (399088.95, 513132.54)	4.5 (0.64, 8.51)	4741.41 (4200.59, 5400.95)	15.17 (10.91, 19.58)	3660.82 (3237.43, 4136.45)	-0.89 (-3.64, 1.56)	-0.0082 (-0.0246, 0.0083)
	Bosnia and Herzegovina	3299982.33 (2949575.52, 3649228.46)	-27.28	163977.61 (145144.91, 187174.5)	-5.75 (-11.24, 0.03)	4969.05 (4398.35, 5671.98)	29.6 (22.06, 37.56)	3793.67 (3352.83, 4289.48)	1.06 (-2.1, 4.31)	-0.0291 (-0.0634, 0.0052)
	Bulgaria	6934624.61 (6359976.35, 7553855.84)	-20.12	382244.33 (337709.61, 433890.99)	-9.58 (-12.97, -6.01)	5512.11 (4869.9, 6256.88)	13.2 (8.96, 17.66)	4077.27 (3620.81, 4647.12)	-1.06 (-4.01, 1.73)	-0.0312 (-0.0566, -0.0059)
	Chile	18198359.04 (16753483.01, 19617222.72)	37.03	496800.7 (435002.97, 570421.3)	69.93 (60.54, 78.87)	2729.92 (2390.34, 3134.47)	24.01 (17.15, 30.53)	2378.95 (2066.89, 2712.05)	0.09 (-2.97, 3.2)	-0.0432 (-0.0662, -0.0202)

	Cook Islands	17986.67 (16598.77, 19575.23)	-5.44	803.96 (714.5, 908.32)	32.35 (25.17, 39.87)	4469.75 (3972.4, 5049.96)	39.97 (32.37, 47.92)	3841.17 (3413.96, 4324.33)	0.04 (-2.47, 2.81)	0.0083 (-0.4526, 0.4713)
	Croatia	4247902.46 (3748359.98, 4764236.58)	-13.32	231826.69 (205231.68, 262408.68)	-3.37 (-8.88, 2.8)	5457.44 (4831.37, 6177.37)	11.47 (5.12, 18.6)	4089.5 (3602.05, 4608.56)	-3.61 (-8.05, 0.95)	-0.134 (-0.1776, -0.0904)
	Dominica	68681.45 (60139.47, 77088.6)	-7.19	2144.3 (1890.43, 2428.35)	12.21 (7.02, 17.49)	3122.09 (2752.46, 3535.67)	20.9 (15.31, 26.6)	2738.98 (2409, 3107.63)	-2.65 (-5.48, 0.54)	-0.1219 (-0.3692, 0.126)
	Greece	10337171.62 (9070617.15, 11489318.75)	-0.5	365188.03 (320546.94, 414544.88)	12.19 (7.69, 17.57)	3532.77 (3100.92, 4010.24)	12.76 (8.23, 18.16)	2714.68 (2371.48, 3090.64)	0.56 (-2.5, 3.87)	-0.1626 (-0.1851, -0.14)
	Greenland	56188.39 (51528.46, 60807.66)	1.1	1851.05 (1613.56, 2106.98)	14.67 (6.18, 24.42)	3294.35 (2871.7, 3749.84)	13.43 (5.02, 23.07)	2930.15 (2566.62, 3318.03)	-2.81 (-5.78, 0.35)	-0.149 (-0.5849, 0.2888)
	Hungary	9674412.78 (8515522.28, 10788963.18)	-6.91	528781.02 (469002.88, 599707.39)	7.96 (4.07, 12.32)	5465.77 (4847.87, 6198.9)	15.98 (11.79, 20.66)	4107.21 (3649.54, 4625.92)	2.42 (-1.03, 5.72)	-0.0188 (-0.0358, -0.0019)
	Israel	9309583.14 (8164696.78, 10550907.48)	87.62	271895.38 (239126.45, 306401.15)	99.53 (90.99, 107.87)	2920.6 (2568.61, 3291.24)	6.35 (1.79, 10.79)	2828.5 (2484.61, 3200.87)	-0.2 (-3.61, 3.05)	-0.1059 (-0.1368, -0.075)

	Italy	60313169.95 (55356079.59, 64983928.64)	6.19	2487894.09 (2186301.07, 2827095.31)	15.63 (12.03, 19.51)	4124.96 (3624.91, 4687.36)	8.89 (5.5, 12.55)	3112.64 (2730.23, 3519.07)	-1.5 (-2.5, -0.47)	-0.1674 (-0.1885, -0.1462)
	Jordan	11636716.67 (10588262.43, 12678912.33)	208.41	312023.52 (271793.21, 358356.11)	298.62 (279.81, 317.65)	2681.37 (2335.65, 3079.53)	29.25 (23.15, 35.42)	3094.64 (2725.9, 3511.31)	-1.05 (-4.09, 1.86)	-0.0493 (-0.1056, 0.0071)
	Kazakhstan	18392068.15 (16794137.15, 19921601.72)	12.37	600890.89 (532942.73, 682777.12)	24.37 (19.94, 29.64)	3267.12 (2897.68, 3712.35)	10.68 (6.74, 15.37)	3232.34 (2875.6, 3654.17)	-1.75 (-4.52, 1.54)	-0.1038 (-0.1215, -0.0862)
	Lebanon	5177068.9 (4455291.49, 5928541.91)	58.07	150707.83 (132418.64, 171751.01)	95.55 (88.15, 103.62)	2911.06 (2557.79, 3317.53)	23.71 (19.02, 28.82)	2830.32 (2484.6, 3214.28)	-1.05 (-4.05, 1.76)	-0.0273 (-0.0707, 0.0162)
	Libya	6735543.07 (5705382.3, 7670829.33)	58.97	210984.85 (183586.05, 243607.58)	131.71 (118.01, 146.71)	3132.41 (2725.63, 3616.75)	45.75 (37.13, 55.19)	3041.97 (2684.58, 3459.01)	-2.16 (-4.97, 1.07)	-0.0685 (-0.1065, -0.0305)
	Malaysia	31301402.28 (27339261.56, 35190541.52)	77.29	1040641.12 (917613, 1183356.62)	119.47 (106.54, 132.47)	3324.58 (2931.54, 3780.52)	23.79 (16.5, 31.12)	3371.55 (2991.28, 3810.94)	-1.9 (-6.9, 2.63)	-0.1971 (-0.2139, -0.1803)
	Malta	439220.75 (389214.99, 489625.59)	18.49	16586.21 (14627.18, 18842.08)	40.4 (33.1, 48.73)	3776.28 (3330.26, 4289.89)	18.49 (12.33, 25.53)	2974.23 (2617.07, 3366.03)	0.49 (-2.53, 3.51)	-0.1119 (-0.2115, -0.0122)

	Montenegro	620339.57 (545880.74, 695553.31)	-0.86	30722.26 (27160.42, 35046.55)	17.08 (12.21, 22.2)	4952.49 (4378.31, 5649.57)	18.09 (13.18, 23.26)	4023.53 (3544.21, 4572.29)	-1.48 (-4.54, 1.4)	-0.0362 (-0.1061, 0.0338)
	Niue	1671.55 (1472.19, 1876.65)	-28.15	73 (64.72, 82.37)	-12.09 (-15.57, -8.76)	4367.31 (3871.78, 4927.88)	22.36 (17.52, 26.99)	3841.91 (3397.92, 4327.73)	-1.25 (-4.18, 1.67)	-0.0474 (-1.2057, 1.1245)
	North Macedonia	2152731.06 (1785469.3, 2527621.99)	6.81	100944.95 (89074.61, 115077.21)	29.78 (23.91, 35.31)	4689.16 (4137.75, 5345.64)	21.5 (16, 26.68)	3780.44 (3345.16, 4284.93)	-1.32 (-4.27, 1.65)	-0.0306 (-0.0806, 0.0194)
	Northern Mariana Islands	42494.48 (37225.71, 47877)	-6.39	1910.01 (1678.65, 2196)	34.48 (20.35, 49.84)	4494.72 (3950.29, 5167.74)	43.66 (28.57, 60.07)	3758.31 (3343.89, 4227.99)	-0.67 (-3.22, 1.97)	-0.0303 (-0.602, 0.5448)
	Oman	4583998.58 (4209600.34, 4952284.8)	135.9	133636.21 (113171.15, 158197.12)	211.42 (193.51, 229.3)	2915.28 (2468.83, 3451.07)	32.01 (24.42, 39.59)	3087.16 (2716.54, 3502.32)	-0.94 (-4.04, 2.45)	-0.026 (-0.1328, 0.0809)
	Palau	18007.43 (16271.85, 19568.01)	16.9	811.57 (714.27, 928.63)	59.72 (49.82, 70.49)	4506.87 (3966.54, 5156.9)	36.63 (28.16, 45.84)	4078.44 (3602.04, 4623.92)	-1.25 (-3.7, 1.52)	-0.0379 (-0.6739, 0.6023)
	Poland	38434444.92 (35378980.97, 41364935.33)	0.72	2090331.69 (1851196.25, 2364558.98)	17.53 (13.73, 21.2)	5438.69 (4816.5, 6152.19)	16.68 (12.91, 20.33)	4179.43 (3702.2, 4717.38)	-3.36 (-4.27, -2.42)	-0.1315 (-0.1401, -0.123)

	Portugal	10651262.92 (9433153.42, 11909037.37)	5.07	408355.99 (361914.03, 463668.28)	21.29 (15.39, 27.3)	3833.87 (3397.85, 4353.18)	15.44 (9.82, 21.16)	2949.18 (2604.66, 3354.47)	-0.01 (-3.24, 3.49)	-0.1268 (-0.1529, -0.1008)
	Romania	19237066.4 (17030069.96, 21542479.59)	-17.78	1044385.83 (924912.86, 1184344.08)	-5.67 (-9.99, -1.74)	5429.03 (4807.97, 6156.57)	14.72 (9.47, 19.51)	4140.4 (3651.59, 4697)	-4.54 (-7.46, -1.62)	-0.1763 (-0.1902, -0.1624)
	Saint Kitts and Nevis	59508.17 (48009.66, 70881.62)	43.9	1844.46 (1610.54, 2114.77)	78.71 (66.72, 90.74)	3099.5 (2706.42, 3553.74)	24.19 (15.86, 32.54)	2732.83 (2401.09, 3098.11)	-2.02 (-4.81, 0.8)	-0.0665 (-0.4557, 0.3242)
	Serbia	8746784.81 (7829776.92, 9730592.23)	-6.92	452981.45 (400643.55, 514424)	5.06 (0.33, 9.78)	5178.83 (4580.47, 5881.29)	12.87 (7.79, 17.95)	4160.1 (3700.62, 4674.5)	-0.62 (-3.69, 2.11)	-0.0269 (-0.0464, -0.0074)
	Seychelles	102145.3 (89531.72, 114507.69)	39.87	3672.9 (3233.6, 4200.19)	71.25 (61.5, 80.75)	3595.76 (3165.68, 4111.98)	22.43 (15.46, 29.22)	3248.3 (2879.9, 3679.23)	-3.59 (-6.38, -0.6)	-0.1287 (-0.3342, 0.0773)
	Spain	46021217.74 (42087988.43, 49981538.75)	18.67	1771794.41 (1548273.54, 2027170.14)	16.25 (6.15, 26.27)	3849.95 (3364.26, 4404.86)	-2.03 (-10.55, 6.41)	2940.45 (2568.93, 3355.52)	-14.4 (-21.54, -7.13)	-0.4365 (-0.4704, -0.4026)
	Trinidad and Tobago	1387457.08 (1226316.68, 1549440.96)	15.33	44773.25 (39514.87, 51040.94)	51.98 (43.78, 60.64)	3227 (2848.01, 3678.74)	31.78 (24.67, 39.29)	2740.62 (2413.77, 3112.19)	-1.77 (-4.51, 1.17)	-0.058 (-0.1381, 0.0221)

	Turkey	81359692.93 (71366133.7, 91236683.76)	36.12	3140049.67 (2767926.68, 3566642.52)	66.48 (55.73, 75.51)	3859.47 (3402.09, 4383.8)	22.31 (14.41, 28.94)	3452.61 (3052.34, 3899.15)	-9.53 (-13.43, -5.79)	-0.1538 (-0.17, -0.1376)
	Ukraine	44042430.65 (35745534.7, 52268015.67)	-16.37	2340533.83 (2085795.12, 2652914.7)	-6.14 (-9.21, -2.85)	5314.27 (4735.88, 6023.54)	12.23 (8.56, 16.17)	4007.15 (3543.75, 4509.24)	-1.19 (-4.23, 1.67)	-0.0538 (-0.0636, -0.0441)
	United States Virgin Islands	103985.33 (91185.3, 116693.65)	-1.91	3617.62 (3188.2, 4120.19)	29.27 (20.59, 38.81)	3478.97 (3066.01, 3962.28)	31.79 (22.95, 41.52)	2738.54 (2398.53, 3107.83)	-0.37 (-2.95, 2.32)	-0.0067 (-0.2537, 0.2409)
**High**	Andorra	83064.21 (76169.21, 89694.45)	53.56	2931.91 (2557.31, 3341.54)	76.58 (64.43, 88.54)	3529.69 (3078.72, 4022.84)	14.99 (7.08, 22.78)	2770.49 (2428.05, 3139.11)	-0.27 (-3.49, 2.89)	-0.0974 (-0.446, 0.2524)
	Australia	24568113.49 (22510057.98, 26779185.61)	45.72	796532.2 (698694.03, 904485.3)	52.08 (44.44, 60.3)	3242.14 (2843.91, 3681.54)	4.37 (-0.88, 10.01)	2723.99 (2384.08, 3095.56)	-5.29 (-9.23, -1.66)	-0.2369 (-0.2658, -0.208)
	Austria	8916185.49 (8169639.22, 9666455.67)	14.77	288856.18 (253261.76, 328518.21)	19.14 (13.27, 25.04)	3239.68 (2840.47, 3684.52)	3.81 (-1.31, 8.94)	2529.1 (2210.82, 2875.96)	-4.62 (-8.91, -0.91)	-0.1539 (-0.1829, -0.1249)
	Belgium	11419165.53 (10536910.95, 12317981.21)	14.43	393941.29 (347786.37, 447216.75)	19.53 (15.46, 23.63)	3449.83 (3045.64, 3916.37)	4.46 (0.9, 8.04)	2799.34 (2469.23, 3167.2)	-0.64 (-3.55, 2.68)	-0.107 (-0.1286, -0.0855)
	Bermuda	64030.47 (58253.56,	7.7	2435.56 (2148.37	33.07 (24.93,	3803.75	23.55 (16,	2769.05	-1.58 (-4.33,	-0.0694

		69664.24)		, 2783.12)	40.99)	(3355.23, 4346.55)	30.91)	(2431.93, 3141.97)	1.08)	(-0.3234, 0.1853)
	Brunei Darussalam	437118.57 (381965.96, 491690.53)	69.07	12058.44 (10462.13, 13916.35)	113.47 (101.5, 125.49)	2758.62 (2393.43, 3183.66)	26.26 (19.18, 33.37)	2675.12 (2343.9, 3033.82)	0.41 (-2.62, 3.41)	-0.1105 (-0.4683, 0.2485)
	Canada	36519840.27 (33331462.68, 39599795.77)	33.98	1233474.64 (1082727.78, 1400023.78)	58.82 (40.23, 78.42)	3377.55 (2964.77, 3833.6)	18.53 (4.66, 33.16)	2710.7 (2374.73, 3066.83)	4.71 (-7.16, 16.35)	0.0198 (-0.0315, 0.0712)
	Cyprus	1313477.21 (1162096.07, 1476216.53)	68.83	44543.07 (39103.54, 50713.51)	94.55 (86.26, 102.55)	3391.23 (2977.1, 3861.01)	15.24 (10.33, 19.98)	2854.19 (2510.8, 3223.26)	0.7 (-1.99, 3.78)	-0.0481 (-0.1966, 0.1007)
	Czechia	10643486.52 (9779073.75, 11500125.78)	3.36	570645.41 (503445.21, 648302.26)	16.26 (11.4, 21.37)	5361.45 (4730.08, 6091.07)	12.48 (7.78, 17.42)	4075.61 (3612.97, 4605)	-2.2 (-5.18, 0.98)	-0.08 (-0.0963, -0.0637)
	Denmark	5802733.42 (5329984.84, 6262192.39)	12.8	183946.29 (157686.81, 216998.57)	16.78 (7.75, 27.99)	3169.99 (2717.46, 3739.59)	3.53 (-4.48, 13.46)	2572.24 (2215.43, 2968.09)	-1.23 (-8.41, 6.55)	-0.249 (-0.2904, -0.2076)
	Estonia	1312361.12 (1204371.69, 1415452.05)	-16.34	63050.29 (55936.97, 71492.99)	-1.12 (-5.23, 3.11)	4804.34 (4262.32, 5447.66)	18.2 (13.28, 23.25)	3537.78 (3135.02, 3997.22)	-0.43 (-3.48, 2.86)	-0.0587 (-0.1044, -0.013)
	Finland	5534094.63 (5086540.72,	10.46	185607.93 (162853.94,	19.42 (13.94, 25.37)	3353.9 (2942.74,	8.12 (3.15, 13.5)	2624.04 (2296.73,	-1.81 (-5.38, 1.92)	-0.2107 (-0.2368,

		5991975.28)		210464.88)		3803.06)		2954.41)		-0.1846)
	France	66204314.96 (60093849.99, 72433668.54)	14.6	2301282.14 (2029674.24, 2603827.57)	24.98 (20.01, 30.03)	3476.03 (3065.77, 3933.02)	9.06 (4.72, 13.47)	2838.49 (2500.05, 3196.99)	0.65 (-2.33, 3.8)	-0.1153 (-0.1372, -0.0933)
	Germany	84914055.85 (77688648.4, 92219488.58)	6.22	3226524.26 (2841666.78, 3661208.26)	13.31 (8.36, 18.17)	3799.75 (3346.52, 4311.66)	6.68 (2.01, 11.25)	2980.56 (2620.07, 3371.43)	-0.91 (-4.02, 2.14)	-0.1446 (-0.1682, -0.121)
	Guam	170627.53 (149082.21, 191542.59)	24.76	6784.84 (6030.77, 7668.6)	57.05 (47.88, 65.86)	3976.4 (3534.46, 4494.35)	25.88 (18.52, 32.94)	3771.47 (3348.12, 4251.26)	-0.05 (-2.75, 2.71)	0.0083 (-0.2636, 0.2809)
	Iceland	344876.26 (316885.06, 373203.51)	35.79	11356.17 (9965.12, 12882.07)	45.88 (39.5, 52.9)	3292.82 (2889.48, 3735.27)	7.43 (2.73, 12.6)	2832.28 (2480.27, 3225.34)	-4.07 (-7.31, -0.69)	-0.2541 (-0.3648, -0.1432)
	Ireland	4910357.39 (4483761.43, 5355859.48)	36.35	165531.91 (145498.74, 187453.96)	52.59 (45.23, 59.66)	3371.08 (2963.1, 3817.52)	11.91 (6.51, 17.09)	2923.48 (2567.89, 3326.49)	-0.09 (-3.59, 3.76)	-0.1431 (-0.1766, -0.1095)
	Japan	127788411.13 (115774123.16, 139878470.99)	1.53	5520721.51 (4858046.79, 6269887.36)	11.51 (6.05, 17.17)	4320.21 (3801.63, 4906.46)	9.84 (4.45, 15.41)	3172.86 (2772.97, 3590.01)	-5.74 (-6.6, -4.88)	-0.1366 (-0.1555, -0.1176)
	Kuwait	4426561.24 (3926462.34, 4929514.6	151.59	147166.77 (126464.61, 171814.2	225.95 (206.99, 243.87)	3324.63 (2856.95, 3881.	29.56 (22.02, 36.68)	3140.29 (2778.55, 3552.	-0.23 (-3.25, 2.49)	-0.0153 (-0.0865, 0.055

		3)		9)		44)		84)		9)
	Latvia	1915292.09 (1760157.19, 2071422.49)	-27.96	96329.3 (85356.25, 109172.14)	-14.34 (-18.32, -10.64)	5029.48 (4456.57, 5700.03)	18.9 (13.38, 24.05)	3633.44 (3227.46, 4107.42)	-0.21 (-3.41, 2.83)	0.0091 (-0.0247, 0.0429)
	Lithuania	2794222.64 (2574822.1, 3025990.37)	-23.94	140606.25 (124831.74, 159766.3)	-7.93 (-12.29, -3.94)	5032.03 (4467.49, 5717.74)	21.04 (15.31, 26.29)	3646.5 (3232.39, 4112.41)	-2.53 (-5.54, 0.59)	-0.0896 (-0.1178, -0.0614)
	Luxembourg	618550.06 (568118.39, 666377.27)	62.24	21165.35 (18473.99, 24120.37)	66.28 (60.61, 72.8)	3421.77 (2986.66, 3899.5)	2.49 (-1.01, 6.51)	2832.96 (2478.61, 3210.1)	-0.92 (-4.23, 2.53)	-0.1537 (-0.2457, -0.0616)
	Monaco	37572.12 (34250.56, 40757.24)	23.43	1397.66 (1227.09, 1593.43)	22.47 (18.25, 27.68)	3719.95 (3265.97, 4241)	-0.78 (-4.2, 3.44)	2782.84 (2441.63, 3164.69)	-1.09 (-3.92, 2.1)	-0.1466 (-0.4294, 0.1371)
	Netherlands	17156788.07 (15675163.12, 18613311.15)	14.97	568295.15 (497155.44, 647105.09)	26.25 (19.44, 34.18)	3312.36 (2897.72, 3771.71)	9.81 (3.89, 16.71)	2635.11 (2308.28, 2979.37)	-0.83 (-4.29, 2.75)	-0.1105 (-0.1415, -0.0795)
	New Zealand	4495667.1 (4005503.84, 4968117.72)	31.56	160802.57 (140417.4, 182595.53)	42.64 (35.65, 49.71)	3576.83 (3123.39, 4061.59)	8.43 (3.11, 13.8)	2997.3 (2600.32, 3415.72)	-3.3 (-6.3, -0.56)	-0.1851 (-0.2161, -0.154)
	Norway	5348846.99 (4936723.2, 5754786.7	25.95	188926.58 (165816.93, 214390.1	28.57 (25.5, 31.91)	3532.1 (3100.05, 4008.	2.08 (-0.36, 4.73)	2892.91 (2530.6, 3277.	-2.6 (-3.79, -1.68)	-0.2001 (-0.2252, -0.175

		8)		2)		16)		74)		1)
	Puerto Rico	3521431.42 (3106914.05, 3986032.26)	-2.54	126325.88 (111861.22, 142913.51)	27.08 (20.61, 33.96)	3587.34 (3176.58, 4058.39)	30.39 (23.76, 37.46)	2702.66 (2383.87, 3065.9)	-1.13 (-4.06, 2.24)	-0.0296 (-0.0626, 0.0035)
	Qatar	2864548.18 (2595389.74, 3123703.88)	543.53	95629.87 (81013.1, 114715.57)	676.05 (634.1, 713.8)	3338.39 (2828.13, 4004.67)	20.59 (14.07, 26.46)	3184.13 (2795.35, 3622.56)	-0.37 (-3.72, 2.93)	-0.031 (-0.3385, 0.2775)
	Republic of Korea	53398251.98 (48441016.82, 58407131.73)	20.44	1846856.3 (1610991.29, 2108698.76)	54.63 (42.19, 68.09)	3458.65 (3016.94, 3949)	28.39 (18.06, 39.57)	2704.08 (2371.06, 3074.69)	-3.03 (-5.89, -0.17)	-0.2248 (-0.2562, -0.1934)
	Russian Federation	146717427.5 (128850161.23, 165171806.88)	-2.85	7129221.79 (6336404.98, 8070020.87)	9.71 (6.94, 12.67)	4859.15 (4318.78, 5500.38)	12.93 (10.08, 15.98)	3790.17 (3369.46, 4259.48)	-2.3 (-2.97, -1.69)	-0.0165 (-0.0357, 0.0026)
	San Marino	33099.76 (28891.15, 37190.51)	40.51	1138.91 (999.56, 1295.28)	51.11 (45.02, 57.3)	3440.85 (3019.83, 3913.28)	7.54 (3.2, 11.95)	2787.07 (2442.88, 3174.29)	-0.86 (-3.98, 2.21)	-0.1328 (-0.4841, 0.2198)
	Saudi Arabia	35731972.4 (31175705.65, 40192038.63)	122.7	1104495.99 (943691.81, 1291966.87)	216.33 (198.03, 235.09)	3091.06 (2641.03, 3615.72)	42.05 (33.83, 50.47)	3071.75 (2708.84, 3479.18)	-0.19 (-3.27, 2.94)	0.0042 (-0.0227, 0.0311)
	Singapore	5667451.46 (5233146.71, 6058683.7	85.99	165762.32 (143055.46, 190636.1	117.35 (103.63, 132.97)	2924.81 (2524.16, 3363.	16.86 (9.49, 25.26)	2371.31 (2056.01, 2714.	-3.78 (-7.2, -0.1)	-0.1491 (-0.1944, -0.103

		1)		2)		7)		28)		8)
	Slovakia	5437222.75 (4969934.14, 5923603.69)	2.92	279993.85 (247913.72, 320289.8)	20.46 (15.5, 26.09)	5149.57 (4559.57, 5890.69)	17.04 (12.22, 22.51)	4036.38 (3585.55, 4604.07)	-2.43 (-5.38, 0.73)	-0.1075 (-0.1309, -0.0842)
	Slovenia	2074270.57 (1914446.31, 2243231.19)	5.24	109411.92 (96908.39, 123980.72)	21.78 (16.04, 27.26)	5274.72 (4671.93, 5977.08)	15.72 (10.26, 20.93)	3918.5 (3460.43, 4434.95)	-2.8 (-6.29, 0.11)	-0.0783 (-0.1147, -0.0419)
	Sweden	10222545.75 (9312311.59, 11127452.96)	19.02	365901.12 (322555.22, 413121.38)	21.77 (16.88, 27.45)	3579.35 (3155.33, 4041.28)	2.31 (-1.8, 7.08)	2855.19 (2512.56, 3214.71)	-0.87 (-4.9, 3.46)	-0.1098 (-0.1356, -0.084)
	Switzerland	8775203.81 (8021728.49, 9564584.3)	27.82	326873.52 (289528.13, 369532.27)	21.5 (9.48, 34.19)	3724.97 (3299.39, 4211.1)	-4.94 (-14.35, 4.99)	2992.14 (2642.43, 3370.45)	-12.12 (-20.82, -3.17)	-0.1137 (-0.1565, -0.071)
	Taiwan (Province of China)	23620242.5 (21658623.59, 25443225.7)	15.79	1107010.57 (980408.95, 1264382.06)	37.43 (24.8, 51.66)	4686.7 (4150.71, 5352.96)	18.69 (7.78, 30.97)	3477.56 (3088.03, 3924.51)	-16.35 (-23.48, -9.59)	-0.0809 (-0.0936, -0.0682)
	United Arab Emirates	9241703.99 (7764641.67, 10592699.15)	393.66	330457.29 (271005.34, 401766.74)	605.35 (544.18, 666.56)	3575.72 (2932.42, 4347.32)	42.88 (30.49, 55.28)	3053.03 (2672.01, 3475.22)	-0.83 (-4.23, 2.87)	-0.0481 (-0.3258, 0.2304)
	United Kingdom	67220446.94 (60468667.95, 73925428.	16.96	2550022.38 (2248491.54, 2883906.	19.34 (15.51, 23.12)	3793.52 (3344.95, 4290.	2.03 (-1.24, 5.27)	3088.56 (2706.52, 3491.	-5.35 (-7.04, -3.78)	-0.0829 (-0.1418, -0.024

		64)		81)		22)		82)		1)
	United States of America	32797872 9.93 (28595930 3.44, 36932416 8.41)	29.33	1372960 3.68 (122999 26.6, 1528978 3.53)	31.92 (25.39, 38.5)	4186. 13 (3750 .22, 4661. 82)	2 (-3.04, 7.09)	3514. 93 (3150 .8, 3937. 23)	-6.8 (-10.17, -3.35)	-0.191 9 (-0.24 06, -0.143 1)

Notes:

† Net drifts are estimates derived from the age-period-cohort model and denotes overall annual percent change in incidence.

*Parenthesis for all GBD health estimate indicates 95% uncertainty intervals; parenthesis for net drift indicates 95% confidence intervals.

SDI= Socio-demographic Index; APC= age-period-cohort

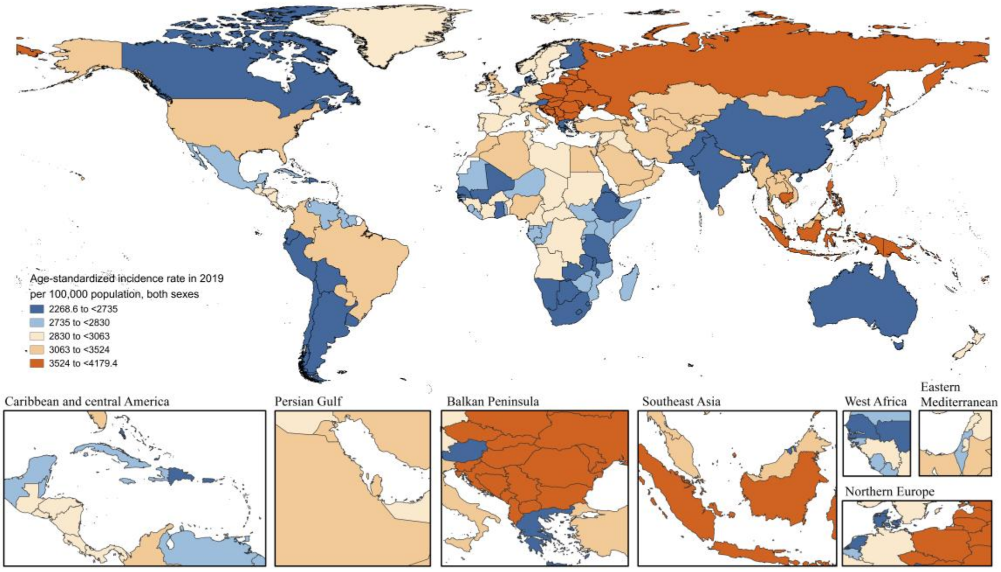

Figure S1. The world map of age-standardized incidence for low back pain, 1990-2019

Note: The global age-standardized incidence rate was 2748.90 (95%UI: 2425.77-3106.89) per 100,000 population for both sexes in 2019.

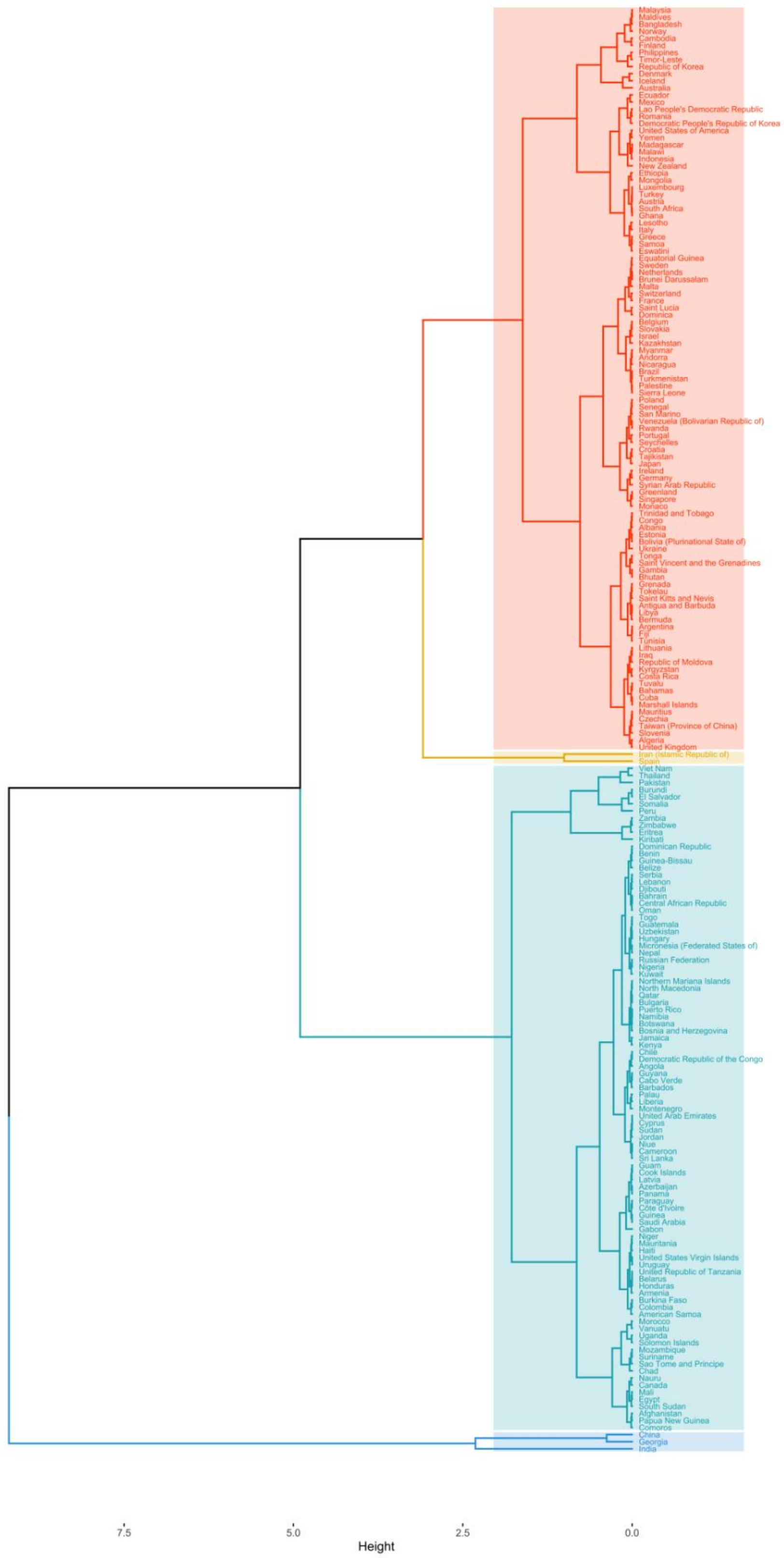

Figure S2. The cluster dendrogram of net drifts in incidence of 204 countries and territories for low back pain, 1990-2019

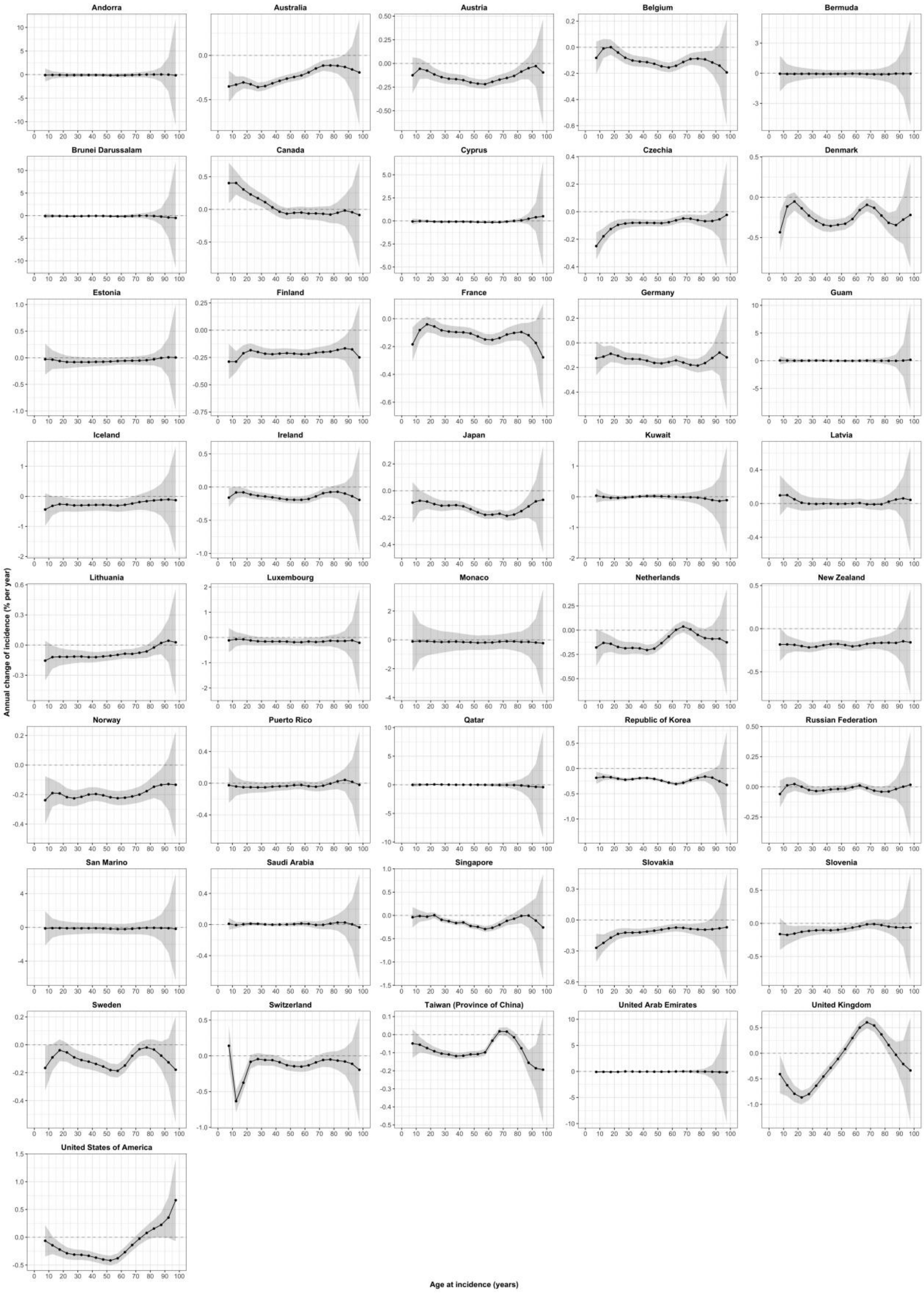

Figure S3. The local drifts of low back pain incidence in high-SDI countries, 1990-2019

The dots and shaded areas indicate the values of local drift (annual percentage change in

incidence) and its 95% CIs for low back pain in 19 age groups (5-9 to 95+ years). SDI=Socio-demographic Index.

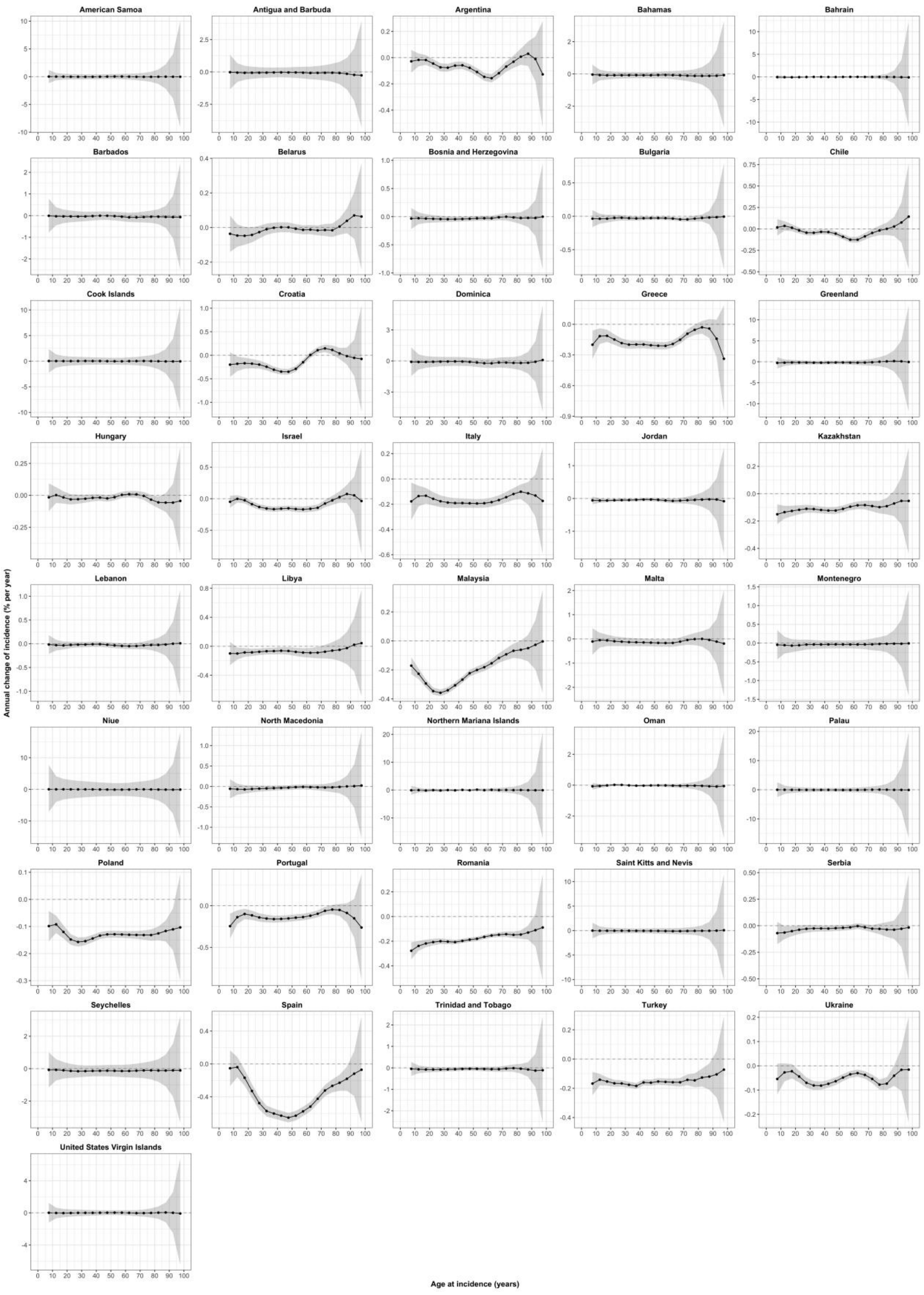

Figure S4. The local drifts of low back pain incidence in high-middle SDI countries, 1990-2019

The dots and shaded areas indicate the values of local drift (annual percentage change in incidence) and its 95% CIs for low back pain in 19 age groups (5-9 to 95+ years). SDI=Socio-demographic Index.

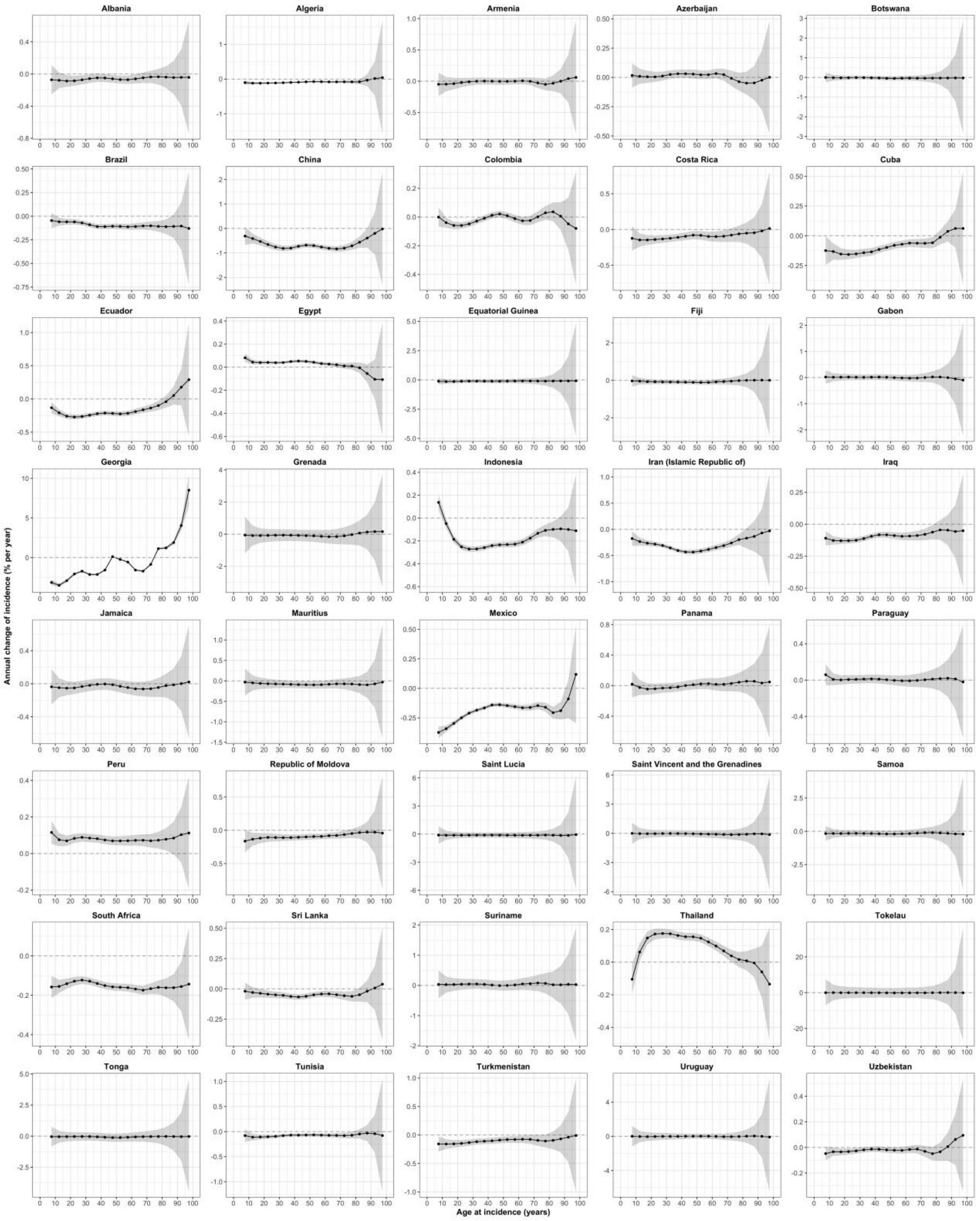

Figure S5. The local drifts of low back pain incidence in middle-SDI countries, 1990-2019

The dots and shaded areas indicate the values of local drift (annual percentage change in incidence) and its 95% CIs for low back pain in 19 age groups (5-9 to 95+ years). SDI=Socio-demographic Index.

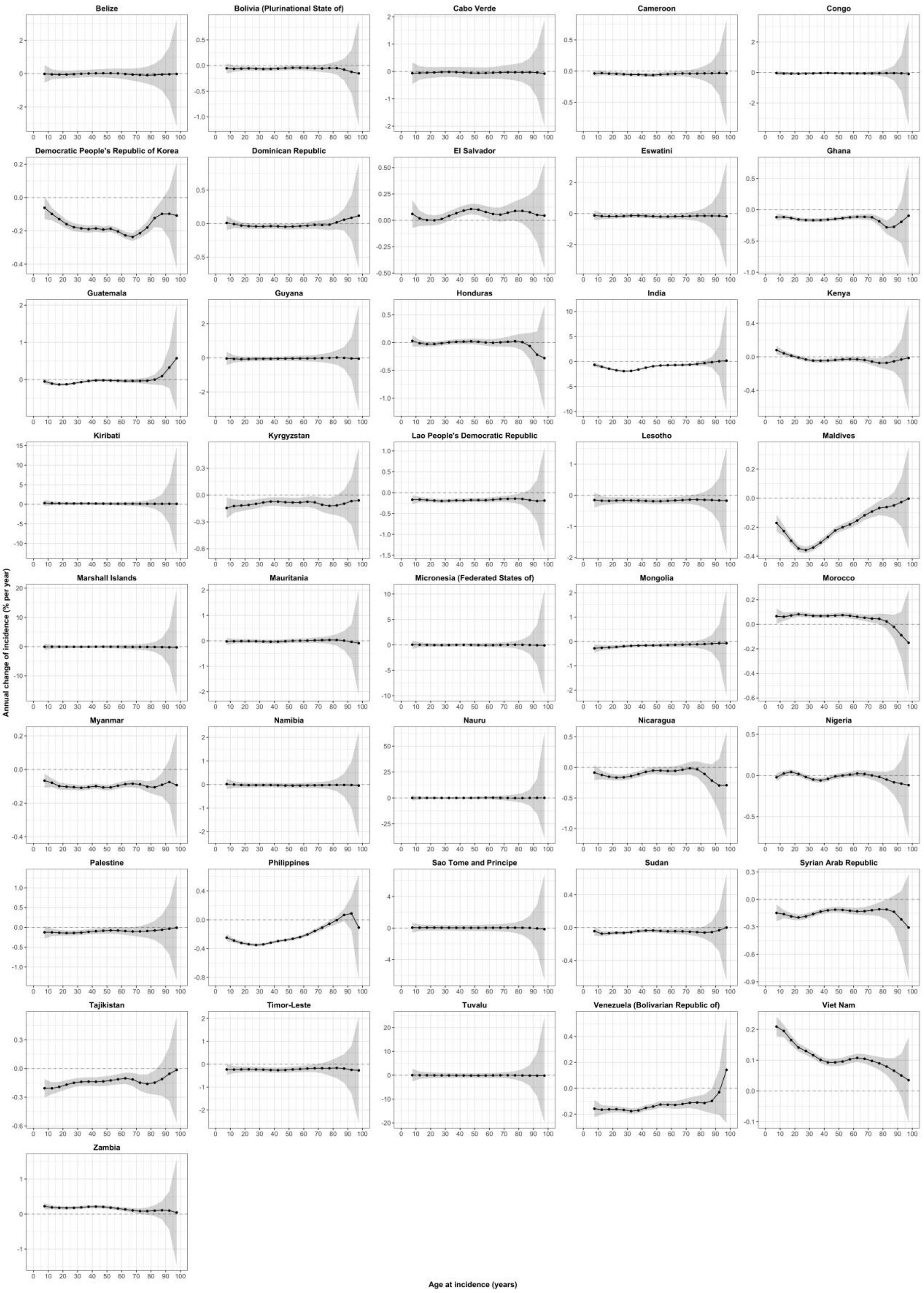

Figure S6. The local drifts of low back pain incidence in low-middle SDI countries, 1990-2019

The dots and shaded areas indicate the values of local drift (annual percentage change in

incidence) and its 95% CIs for low back pain in 19 age groups (5-9 to 95+ years). SDI=Socio-demographic Index.

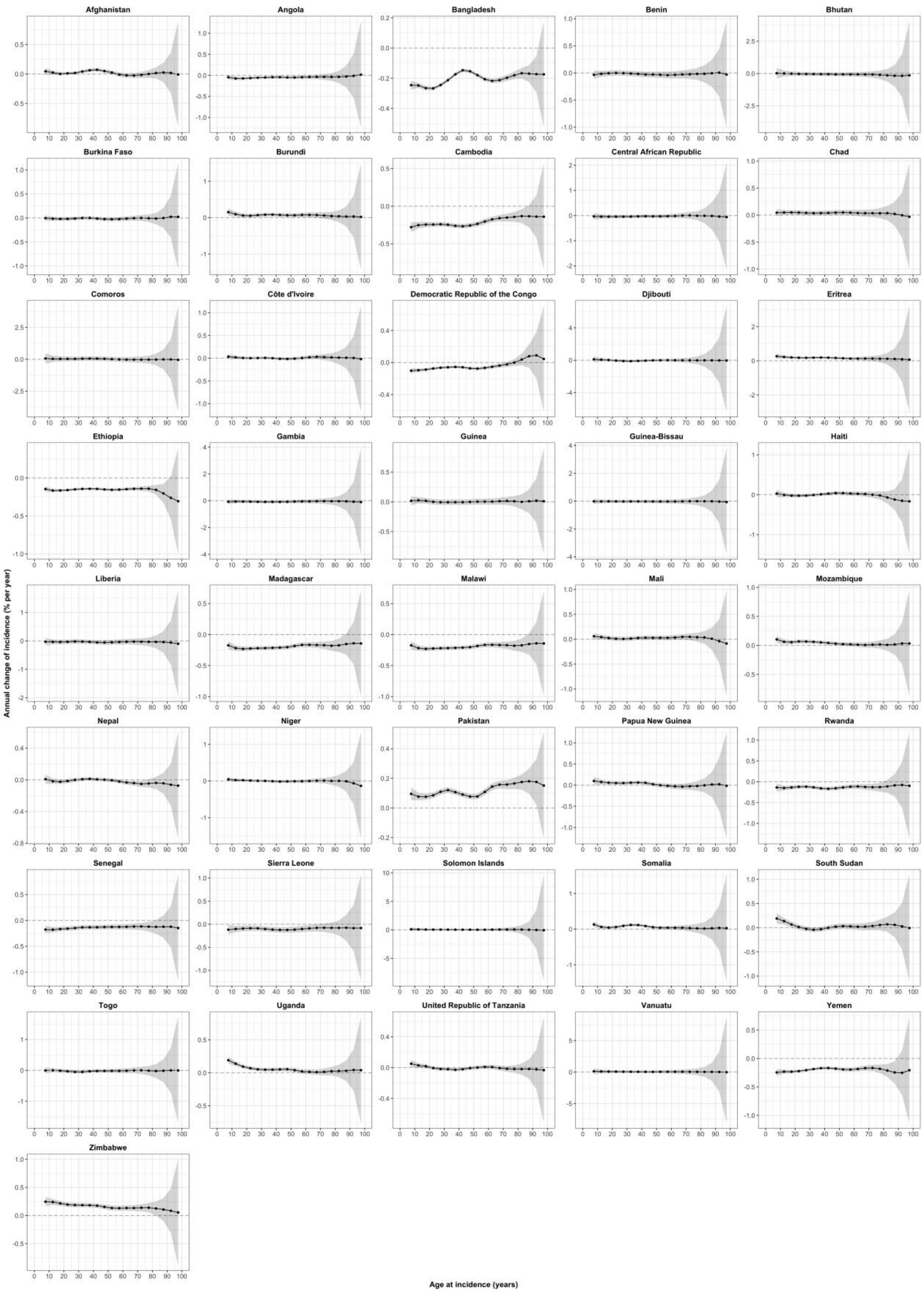

Figure S7. The local drifts of low back pain incidence in low-SDI countries, 1990-2019

The dots and shaded areas indicate the values of local drift (annual percentage change in incidence) and its 95% CIs for low back pain in 19 age groups (5-9 to 95+ years). SDI=Socio-demographic Index.

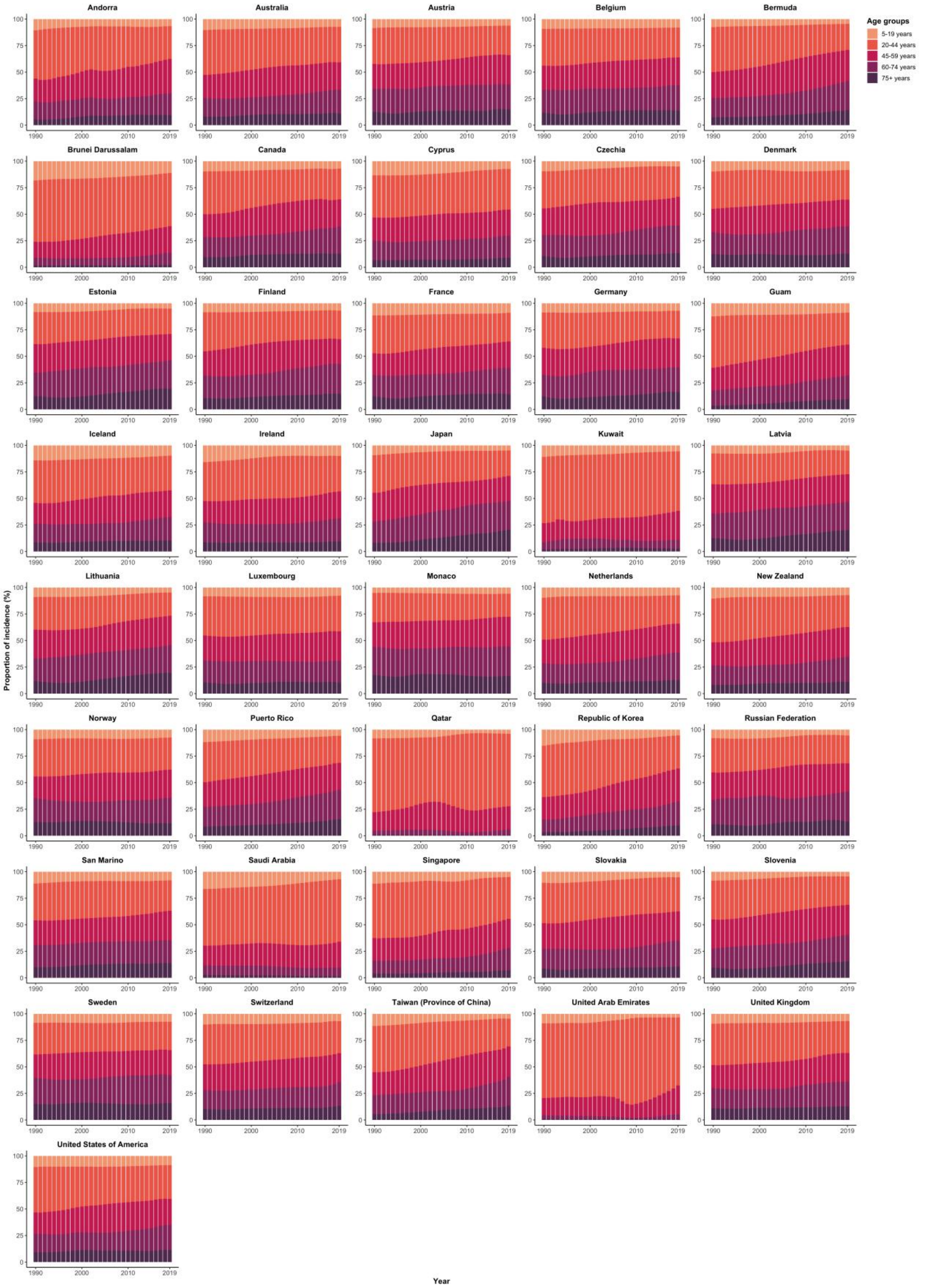

Figure S8. Age distribution of incidence from low back pain in high SDI countries, 1990-2019

Age distribution of incident cases is represented as temporal change in the relative proportion of incident cases across age groups (5-19, 20-44, 45-59, 60-74, 75+ years) from 1990 to 2019. SDI=Socio-demographic Index.

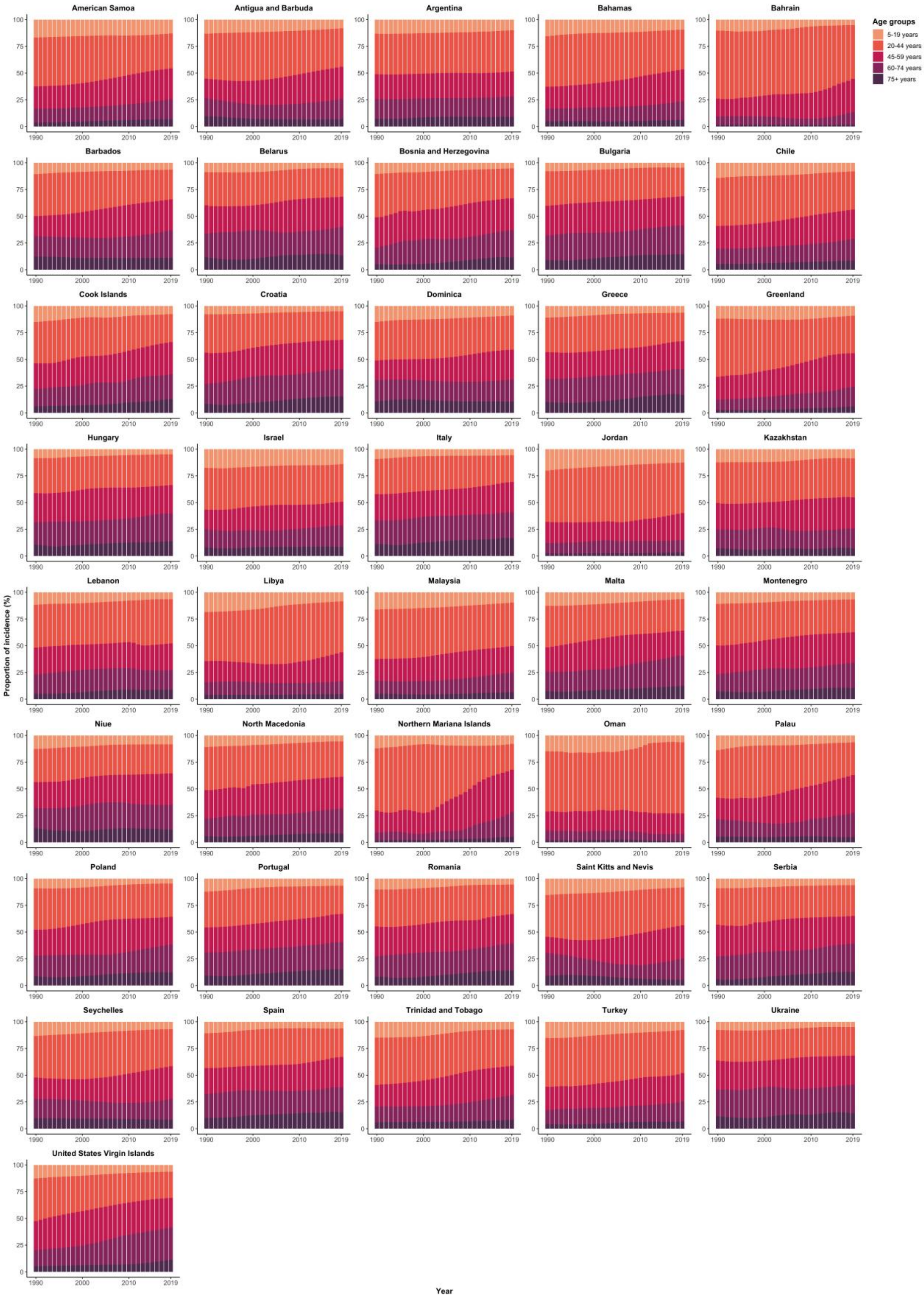

Figure S9. Age distribution of incidence from low back pain in high-middle SDI

countries, 1990-2019

Age distribution of incident cases is represented as temporal change in the relative proportion of incident cases across age groups (5-19, 20-44, 45-59, 60-74, 75+ years) from 1990 to 2019. SDI=Socio-demographic Index.

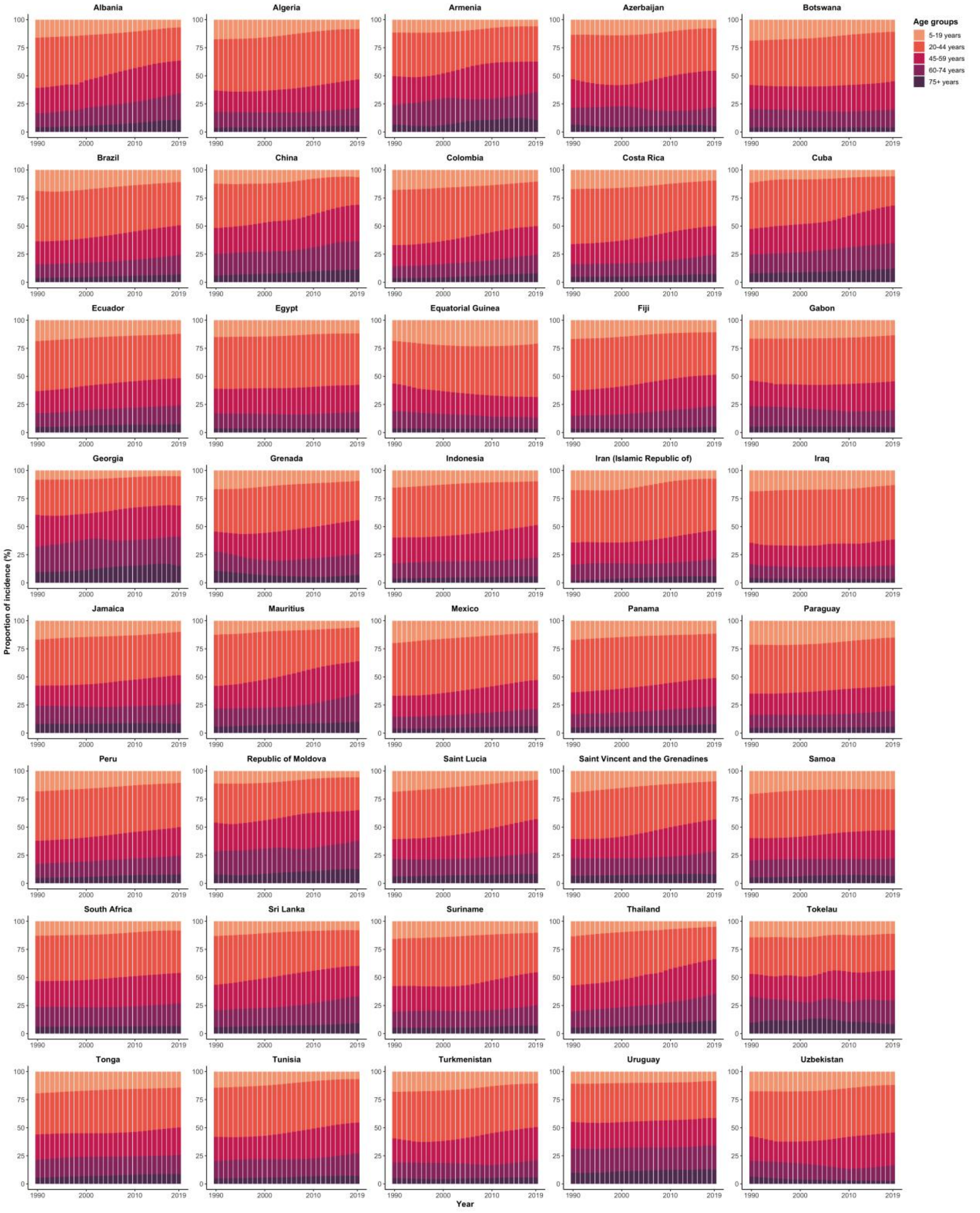

Figure S10. Age distribution of incidence from low back pain in middle SDI countries, 1990-2019

Age distribution of incident cases is represented as temporal change in the relative proportion

of incident cases across age groups (5-19, 20-44, 45-59, 60-74, 75+ years) from 1990 to 2019. SDI=Socio-demographic Index.

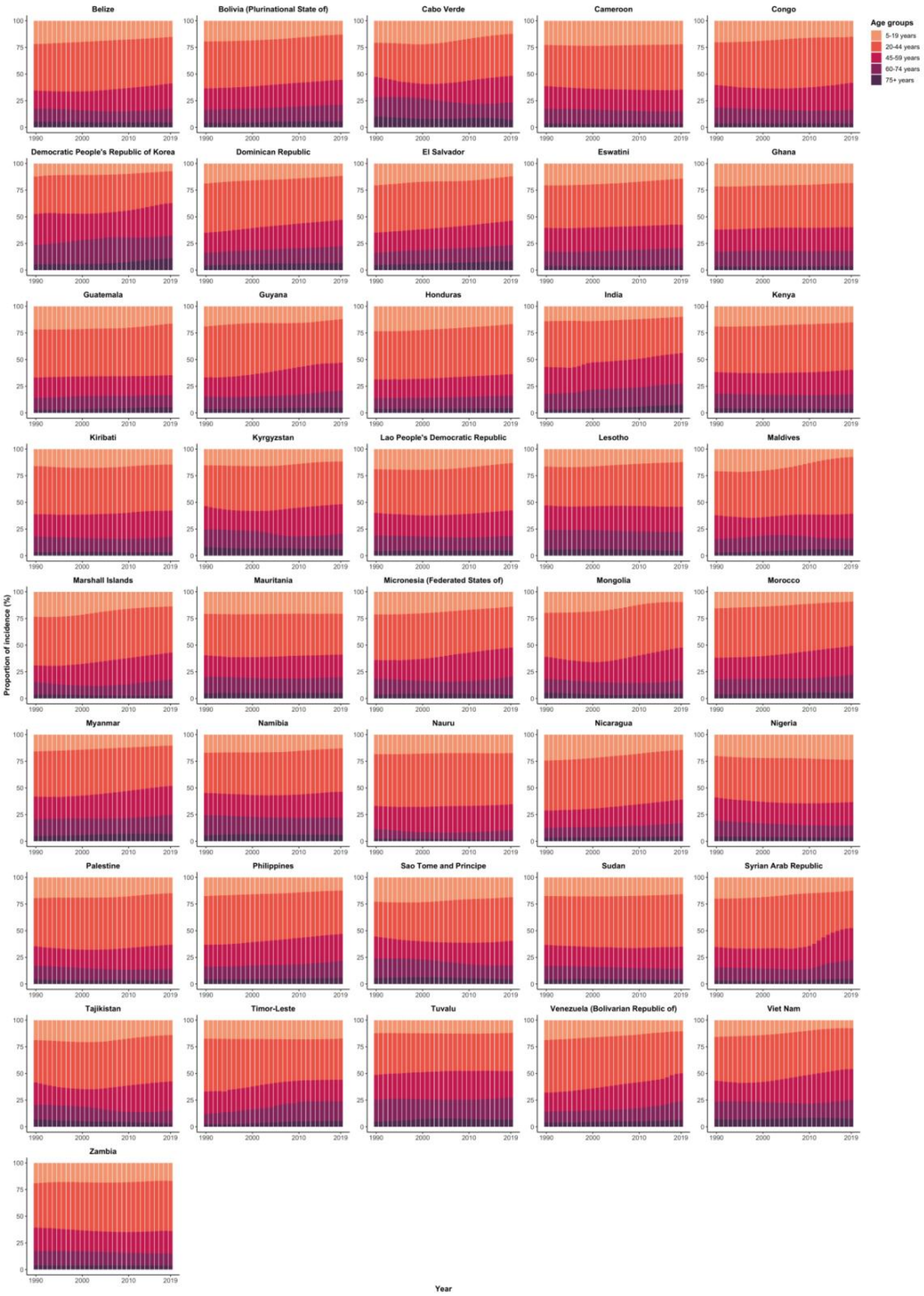

Figure S11. Age distribution of incidence from low back pain in low-middle SDI countries, 1990-2019

Age distribution of incident cases is represented as temporal change in the relative proportion

of incident cases across age groups (5-19, 20-44, 45-59, 60-74, 75+ years) from 1990 to 2019. SDI=Socio-demographic Index.

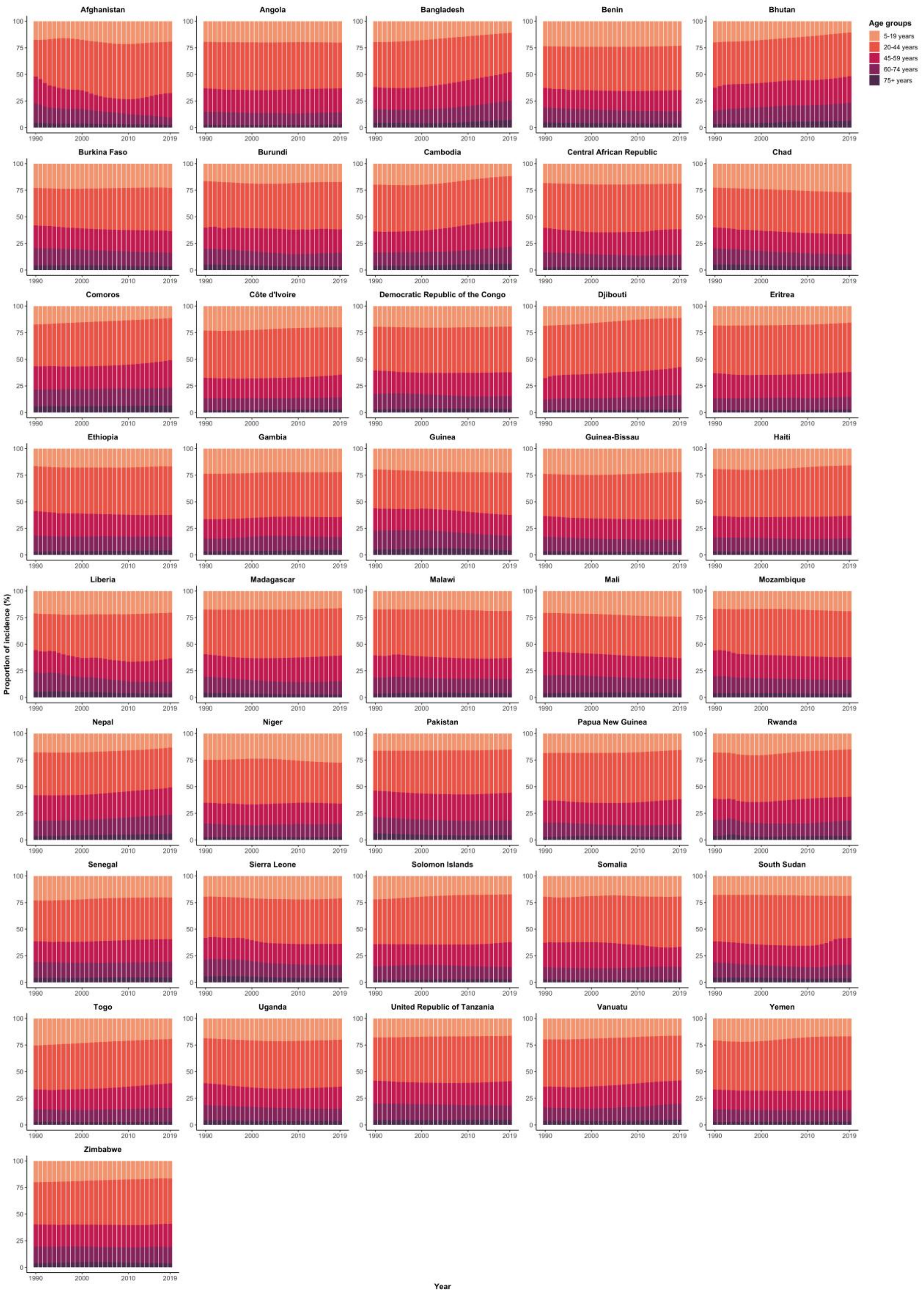

Figure S12. Age distribution of incidence from low back pain in low SDI countries, 1990-2019

Age distribution of incident cases is represented as temporal change in the relative proportion of incident cases across age groups (5-19, 20-44, 45-59, 60-74, 75+ years) from 1990 to 2019. SDI=Socio-demographic Index.

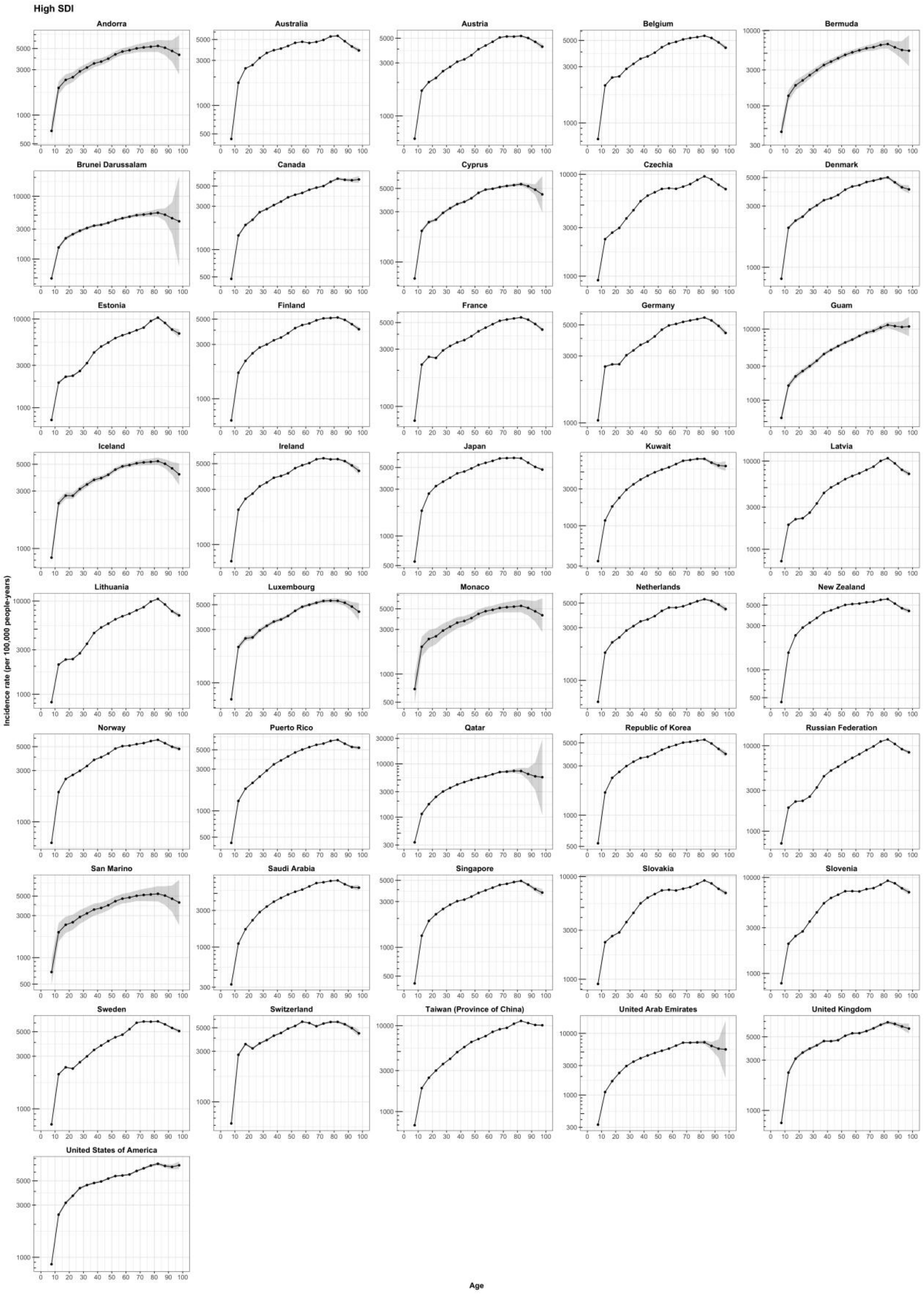

Figure S13. Age effects on low back pain incidence in high-SDI countries

Age effects indicate age-associated natural history and are shown by the fitted longitudinal age curves of incidence (per 100000 person-years) adjusted for period deviations, with the dots and shaded areas denoting incidence rates with 95% CIs. SDI=Socio-demographic Index.

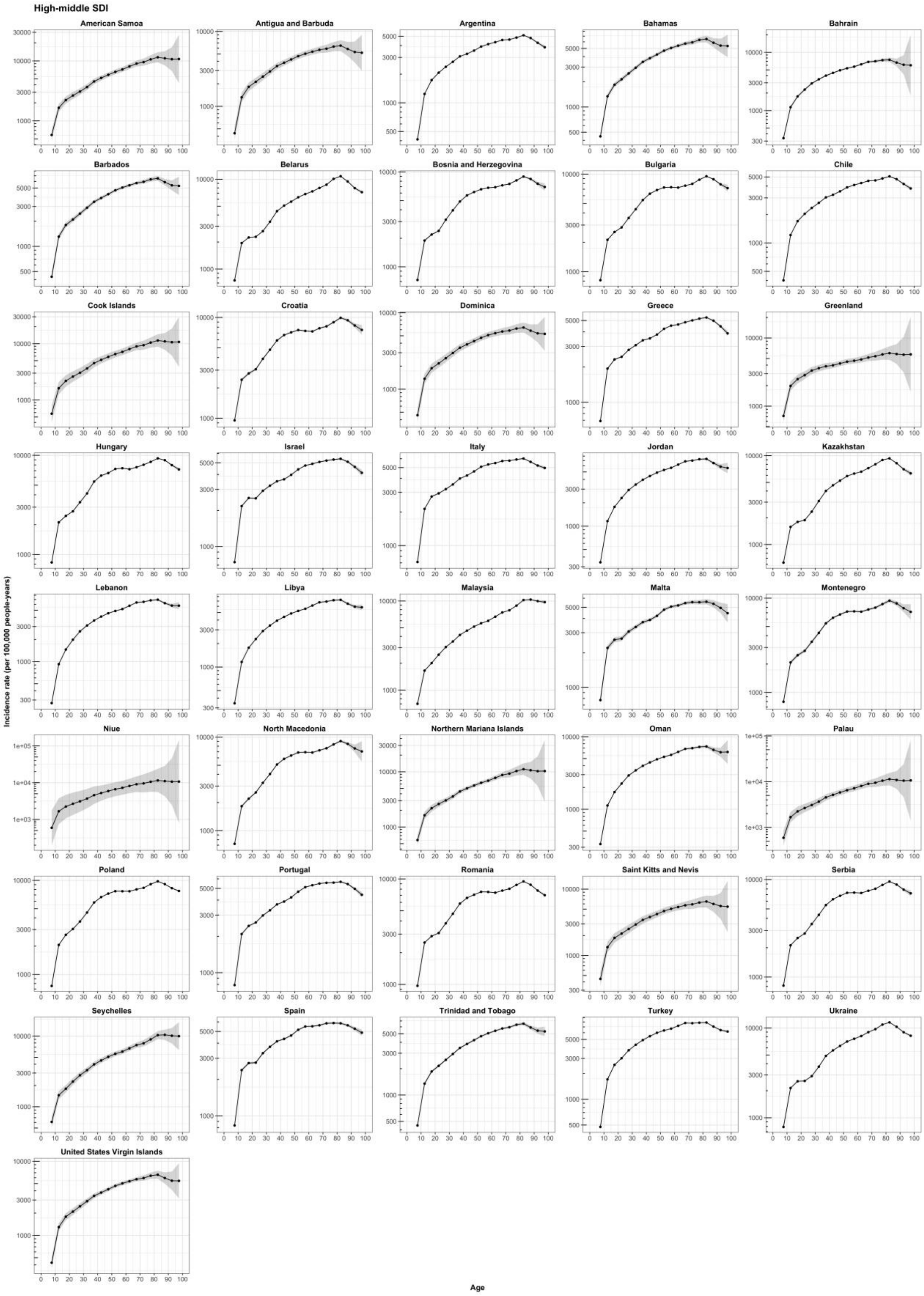

Figure S14. Age effects on low back pain incidence in high-middle SDI countries

Age effects indicate age-associated natural history and are shown by the fitted longitudinal age curves of incidence (per 100000 person-years) adjusted for period deviations, with the dots and shaded areas denoting incidence rates with 95% CIs. SDI=Socio-demographic Index.

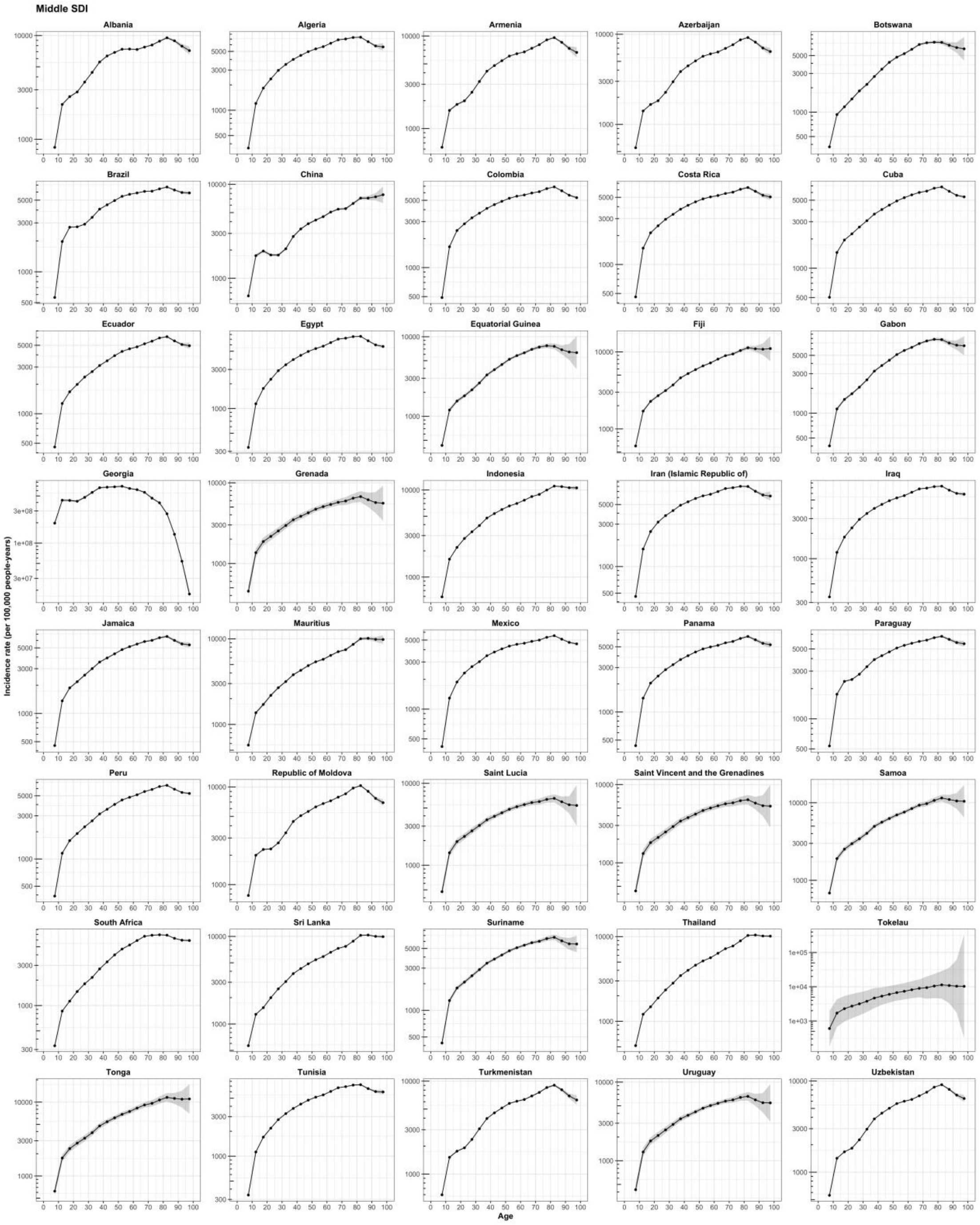

Figure S15. Age effects on low back pain incidence in middle SDI countries

Age effects indicate age-associated natural history and are shown by the fitted longitudinal age curves of incidence (per 100000 person-years) adjusted for period

deviations, with the dots and shaded areas denoting incidence rates with 95% CIs. SDI=Socio-demographic Index.

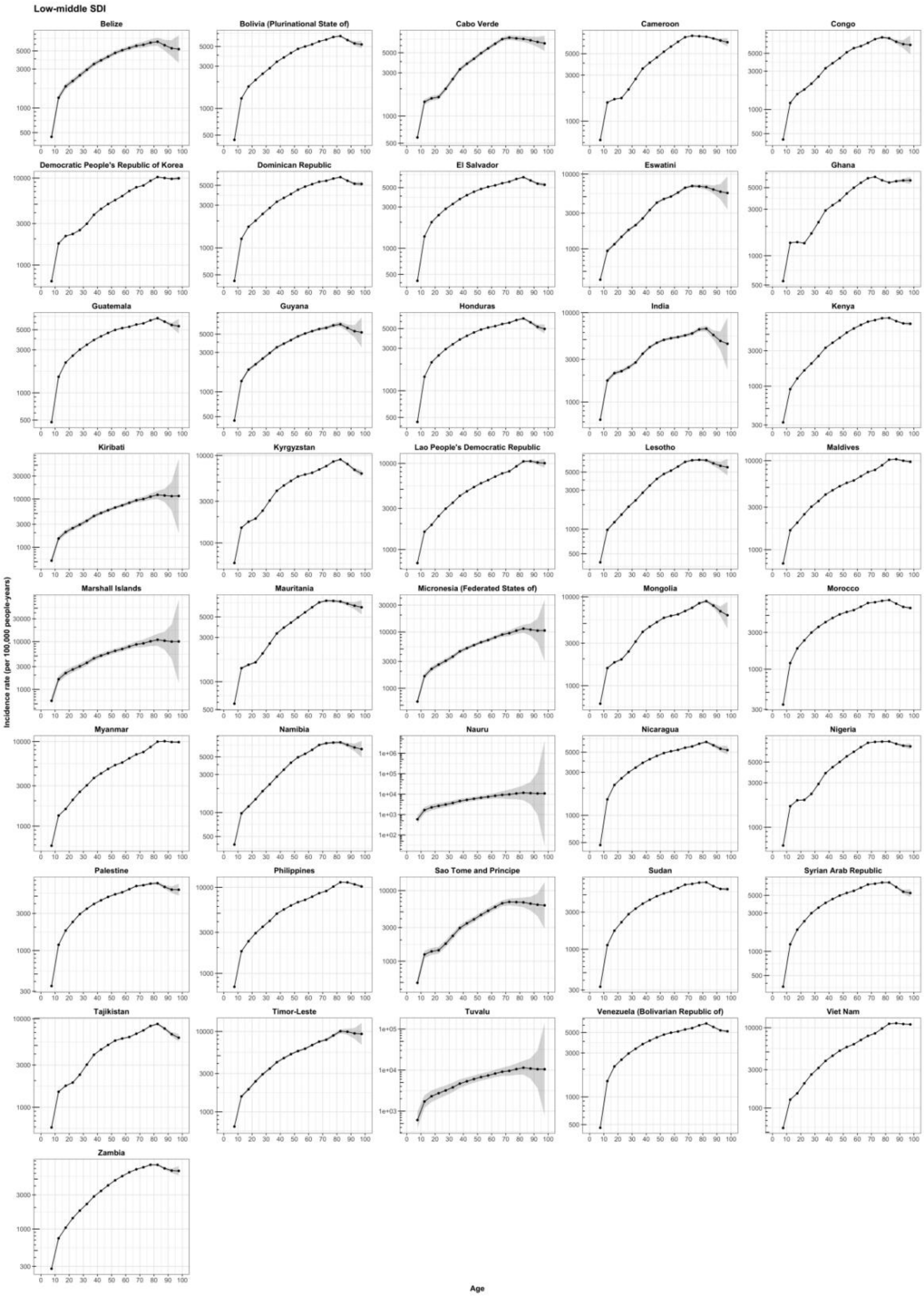

Figure S16. Age effects on low back pain incidence in low-middle SDI countries

Age effects indicate age-associated natural history and are shown by the fitted longitudinal age curves of incidence (per 100000 person-years) adjusted for period deviations, with the dots and shaded areas denoting incidence rates with 95% CIs. SDI=Socio-demographic Index.

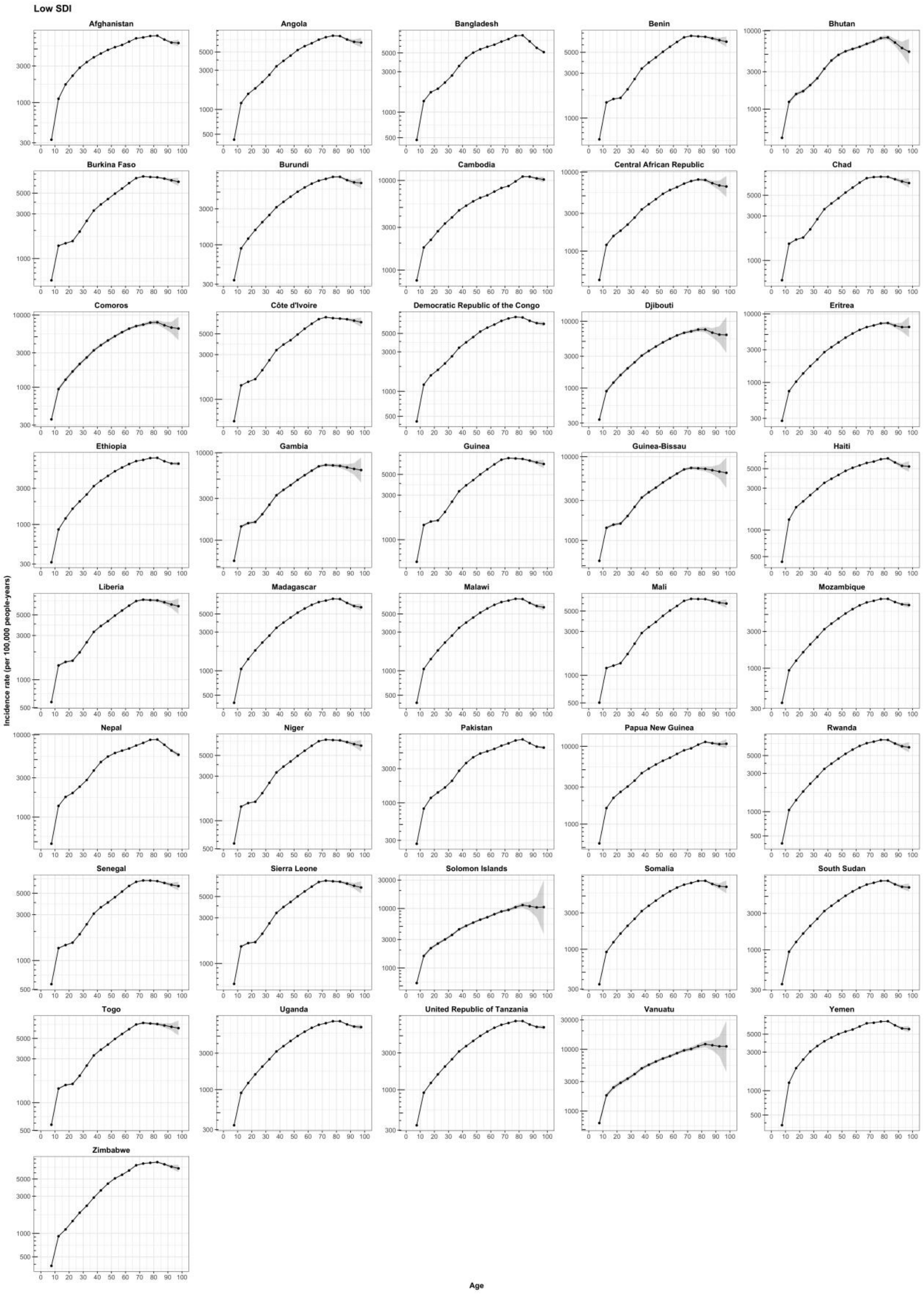

Figure S17. Age effects on low back pain incidence in low-SDI countries

Age effects indicate age-associated natural history and are shown by the fitted longitudinal age curves of incidence (per 100000 person-years) adjusted for period deviations, with the dots and shaded areas denoting incidence rates with 95% CIs. SDI=Socio-demographic Index.

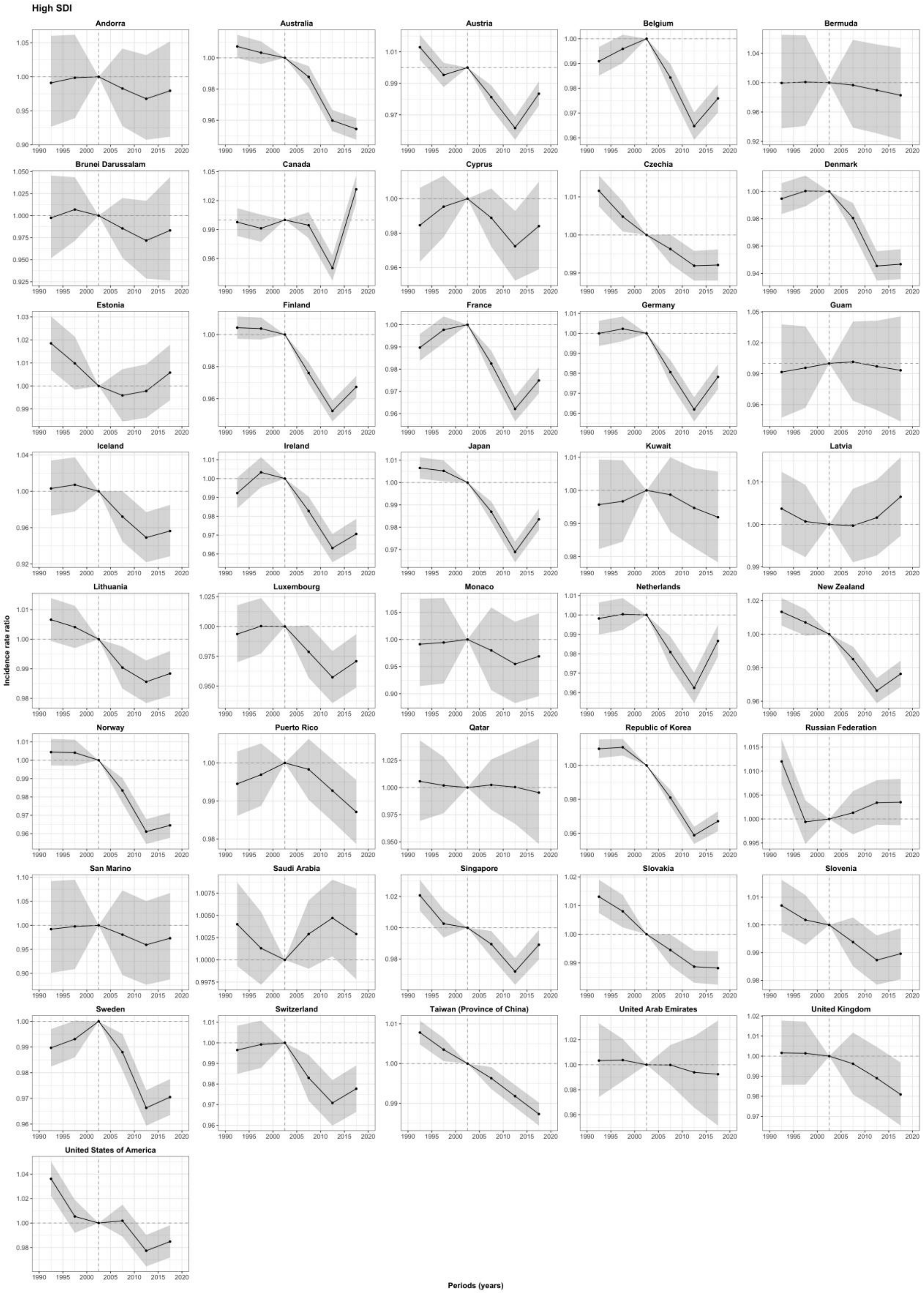

Figure S18. Period effects on low back pain incidence in high-SDI countries

Period effects are shown by the relative risk of incidence (incidence rate ratio) for each period from 1990-1994 to 2015-2019, with the dots and shaded areas representing rate ratios and 95% CIs for a given period relative to the referent period

(2000-2005). SDI=Socio-demographic Index.

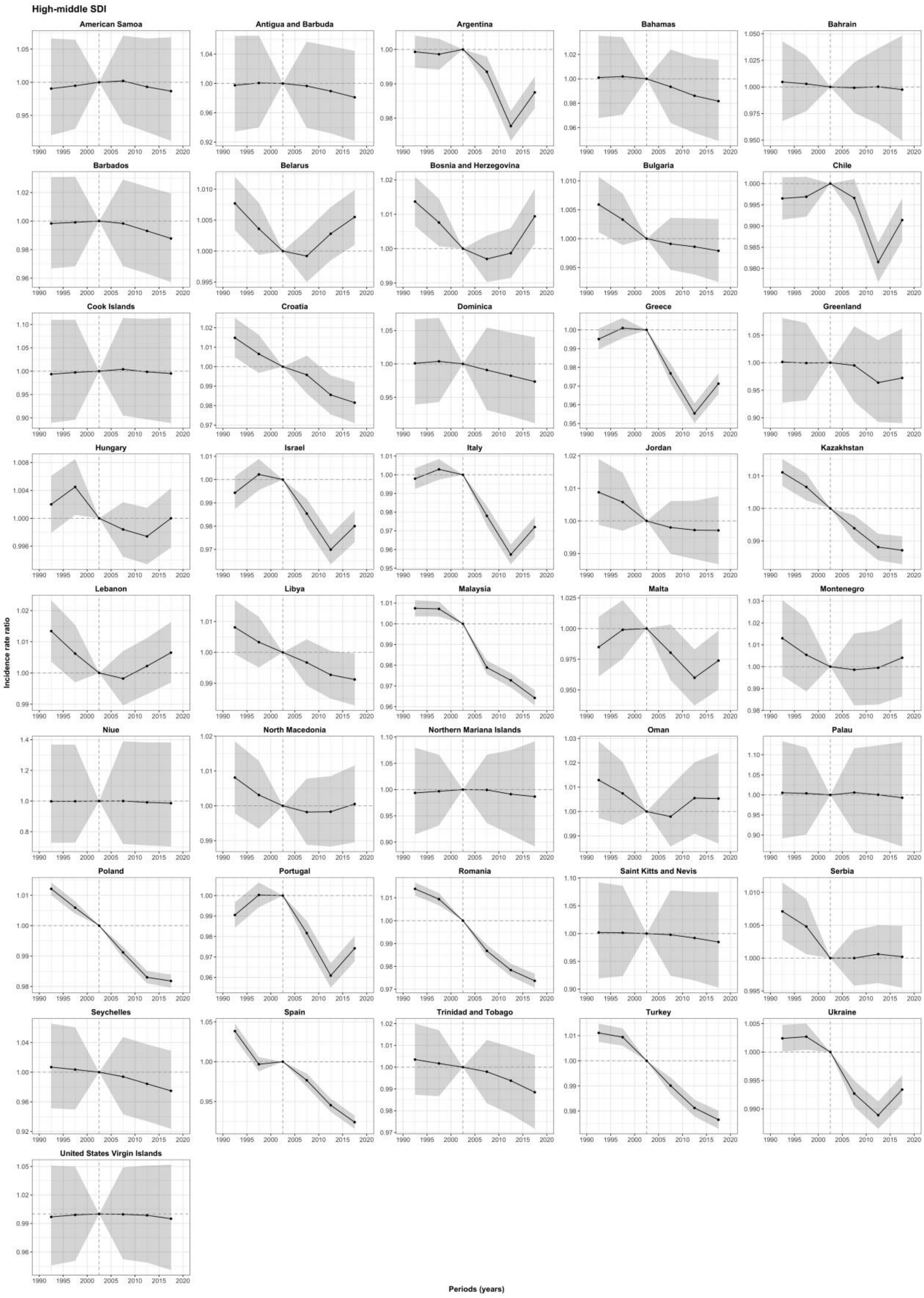

Figure S19. Period effects on low back pain incidence in high-middle SDI countries

Period effects are shown by the relative risk of incidence (incidence rate ratio) for

each period from 1990-1994 to 2015-2019, with the dots and shaded areas representing rate ratios and 95% CIs for a given period relative to the referent period (2000-2005). SDI=Socio-demographic Index.

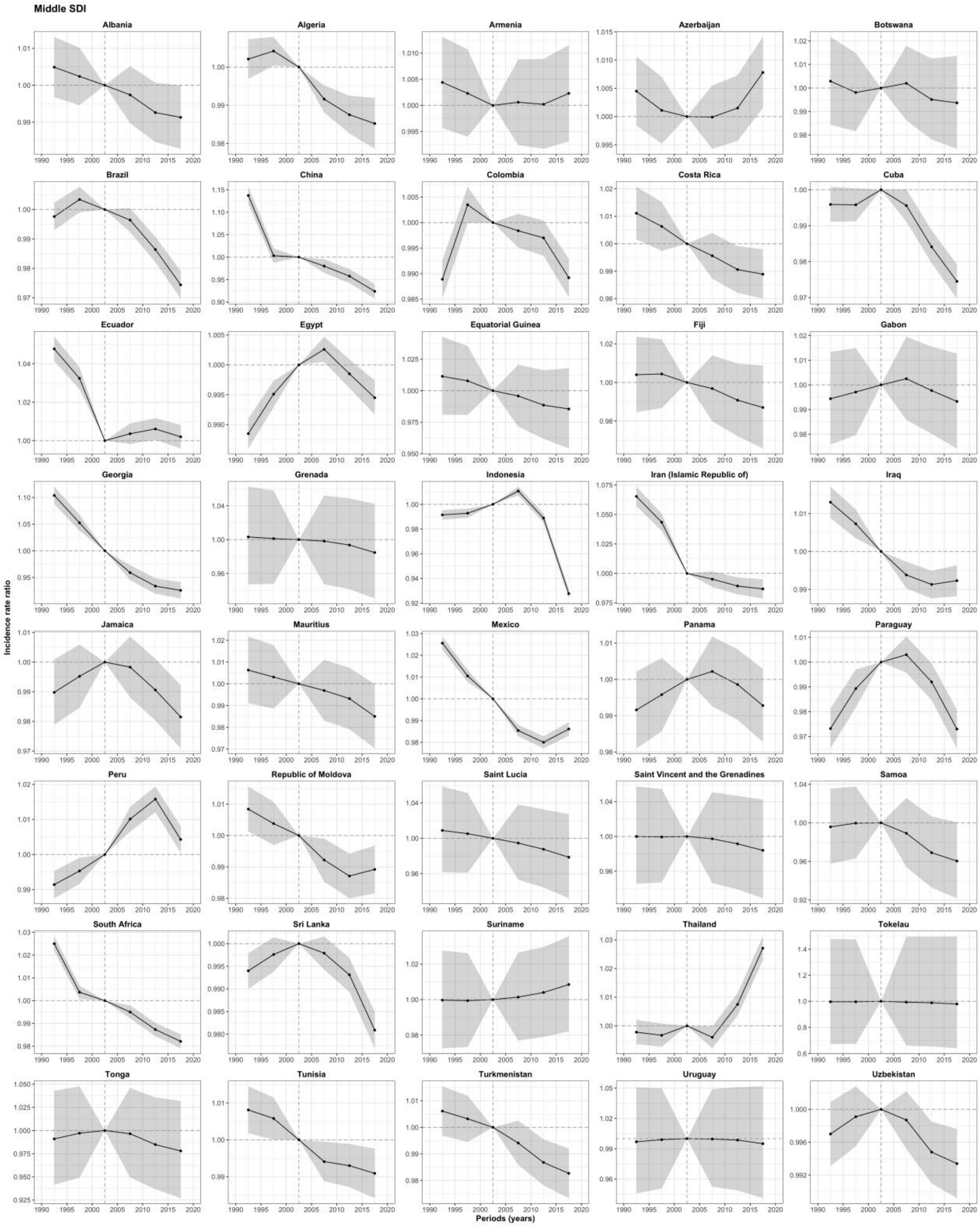

Figure S20. Period effects on low back pain incidence in middle-SDI countries

Period effects are shown by the relative risk of incidence (incidence rate ratio) for each period from 1990-1994 to 2015-2019, with the dots and shaded areas representing rate ratios and 95% CIs for a given period relative to the referent period (2000-2005). SDI=Socio-demographic Index.

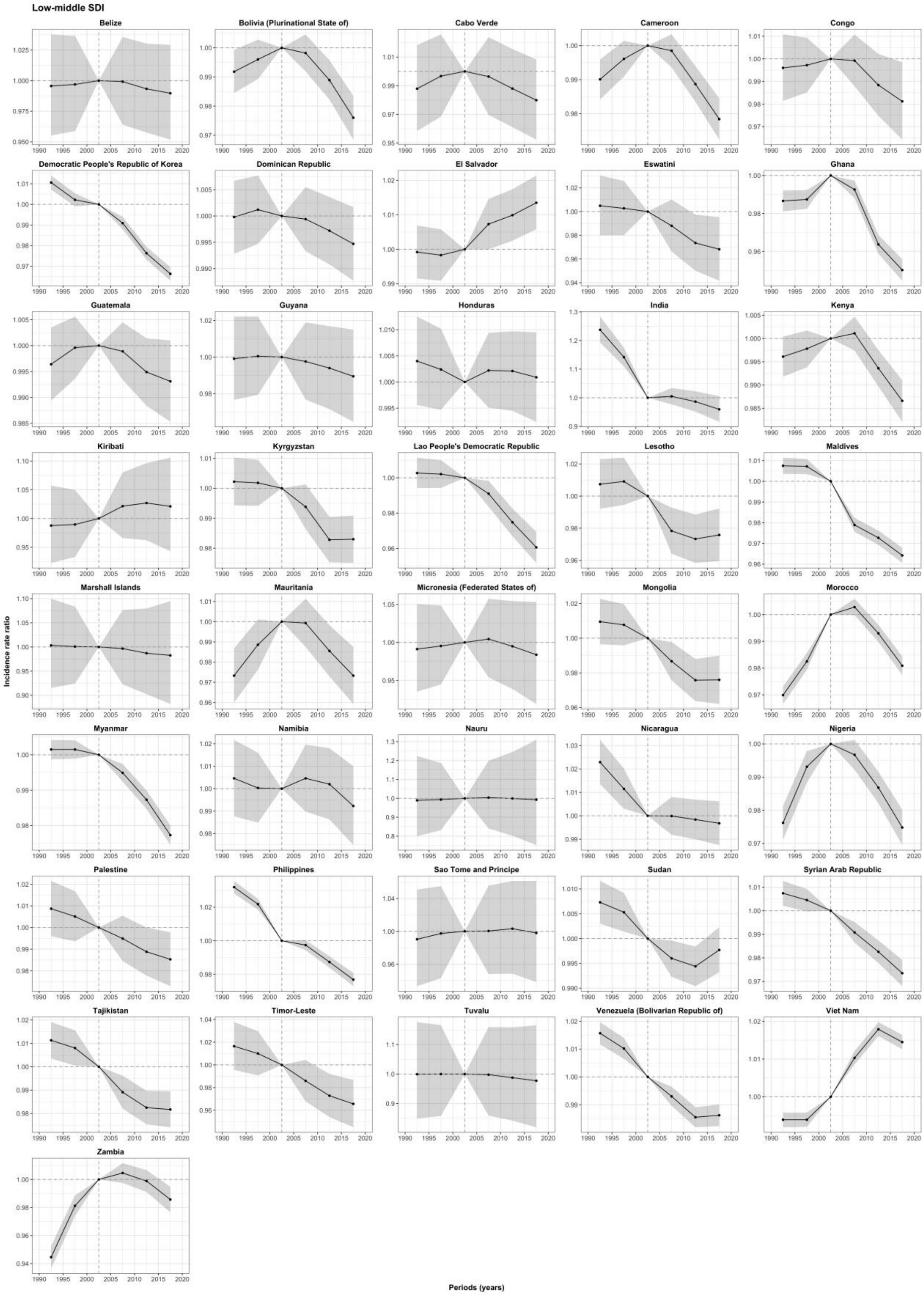

Figure S21. Period effects on low back pain incidence in low-middle SDI countries

Period effects are shown by the relative risk of incidence (incidence rate ratio) for each period from 1990-1994 to 2015-2019, with the dots and shaded areas

representing rate ratios and 95% CIs for a given period relative to the referent period (2000-2005). SDI=Socio-demographic Index.

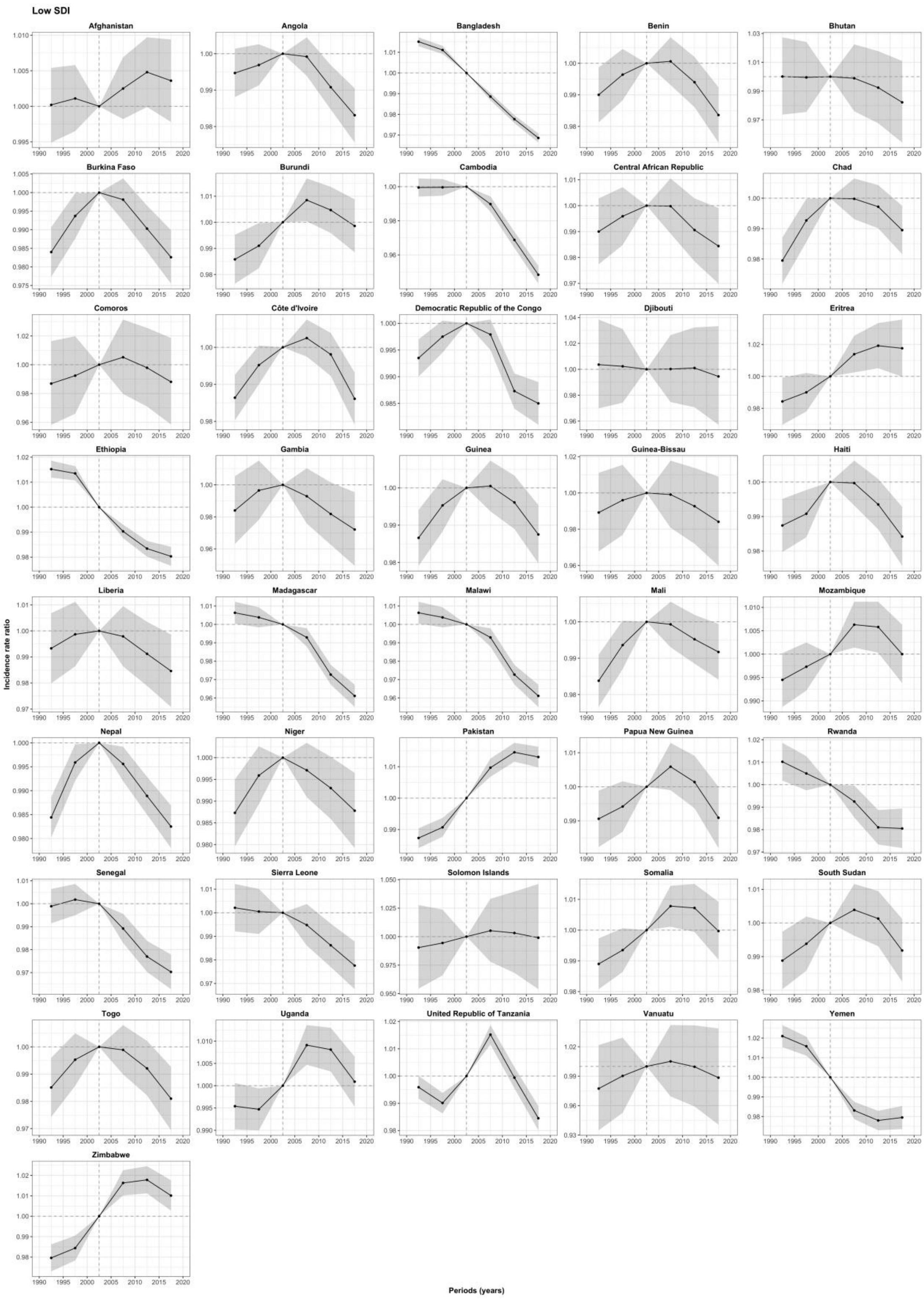

Figure S22. Period effects on low back pain incidence in low-SDI countries

Period effects are shown by the relative risk of incidence (incidence rate ratio) for

each period from 1990-1994 to 2015-2019, with the dots and shaded areas representing rate ratios and 95% CIs for a given period relative to the referent period (2000-2005). SDI=Socio-demographic Index.

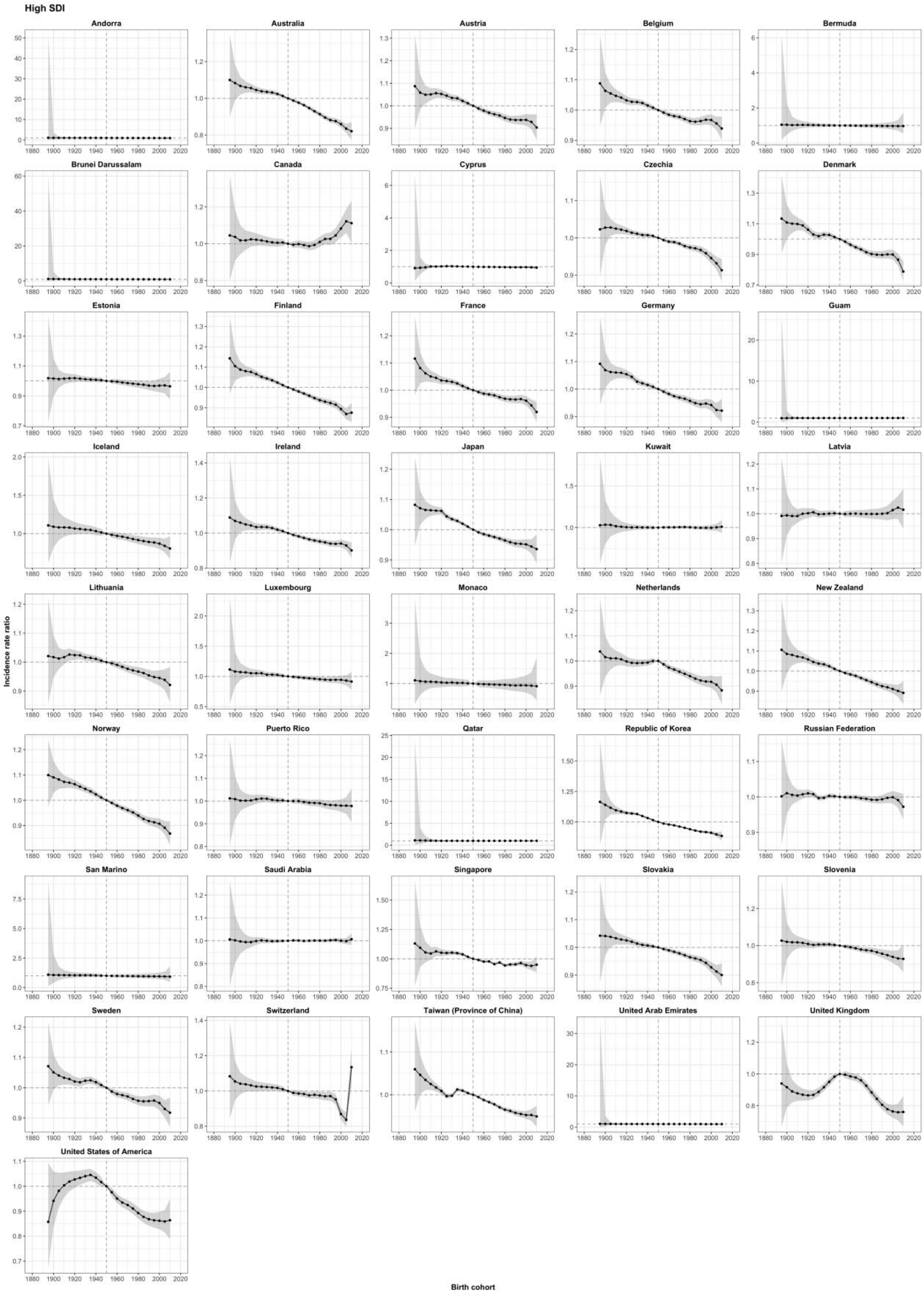

Figure S23. Cohort effects on low back pain incidence in high-SDI countries

Cohort effects are shown by the relative risk of incidence (incidence rate ratio) for each birth cohort from 1895 to 2010, with the dots and shaded areas represent rate ratios and 95% CIs for a given cohort relative to the referent 1950 cohort. SDI=Socio-demographic Index.

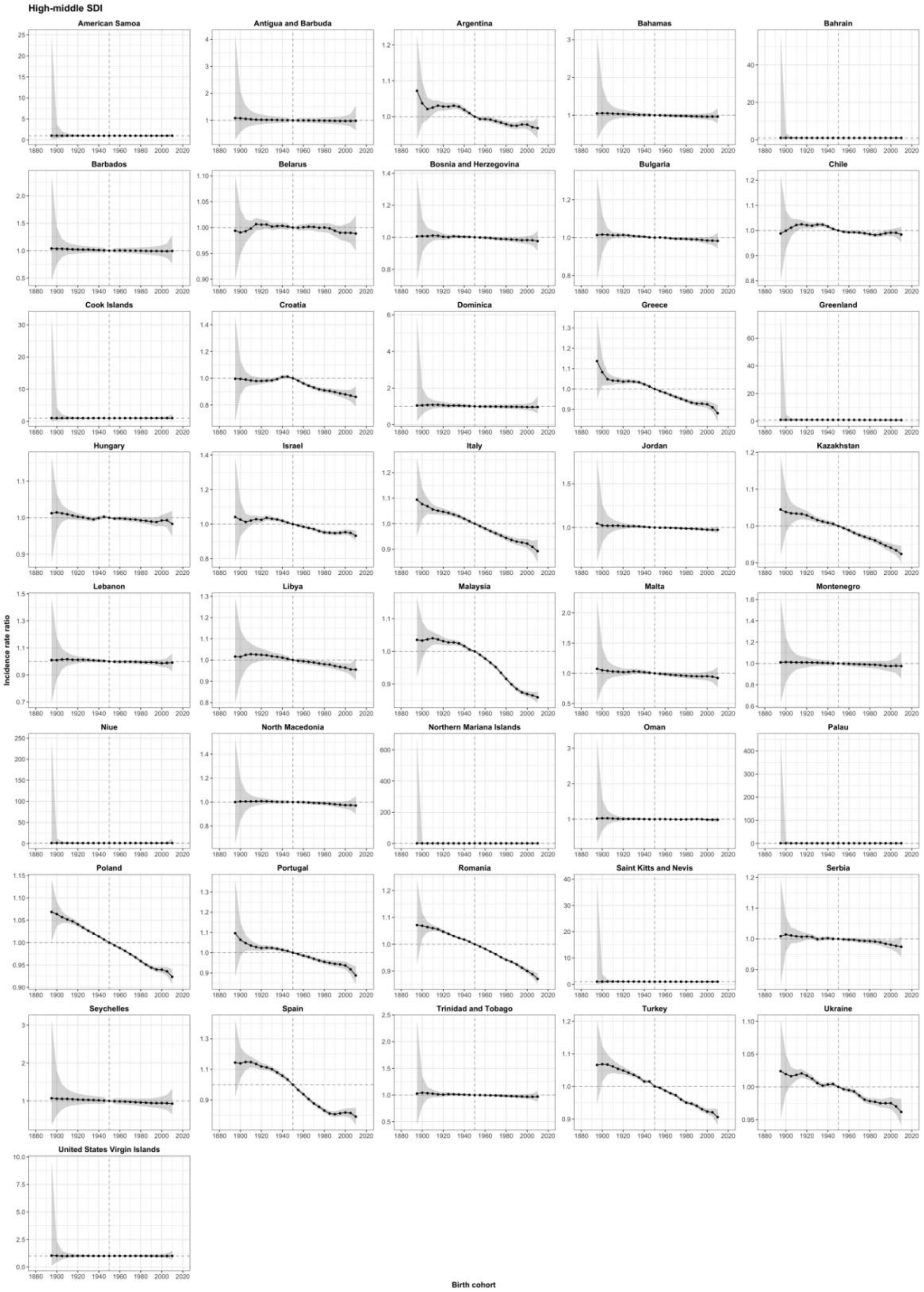

Figure S24. Cohort effects on low back pain incidence in high-middle SDI countries

Cohort effects are shown by the relative risk of incidence (incidence rate ratio) for each birth cohort from 1895 to 2010, with the dots and shaded areas represent rate ratios and 95% CIs for a given cohort relative to the referent 1950 cohort. SDI=Socio-demographic Index.

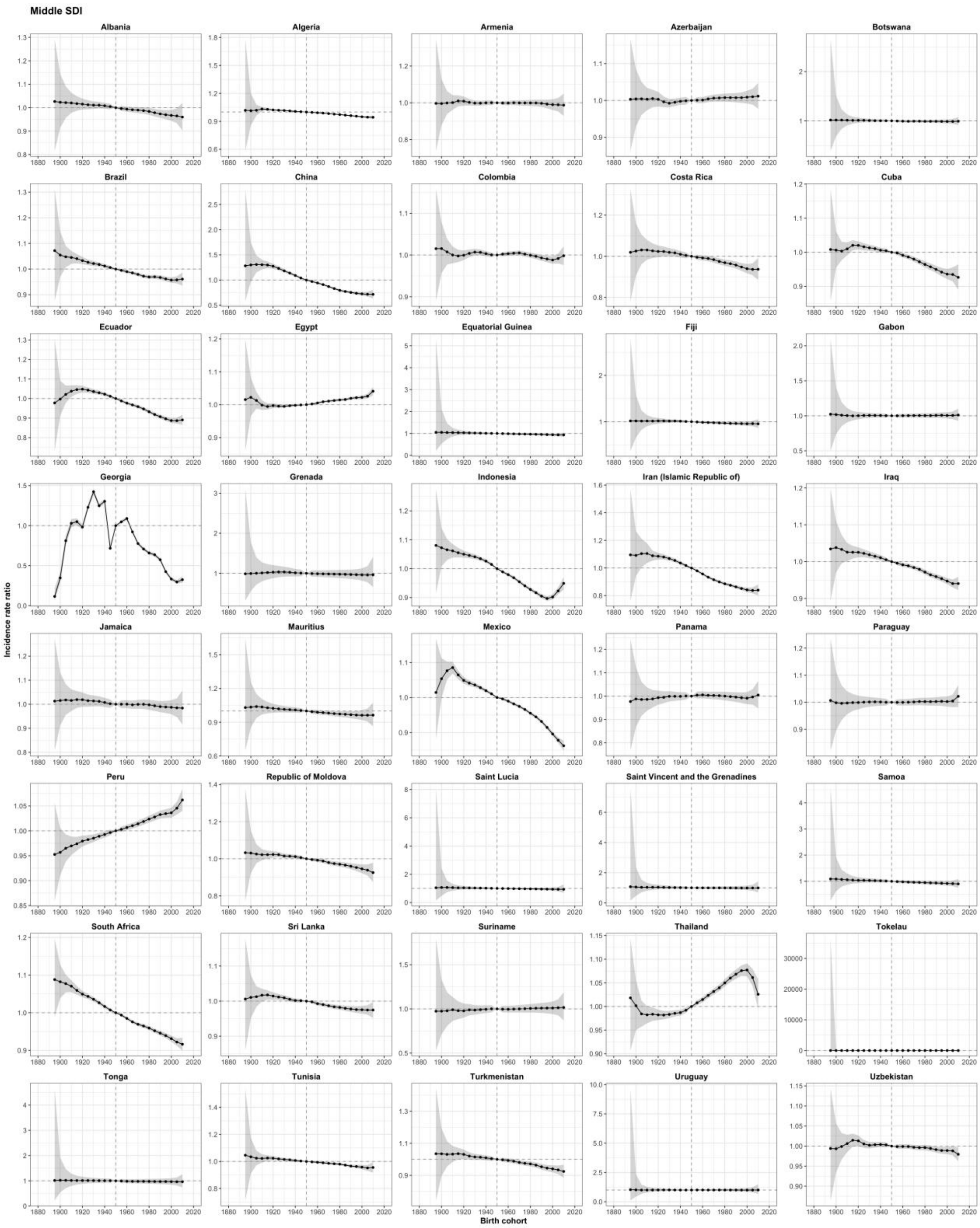

Figure S25. Cohort effects on low back pain incidence in middle-SDI countries

Cohort effects are shown by the relative risk of incidence (incidence rate ratio) for each birth cohort from 1895 to 2010, with the dots and shaded areas represent rate ratios and 95% CIs for a given cohort relative to the referent 1950 cohort.

SDI=Socio-demographic Index.

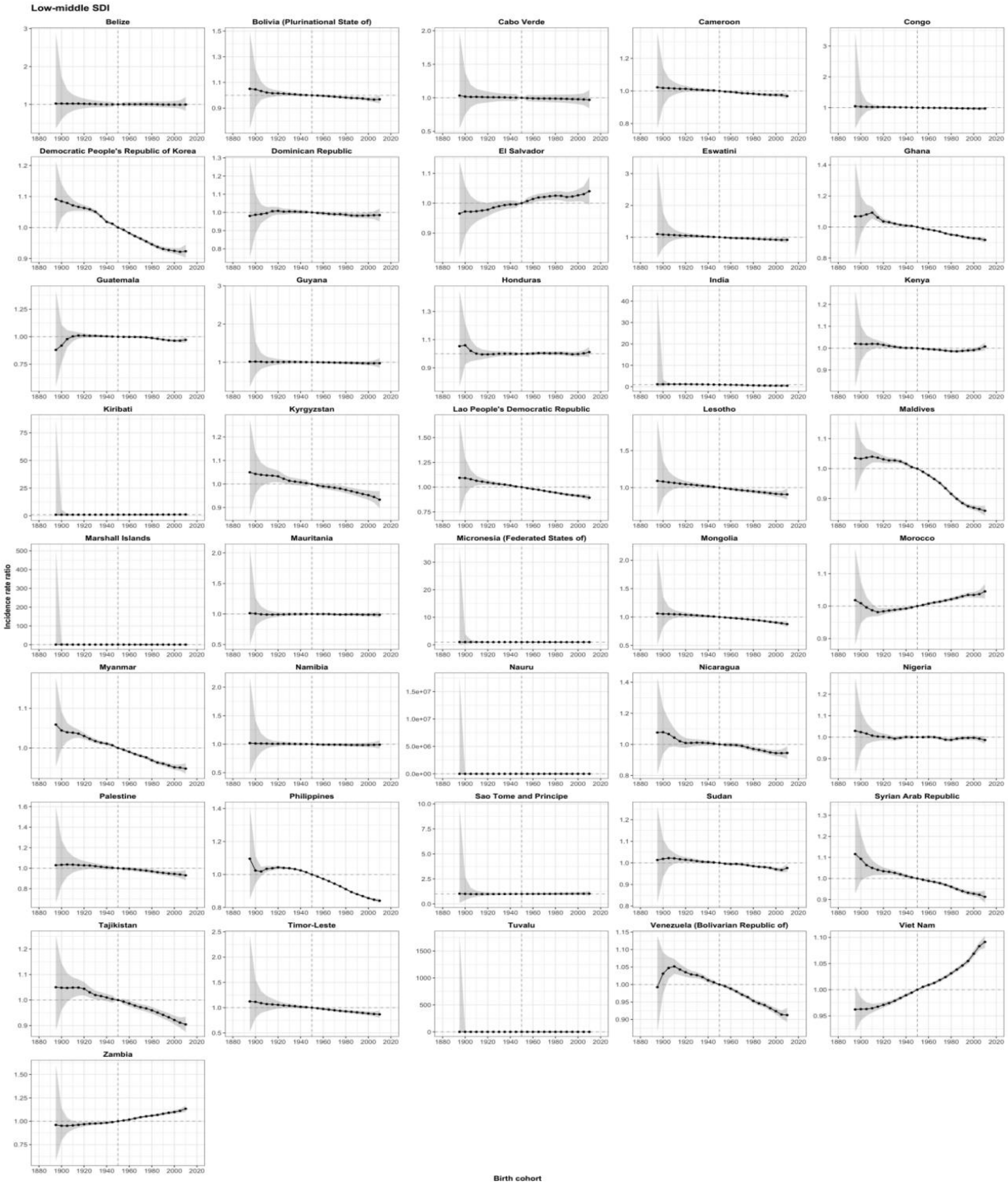

Figure S26. Cohort effects on low back pain incidence in low-middle SDI countries

Cohort effects are shown by the relative risk of incidence (incidence rate ratio) for each birth cohort from 1895 to 2010, with the dots and shaded areas represent rate ratios and 95% CIs for a given cohort relative to the referent 1950 cohort. SDI=Socio-demographic Index.

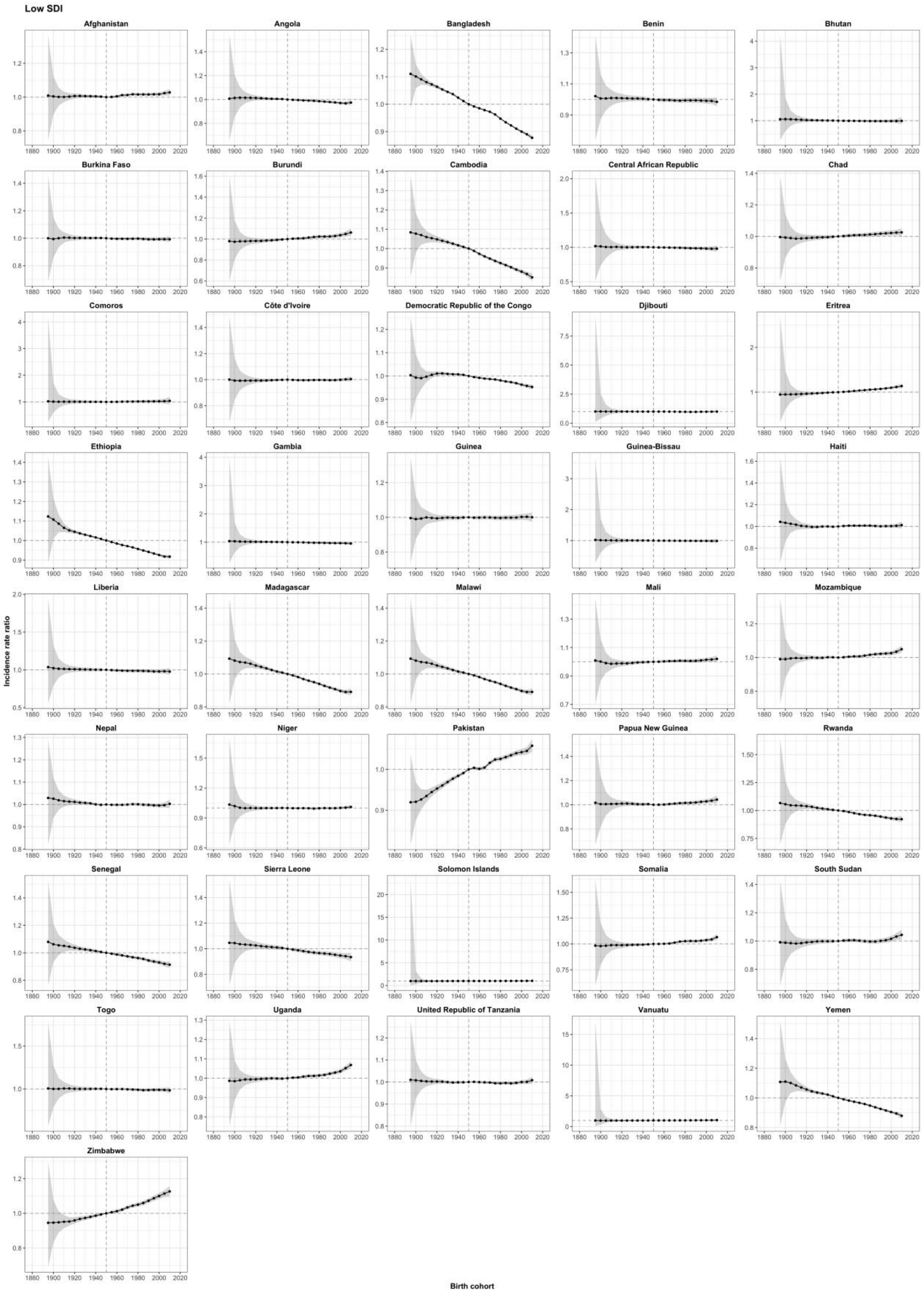

Figure S27. Cohort effects on low back pain incidence in low-SDI countries

Cohort effects are shown by the relative risk of incidence (incidence rate ratio) for each birth cohort from 1895 to 2010, with the dots and shaded areas represent rate ratios and 95% CIs for a given cohort relative to the referent 1950 cohort. SDI=Socio-demographic Index.
